# A trafficking regulatory subnetwork governs α_V_β_6_ integrin-HER2 cross-talk to control breast cancer invasion and drug resistance

**DOI:** 10.1126/sciadv.adk9944

**Published:** 2024-12-04

**Authors:** Horacio Maldonado, Marcel Dreger, Lara D. Bedgood, Theano Kyriakou, Katarzyna I. Wolanska, Megan E. Rigby, Valeria E. Marotta, Justine M. Webster, Jun Wang, Emma V. Rusilowicz-Jones, John F. Marshall, Judy M. Coulson, Iain R. Macpherson, Adam Hurlstone, Mark R. Morgan

**Affiliations:** ^1^ Institute of Systems, Molecular & Integrative Biology, University of Liverpool, Crown Street, Liverpool L69 3BX, UK.; ^2^ Division of Immunology, Immunity to Infection and Respiratory Medicine, Faculty of Biology, Medicine & Health, University of Manchester, Oxford Road, Manchester M13 9PT, UK.; ^3^ Centre for Tumour Biology, Barts Cancer Institute, Queen Mary University London, John Vane Science Centre, Charterhouse Square, London EC1M 6BQ, UK.; ^4^ Wolfson Wohl Cancer Research Centre, School of Cancer Sciences, University of Glasgow, Garscube Estate, Glasgow G61 1QH, UK.

## Abstract

HER2 and α_V_β_6_ integrin are independent predictors of breast cancer survival and metastasis. We identify an α_V_β_6_/HER2 cross-talk mechanism driving invasion, which is dysregulated in drug-resistant HER2+ breast cancer cells. Proteomic analyses reveal ligand-bound α_V_β_6_ recruits HER2 and a trafficking subnetwork, comprising guanosine triphosphatases RAB5 and RAB7A and the Rab regulator guanine nucleotide dissociation inhibitor 2 (GDI2). The RAB5/RAB7A/GDI2 functional module mediates direct cross-talk between α_V_β_6_ and HER2, affecting receptor trafficking and signaling. Acute exposure to trastuzumab increases recruitment of the subnetwork to α_V_β_6_, but trastuzumab resistance decouples GDI2 recruitment. GDI2, RAB5, and RAB7A cooperate to regulate migration and transforming growth factor–β activation to promote invasion. However, these mechanisms are dysregulated in trastuzumab-resistant cells. In patients, *RAB5A*, *RAB7A*, and *GDI2* expression correlates with patient survival and α_V_β_6_ expression predicts relapse following trastuzumab treatment. Thus, the RAB5/RAB7A/GDI2 subnetwork regulates α_V_β_6_-HER2 cross-talk to drive breast cancer invasion but is subverted in trastuzumab-resistant cells to drive α_V_β_6_-independent and HER2-independent tumor progression.

## Teaser

Control of αVβ6 integrin and HER2 trafficking drives breast cancer invasion, drug resistance, and therapeutic response.

## INTRODUCTION

Human epidermal growth factor receptor 2 (HER2/ERBB2) is an oncogenic receptor tyrosine kinase (RTK) that drives cancer cell proliferation and survival (*1*–*3*). HER2 is overexpressed in 25 to 30% of breast cancers and promotes invasion and metastasis (*4*–*9*). Consequently, therapeutic strategies targeting HER2 have been developed and trastuzumab, a humanized monoclonal antibody (mAb) targeting the extracellular domain of HER2, has substantially improved outcomes for patients with HER2-positive (HER2+) breast cancer (*9*–*11*). However, the benefits of trastuzumab are limited substantially by innate or acquired drug resistance (*12*).

The adhesion receptor α_V_β_6_ integrin is an epithelial integrin, expressed at very low levels in normal tissue but substantially up-regulated in a range of epithelial cancers where it acts as a prognostic indicator (*13*–*20*). This is particularly the case in HER2+ breast cancer, where expression of α_V_β_6_ is a predictor of poor survival and metastasis (*17*, *20*). Integrin α_V_β_6_ is a proinvasive receptor, characterized by the ability to promote cell motility, protease-mediated extracellular matrix (ECM) degradation, and mechanical activation of transforming growth factor–β1/3 (TGFβ1/3) (*21*–*24*). Thus, coordination of α_V_β_6_-dependent mechanisms promotes remodeling of the tumor microenvironment and cancer cell invasion, rendering α_V_β_6_ integrin a multifunctional receptor driving cancer progression.

While HER2 and α_V_β_6_ integrin are independent prognostic indicators in breast cancer, patients with HER2+ breast cancer and high α_V_β_6_ expression exhibit reduced survival compared with patients with low α_V_β_6_ expression (*17*). Moreover, simultaneous targeting of α_V_β_6_ and HER2 effectively eliminates tumors in mouse xenograft models of trastuzumab-sensitive breast cancer, suggesting that cotargeting α_V_β_6_ and HER2 represents a potential therapeutic avenue for treating breast cancer (*17*).

Mounting evidence suggests that numerous mechanisms coordinate cross-talk between RTKs and integrins to control cell adhesion, motility, proliferation, invasion, and drug resistance (*25*–*31*). To effectively exploit adhesion receptors and RTKs therapeutically, it is essential to understand how their signaling networks are integrated and how cross-talk mechanisms coordinate invasion and the response to targeted molecular therapeutics. However, to date, no evidence of direct cross-talk between α_V_β_6_ and HER2 exists.

Integrin receptors couple the extracellular microenvironment with intracellular cytoskeletal and signaling machinery, at integrin-associated adhesion complexes (IACs), to coordinate mechanochemical signaling and control a wide range of cellular functions (*32*–*35*). Integrin-ECM engagement triggers recruitment of a complex and dynamic network of hundreds of proteins, typically termed the “adhesome” (*36*–*38*). To dissect the regulatory processes coordinating α_V_β_6_ function in HER2+ breast cancer, we used global proteomic strategies to analyze the specific α_V_β_6_ adhesome in HER2+ breast cancer cells. We demonstrate that the α_V_β_6_ adhesome is enriched for HER2 and a trafficking regulatory subnetwork comprising the small guanosine triphosphatases (GTPases) RAB5 and RAB7A and the Rab regulator GDI2 (guanine nucleotide dissociation inhibitor 2; also known as RabGDIβ). Furthermore, the composition of this subnetwork is dynamically and differentially modulated by treatment with trastuzumab or induction of acquired trastuzumab resistance.

HER2 internalization and suppression of recycling represents a key mechanism by which HER2+ breast cancer cells may adapt to evade trastuzumab exposure and is a potential biomarker of drug sensitivity (*39*, *40*). The subcellular distribution of HER2 correlates with anti-HER2 drug resistance, with resistant cell lines exhibiting more intracellular HER2, compared to therapy-sensitive cells, displaying predominantly plasma membrane–localized HER2 (*41*). We demonstrate that the GDI2/RAB5/RAB7A trafficking regulatory subnetwork mediates direct cross-talk between α_V_β_6_ and HER2, affecting α_V_β_6_ expression, HER2 trafficking and signaling and, consequently, HER2 availability at the cell surface. Furthermore, this mechanism is dysregulated when cells acquire trastuzumab resistance, affecting TGFβ activation, cell invasion, and dissemination. Expression of components of the trafficking regulatory subnetwork correlates with patient survival, and α_V_β_6_ expression predicts therapeutic response following breast cancer relapse. Together, these results further our understanding of how HER2+ cancer cells evade trastuzumab exposure and identify putative molecular targets for future therapeutic development.

## RESULTS

### Integrin α_V_β_6_ recruits HER2 and a trafficking regulatory subnetwork

**Fig. 1.**
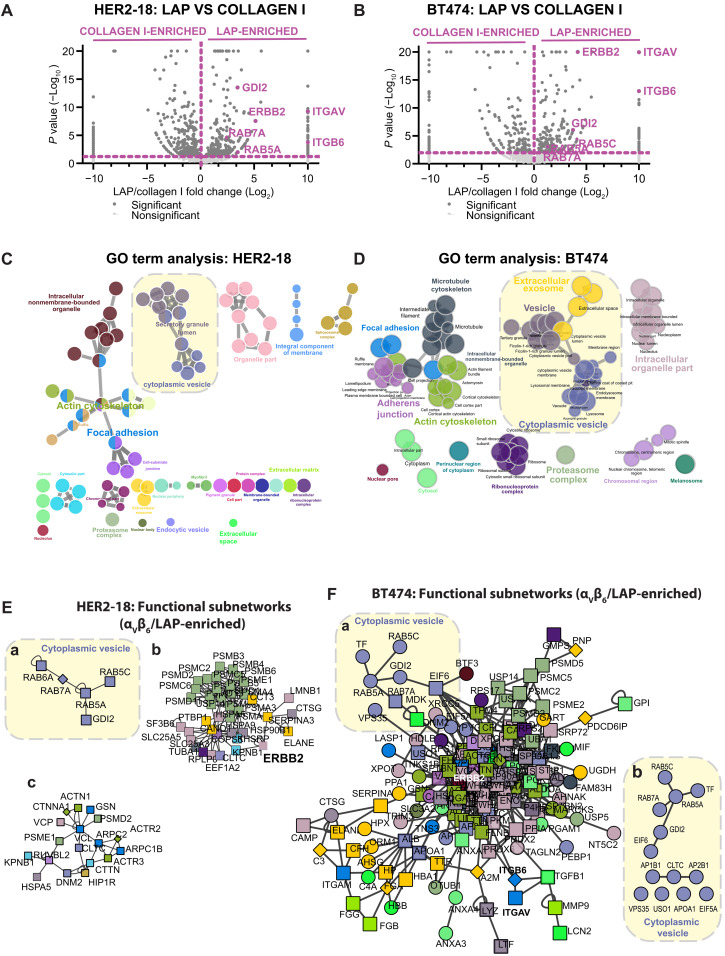
Integrin α_V_β_6_ recruits HER2 and a trafficking regulatory subnetwork comprising RAB5/RAB7A/GDI2 in HER2+ breast cancer cells. IAC enrichment coupled with free-label MS was used to define proteins specifically recruited to ligand-bound α_V_β_6_ in HER2+ breast cancer cell lines. (**A** and **B**) Volcano plots demonstrating enrichment of proteins identified on LAP (α_V_β_6_ integrin–selective ligand; right) and Coll-I (non-α_V_β_6_ integrin binding ligand; left) matrices in (A) HER2-18 and (B) BT474 cells. Statistical analysis: Fisher’s exact test; quantitative method: weighted spectra; significance level: *P* < 0.05. Significant proteins (dark gray); nonsignificant proteins (light gray); proteins of interest highlighted in purple. (**C** and **D**) Visual representation of ClueGO cellular compartment GO analyses of proteins significantly enriched on LAP in comparison with Coll-I in (C) HER2-18 and (D) BT474 cells. Colors represent specific merged GO term groups, node size represents level of significance of each GO term, and clustering and edge length represent functionally grouped networks based on kappa score. Yellow boxes highlight the cytoplasmic vesicle GO term cluster. (**E** and **F**) Top functional subnetworks of proteins significantly enriched on LAP in comparison with Coll-I in (E) HER2-18 and (F) BT474 cells, identified using the OH-PIN algorithm. Colors represent the primary cellular compartment GO term associated with each protein as identified in (C) and (D), respectively. Yellow boxes [(Ea) and (Fa)] highlight the clusters of proteins related to GO term cytoplasmic vesicle, in the top functional subnetwork isolated from each cell line. [(Eb) and (Ec)] Second and third most significant subnetworks in HER2-18 cells. (Fb) All proteins in the cytoplasmic vesicle GO term within the primary functional subnetwork in α_V_β_6_ integrin/LAP-enriched IACs in BT474 cells. All MS data represent three independent experiments. See also figs. S1 (HER2-18) and S3 (BT474) and data files S1 and S2.

To understand the role of α_V_β_6_ integrin in HER2+ breast cancer progression, we sought to define the composition of the signaling networks recruited to ligand-bound α_V_β_6_, “the α_V_β_6_ integrin adhesome,” in HER2+ breast cancer cells. IAC enrichment coupled with quantitative proteomic analysis was used in two HER2+ breast cancer cell lines: HER2-18 and BT474 cells (*42*, *43*). IACs were isolated from cells plated on latency-associated peptide (LAP), fibronectin (FN), or collagen-I (Coll-I) and subjected to label-free analysis by mass spectrometry (MS). LAP is an α_V_β_6_-selective ligand; FN can engage multiple integrins including, but not exclusively, α_V_β_6_; and Coll-I was a non–α_V_β_6_-binding negative control capable of binding different classes of integrin (*21*, *44*, *45*). Proteomic analysis and immunofluorescence confirmed the heterodimer specificity of α_V_β_6_ binding to LAP as αVβ6 was the primary RGD-binding heterodimer recruited to the cell-matrix interface in cells plated on LAP (Fig. 1, A and B, and figs. S1, A to E, and S2). Pairwise analysis of protein networks recruited to IACs on different substrates allowed identification of proteins selectively recruited to ligand-engaged α_V_β_6_ integrin and delineation of the α_V_β_6_-associated adhesome (Fig. 1, A and B, and figs. S1, A to E, and S3, A to E).

To gain mechanistic insight from the proteomic datasets, ontological and functional enrichment analyses were performed to identify overrepresentation of cellular compartment gene ontology (GO) terms (Fig. 1, C and D) and functional subnetworks recruited to ligand-bound α_V_β_6_ (Fig. 1, E and F), respectively. As expected, the primary cluster identified by GO analysis represented terms typically associated with IAC function (e.g., “focal adhesion,” “actin cytoskeleton,” and “intracellular nonmembrane bounded organelle”). However, the second most statistically significant cluster identified through GO analysis, in both HER2-18 and BT474 cells, was associated predominantly with intracellular trafficking pathways (e.g., “cytoplasmic vesicle lumen,” “secretory granule lumen,” and “vesicle”) (Fig. 1, C and D). Functional enrichment analysis revealed that the highest confidence subnetwork recruited to ligand-bound α_V_β_6_ in HER2-18 cells was an endosomal trafficking module, comprising GDI2, RAB5, RAB7A, and RAB6A (Fig. 1Ea and figs. S4A and S5A). In addition, the second and third highest confidence subnetworks contained HER2 and were related to proteasomal regulation (Fig. 1Eb) and cytoskeletal regulation (Fig. 1Ec). Similarly, in BT474 cells, HER2 and the key components of the RAB5/RAB7A/GDI2 trafficking regulatory subnetwork were contained within the dominant functional subnetwork in α_V_β_6_-dependent IACs (Fig. 1F). Together, these data suggested that HER2 is recruited to sites of ligand-bound α_V_β_6_ integrin and that endosomal trafficking regulators are also recruited to the α_V_β_6_-dependent adhesion environment (the α_V_β_6_ adhesome).

**Fig. 2.**
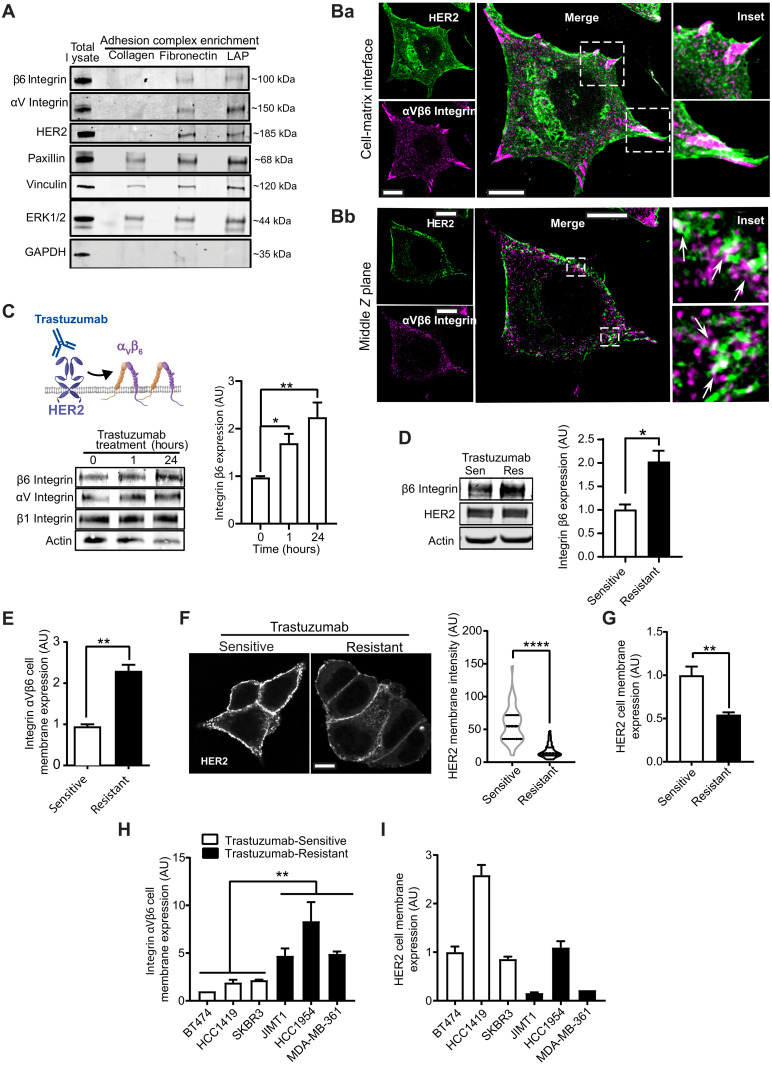
HER2 and integrin α_V_β_6_ colocalize and trastuzumab regulates HER2 and integrin α_V_β_6_ expression. (**A**) Immunoblot analysis of β_6_ integrin, α_V_ integrin, HER2, paxillin, vinculin, ERK1/2, and GAPDH protein levels in IACs isolated from BT474 cells on LAP, FN, and Coll-I (*N* = 3). (**B**) HER2 (magenta) and β_6_ integrin (green) immunofluorescence in BT474 cells. Two *Z* planes of the same cell: (Ba) cell-matrix interface and (Bb) middle *Z* plane. Dashed boxes: insets. Arrows: membrane-proximal vesicular HER2/β_6_ colocalization; scale bars, 10 μm. (**C**) Immunoblot analysis of integrin β_6_, α_V_, and β_1_ and actin (loading control) expression in BT474 cells treated with trastuzumab (10 μg/ml) for 0, 1, and 24 hours (*N* = 3). One-way ANOVA, Šídák’s multiple comparison test. (**D**) Immunoblot analysis of total β_6_ integrin and HER2 expression, normalized to actin, in trastuzumab-sensitive (Sen) and trastuzumab-resistant (Res) BT474 cells (*N* = 3). Two-sided *t* test, Welch’s correction. (**E**) Flow cytometry analysis of cell surface α_V_β_6_ integrin expression in trastuzumab-sensitive and trastuzumab-resistant BT474 cells [mean fluorescence intensity (MFI) normalized to Sen cells, *N* = 4]. Two-sided *t* test. (**F**) Fluorescence analysis of HER2 expression at the plasma membrane of trastuzumab-sensitive and trastuzumab-resistant nonpermeabilized BT474 cells surface labeled with FITC-conjugated HER2 affibody (*N* = 3; 44 to 52 cells per condition); scale bar, 10 μm. Two-sided Mann-Whitney test. (**G**) Flow cytometry analysis of HER2 cell surface expression in trastuzumab-sensitive (Sen) and trastuzumab-resistant (Res) BT474 cells (MFI normalized to Sen cells, *N* = 3). Two-sided *t* test. (**H** and **I**) Cell surface expression of α_V_β_6_ integrin (H) and HER2 (I) by flow cytometry in HER2+ breast cancer cells that are endogenously sensitive (white) or resistant (black) to trastuzumab (*N* = 4). One-way ANOVA, Dunnett’s multiple comparison test. [(C) to (I)] Data shown are arbitrary units (AU) normalized to control means (untreated trastuzumab-sensitive BT474 cells) ± SEM. Statistical significance: *P < 0.05; **P < 0.01; ****P < 0.0001.

Recruitment of HER2 to α_V_β_6_-dependent IACs was confirmed by immunoblotting IACs isolated on LAP and FN, in comparison with those enriched on Coll-I (Fig. 2A). The specificity of isolation was confirmed by the presence of β_6_ and α_V_ integrin subunits on LAP and FN, recruitment of vinculin and paxillin on all three integrin-binding ligands, and the absence of glyceraldehyde-3-phosphate dehydrogenase (GAPDH), a nonadhesion protein (Fig. 2A). Enrichment of GDI2, RAB5, and RAB7A were also confirmed by immunoblotting (figs. S4A and S5A). Recruitment and colocalization of HER2 to sites of α_V_β_6_ ligand engagement, at the cell-matrix interface, were further confirmed by immunofluorescence (Fig. 2Ba). However, α_V_β_6_-HER2 colocalization was also observed in punctal vesicle–like structures in the cytoplasmic region of cells (Fig. 2Bb). Further analyses revealed that α_V_β_6_ ligand binding promotes colocalization of α_V_β_6_ integrin, HER2, and the Rab regulator GDI2 (fig. S4, Ba to Bc). Moreover, engagement of cells on LAP increased colocalization of RAB5A and RAB7A on vesicular structures (fig. S5Ba). Thus, ligand binding of integrin α_V_β_6_ modulates the subcellular distribution of HER2 and components of the RAB5/RAB7A/GDI2 trafficking regulatory subnetwork. These data led us to focus on whether there was a functional link between α_V_β_6_ and HER2 and whether this involved receptor trafficking mechanisms.

### Trastuzumab modulates α_V_β_6_ integrin expression and HER2 plasma membrane bioavailability

Proteomic, immunoblot, and imaging analyses suggested that engagement of α_V_β_6_ integrin recruits the therapeutically tractable RTK HER2 and molecular machinery associated with receptor trafficking mechanisms (Fig. 1 and figs. S1, S4, and S5). While cotargeting of α_V_β_6_ and HER2 inhibits tumor growth in in vivo models of trastuzumab-sensitive breast cancer (*17*), to date, there is no evidence of direct cross-talk between the two receptors. Therefore, the identification of HER2 as a key component of α_V_β_6_-associated adhesions led us to investigate whether the functions of α_V_β_6_ and HER2 were functionally linked.

Trastuzumab is a key part of contemporary treatment regimens in patients with HER2+ breast cancer (*46*), but up to 70% exhibit resistance to the drug (*9*, *12*, *47*). Having demonstrated that ligand-engaged α_V_β_6_ recruits HER2, we tested what effect trastuzumab-mediated HER2 inhibition or trastuzumab resistance has on α_V_β_6_ integrin. To achieve this, we generated BT474 cells that were resistant to high concentrations of trastuzumab (BT474 Trastuzumab-Resistant) but still sensitive to the HER2-targeting tyrosine kinase inhibitor lapatinib and matched parental cells (BT474 Trastuzumab-Sensitive) (fig. S6, A and B). Relatively short-term trastuzumab treatment of trastuzumab-sensitive cells increased α_V_β_6_ expression (Fig. 2C). Moreover, induction of acquired trastuzumab resistance in BT474 cells was sufficient to increase total and cell surface α_V_β_6_ expression (Fig. 2, D and E). By contrast, trastuzumab resistance decreased the levels of HER2 at the cell surface while not affecting overall expression levels (Fig. 2, D, F, and G). Flow cytometry analysis of a panel of cell lines that are endogenously trastuzumab sensitive (BT474, HCC1419, and SKBR3) or trastuzumab resistant (JIMT1, HCC1954, and MDA-MB-361) revealed similar expression patterns, elevated cell surface α_V_β_6_ and reduced membrane-localized HER2 in trastuzumab-resistant cells (Fig. 2, H and I). Together, these data indicate that α_V_β_6_ and HER2 are functionally coupled and that trastuzumab exposure and resistance promote α_V_β_6_ expression while limiting expression of HER2 at the cell surface.

### Trastuzumab modulates recruitment of the trafficking regulatory subnetwork to α_V_β_6_ IACs

**Fig. 3.**
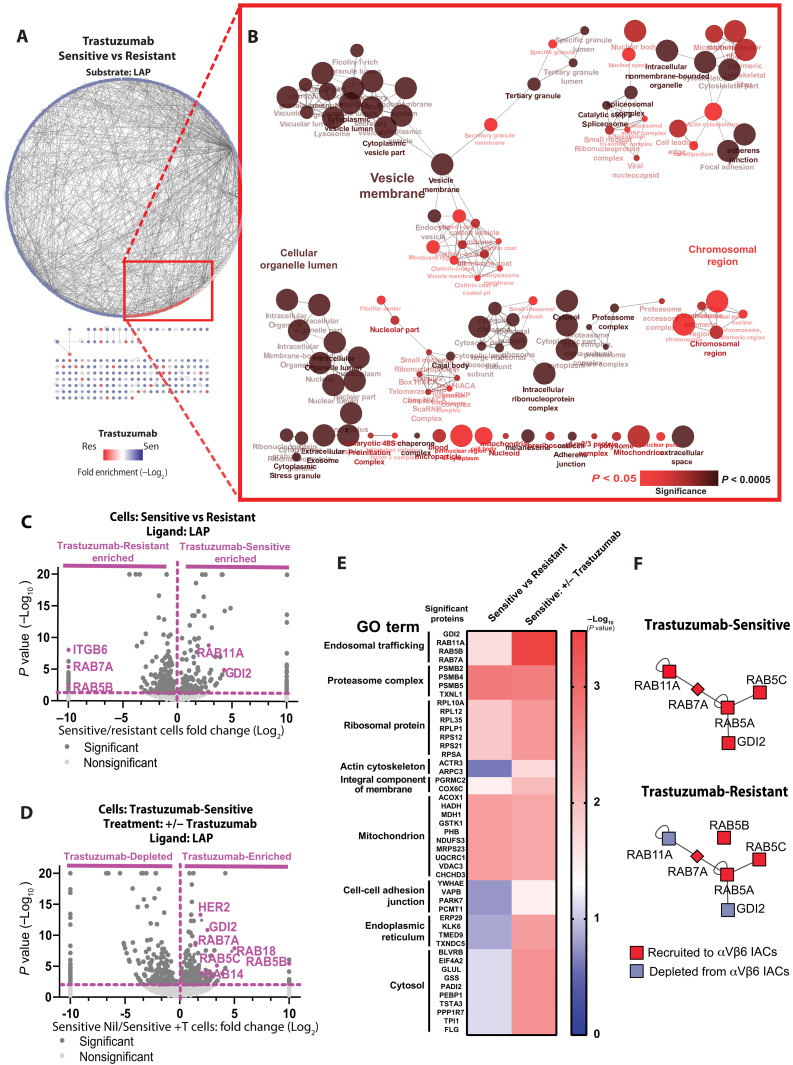
RAB5/RAB7A/GDI2 trafficking subnetwork is differentially recruited to α_V_β_6_ IACs in HER2+ breast cancer cells by trastuzumab. IAC enrichment coupled with free-label MS was used to define proteins specifically recruited to ligand-bound α_V_β_6_ in BT474 cells. (**A**) Protein-protein interaction network of proteins significantly enriched in α_V_β_6_-mediated complexes of trastuzumab-sensitive (blue nodes) and trastuzumab-resistant cells (red nodes). Lines (edges) linking nodes represent protein-protein interactions. (**B**) Visual representation of ClueGO cellular compartment GO analyses of proteins significantly enriched in α_V_β_6_-mediated complexes of trastuzumab-resistant cells in comparison with trastuzumab-sensitive cells. Node size represents the number of mapped proteins in each GO term, color indicates the level of significance of each GO term, and node clustering and edge length represent functionally grouped networks based on kappa score. See also fig. S6 (C and D) [BT474 Trastuzumab-Sensitive +/− trastuzumab (10 μg/ml)]. (**C** and **D**) Volcano plots demonstrating enrichment of proteins identified on LAP in (C) BT474 Trastuzumab-Sensitive cells (right) versus BT474 Trastuzumab-Resistant cells (left) and (D) BT474 Trastuzumab-Sensitive cells following 96 hours pretreatment with sublethal concentration of trastuzumab (10 μg/ml) or vehicle control. Statistical analysis: Fisher’s exact test; quantitative method: weighted spectra; significance level: *P* < 0.05. Significant proteins (dark gray); nonsignificant proteins (light gray); proteins of interest highlighted in purple. (**E**) Heatmap displaying statistical significance (−log_10_ *P* values) of the best hit protein per group, clustered by their main GO terms. Data obtained from analysis displayed in (C) and (D). (**F**) Schematic representation of differential enrichment of trafficking regulatory subnetwork components in trastuzumab-sensitive and trastuzumab-resistant BT474 cells. Proteins recruited to, or depleted from, α_V_β_6_ IACs are shown in red and blue, respectively. All MS data represent three independent experiments. See also fig. S6 (C and D) and data file S3.

Having identified key components of the α_V_β_6_ integrin adhesome in HER2+ breast cancer cells and established a functional link between α_V_β_6_ integrin and HER2 (Figs. 1 and 2 and fig. S4), we assessed whether exposure to trastuzumab may differentially modulate recruitment of those proteins to α_V_β_6_ IACs. The composition of isolated α_V_β_6_-mediated IACs from trastuzumab-sensitive or trastuzumab-resistant BT474 cells seeded on LAP was assessed using proteomics (Fig. 3, A to C). In parallel experiments, analysis was performed on α_V_β_6_ IACs from trastuzumab-sensitive BT474 cells, following 96 hours of treatment with a sublethal concentration of trastuzumab (10 μg/ml) or vehicle control, prior to plating on LAP (Fig. 3D and fig. S6, C and D).

Pairwise analysis was applied to proteins recruited to α_V_β_6_-dependent IACs in trastuzumab-sensitive versus trastuzumab-resistant BT474 cells (Fig. 3, A to C) and in trastuzumab-sensitive BT474 cells in the presence or absence of trastuzumab (Fig. 3D and fig. S6, C and D). Protein-protein interaction networks identified proteins differentially enriched between conditions (Fig. 3A and fig. S6C). Changes in IAC composition were analyzed to identify statistically significant changes in protein enrichment in α_V_β_6_-dependent IACs following specific treatments (Fig. 3, C and D). The primary protein cluster identified in α_V_β_6_-dependent IACs by GO term analysis, which represented terms typically associated with IAC function (Fig. 1, B and E), was not differentially recruited following trastuzumab treatment or induction of trastuzumab resistance (Fig. 3B and fig. S6D). This observation suggests that the core structural and mechanoresponsive architecture of α_V_β_6_-dependent IACs is not modulated by exposure or acquired resistance to trastuzumab. However, GO analysis revealed that terms associated with intracellular trafficking pathways (e.g., “endocytic vesicle,” “vesicle membrane,” and cytoplasmic vesicle lumen) were overrepresented in the proteins differentially recruited to α_V_β_6_-dependent IACs following acquired trastuzumab resistance (Fig. 3, A to C) or exposure to trastuzumab treatment (Fig. 3D and fig. S6, C and D). Moreover, hierarchical clustering based on analysis of functional enrichment scores revealed that the RAB5/RAB7A/GDI2 trafficking regulatory subnetwork was the highest confidence group of proteins differentially recruited to α_V_β_6_ IACs following exposure to trastuzumab (Fig. 3, E and F). By contrast, acquired trastuzumab resistance increased RAB7A and RAB5 recruitment to α_V_β_6_-dependent adhesions, but GDI2 recruitment was reduced (Fig. 3, E and F). Thus, acute exposure to trastuzumab increases recruitment of all proteins within the RAB5/RAB7A/GDI2 trafficking subnetwork, whereas acquired trastuzumab resistance decouples GDI2 from this subnetwork and suppresses GDI2 recruitment to α_V_β_6_-dependent adhesion complexes (Fig. 3F). Thus, the HER2-targeting drug trastuzumab differentially modulates recruitment of the trafficking regulatory subnetwork to α_V_β_6_ IACs.

**Fig. 4.**
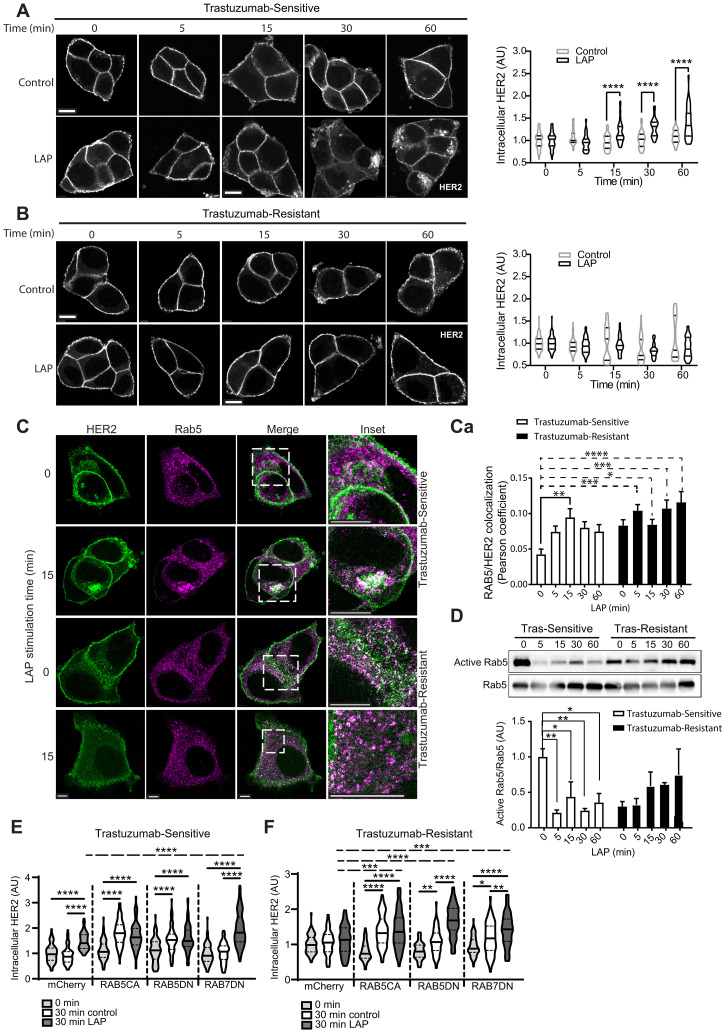
Integrin α_V_β_6_ engagement triggers internalization and vesicular accumulation of surface-labeled HER2 and modulates RAB5 activity in trastuzumab-sensitive cells. (**A** and **B**) Affibody-chase experiments. Cells surface labeled with FITC-conjugated HER2 affibody and stimulated with soluble LAP (LAP) to stimulate α_V_β_6_ integrin and trigger α_V_β_6_ endocytosis, or vehicle (Control), 0- to 60-min time course. Quantitation represents cytoplasmic HER2 fluorescence intensity analysis in (A) trastuzumab-sensitive or (B) trastuzumab-resistant BT474 cells (*N* = 3; 27 to 50 cells per condition), normalized to control trastuzumab-sensitive BT474 cells (0 min); scale bar, 10 μm. Two-way ANOVA with Šídák’s multiple comparison test. Image intensity increased in (B), relative to (A), due to low cell surface HER2 levels in trastuzumab-resistant cells to highlight internalization differences. (**C**) HER2 (green) and RAB5 (magenta) immunofluorescence in trastuzumab-sensitive and trastuzumab-resistant BT474 cells, treated with soluble LAP, 0 to 60 min (*N* = 3; 16 to 28 cells per condition); scale bar, 10 μm. (**Ca**) HER2/RAB5 colocalization quantitation (Pearson’s coefficient ± SEM). Two-way ANOVA with Dunnett’s multiple comparison test. (**D**) Active RAB5 pull-down assays. 0- to 60-min LAP stimulation time course. Quantitation of mean RAB5 activity (pull-down eluate), relative to total RAB5 (lysate) ± SEM (*N* = 3), normalized to 0-min trastuzumab-sensitive cells. One-way ANOVA with Dunnett’s multiple comparison test. (**E** and **F**) Affibody-chase experiments in (E) siControl Trastuzumab-Sensitive or (F) Trastuzumab-Resistant BT474 cells expressing constitutively active RAB5 (RAB5CA), dominant-negative RAB5 (RAB5DN), dominant-negative RAB7 (RAB7DN), or mCherry vector control. Cells were surface labeled with FITC-conjugated HER2 affibody and stimulated with soluble LAP (LAP), or vehicle control (control), for 0 or 30 min. Quantitation represents cytoplasmic HER2 fluorescence intensity (*N* = 3; 81 to 87 cells per condition); scale bar, 10 μm. One-way ANOVA with Tukey’s multiple comparison test. Representative images in fig. S10 (A and B). Further HER2 internalization analyses: Supplementary Results and fig. S11 (A to D). [(A), (B), and (D) to (F)] Data are arbitrary units (AU) normalized to control means ± SEM. [(A) to (F)] Statistical significance: *P < 0.05; **P < 0.01; ***P < 0.001; ****P < 0.0001.

The fact that trastuzumab induces α_V_β_6_ expression suggested a functional relationship integrating α_V_β_6_ and HER2 functions. Thus, we wanted to test whether direct cross-talk mechanisms exist between α_V_β_6_ and HER2. Our data demonstrated that α_V_β_6_-dependent IACs recruit HER2 and a trafficking regulatory module (Fig. 1, Ei and Fi), while trastuzumab resistance suppresses the bioavailability of HER2 at the membrane (Fig. 2, F to I) and differentially modulates recruitment of the trafficking regulatory module comprising RAB5/RAB7A/GDI2 (Fig. 3F). Therefore, we hypothesized that the putative cross-talk mechanism would involve receptor trafficking. To dissect the impact of α_V_β_6_ on HER2 trafficking, we used soluble LAP as a tool to trigger ligand-induced stimulation and endocytosis of α_V_β_6_ (*48*). In trastuzumab-sensitive BT474 cells, α_V_β_6_ stimulation triggered the endocytosis and vesicular accumulation of surface-labeled HER2 (Fig. 4A). However, despite the high levels of plasma membrane–localized α_V_β_6_, ligand-induced stimulation of α_V_β_6_ did not induce HER2 internalization in trastuzumab-resistant BT474 cells (Fig. 4B), suggesting that the endocytic mechanism integrating α_V_β_6_ integrin and HER2 is decoupled following acquired trastuzumab resistance. A similar behavior was observed in cell lines that are innately trastuzumab-sensitive or trastuzumab-resistant: LAP stimulation triggered the internalization and vesicular accumulation of surface-labeled HER2 in trastuzumab-sensitive SKBR3 and AU565 cells (fig. S7, A and B), whereas ligand engagement of α_V_β_6_ did not induce HER2 internalization in trastuzumab-resistant JIMT1 or HCC1954 cells (fig. S7, C and D), despite their elevated levels of cell surface α_V_β_6_ expression (Fig. 2H).

HER2 activation triggers autophosphorylation and intracellular signaling (*49*, *50*). Moreover, RTKs, such as HER2, are subject to precise endolysosomal trafficking mechanisms and receptor endocytosis and intracellular trafficking are essential for initiating a complete HER2 signaling response (*41*, *51*). Consistent with a mechanism integrating α_V_β_6_ and HER2 trafficking and signaling, ligand-induced stimulation of α_V_β_6_ endocytosis in trastuzumab-sensitive cells initiated differential and time-dependent regulation of HER2 expression, phosphorylation, mitogen-activated protein kinase (MAPK) and Akt activity (fig. S7, E to G). This integrin-specific stimulus also triggered a transient increase in HER2 expression, most likely via a transient inhibition of degradation (fig. S7, E to Ga and Gd). Together, these data demonstrate direct functional cross-talk between αVβ6 integrin and HER2 in trastuzumab-sensitive cells. By contrast, despite high levels of cell surface α_V_β_6_, LAP stimulation in trastuzumab-resistant cells did not initiate similar profiles of HER2 phosphorylation or signaling (fig. S7, E to G). Given the differential impact of α_V_β_6_ engagement on HER2 endocytosis and signaling in trastuzumab-sensitive and trastuzumab-resistant cells, these data suggest that α_V_β_6_ integrin–mediated regulation of HER2 is decoupled following acquired trastuzumab resistance.

### Integrin α_V_β_6_ regulates RAB5-dependent HER2 trafficking

Having identified a trafficking regulatory subnetwork specifically recruited to α_V_β_6_ IACs comprising the small GTPases RAB5 and RAB7A and the Rab regulator GDI2 (Fig. 1) and because this subnetwork is differentially recruited following treatment with, or acquired resistance to, trastuzumab (Fig. 3, C to F), we examined the role that this subnetwork plays in regulating α_V_β_6_-dependent HER2 trafficking in trastuzumab-sensitive and trastuzumab-resistant cells.

Rab GTPases coordinate intracellular trafficking mechanisms via guanosine triphosphate (GTP)–dependent recruitment of effector proteins to specific membrane compartments (*52*–*54*). Recruitment of specific Rab GTPases confers endomembrane identity, and tightly regulated coordination of GTPase activity ensures specificity and directionality of vesicular cargo transport (*52*–*54*). Precise coordination of RAB5 and RAB7A activity is essential for the maintenance and dynamics of the endolysosomal network (*55*).

To determine whether LAP-stimulated HER2 internalization involved RAB5 and RAB7A endolysosomal compartments, we assessed the subcellular distribution of HER2 following ligand-induced stimulation and endocytosis of α_V_β_6_. Chase experiments using affibody-mediated cell surface labeling of HER2, combined with immunofluorescence imaging, demonstrated that engagement of α_V_β_6_ with LAP triggered internalization of HER2 and promoted colocalization with RAB5 and α_V_β_6_ (Fig. 4, C and Ca, and fig. S8A) and with RAB7A (fig. S9, A and Ai) in trastuzumab-sensitive BT474 cells. Colocalization of integrin αVβ6 with HER2 and RAB5 following LAP-dependent stimulation of HER2 endocytosis (fig. S8A) suggests that the two receptors likely cointernalize, at least during the initial stages of endocytosis.

By contrast, trastuzumab-resistant cells exhibit a relatively high level of colocalization between HER2 and RAB5, or HER2 and RAB7A, even in unstimulated conditions (Fig. 4, C and Ca, and fig. S9, A and Aa). As LAP stimulation did not trigger HER2 endocytosis in trastuzumab-resistant cells (Fig. 4B), these data suggest that, under basal conditions, the large intracellular pool of HER2, following acquisition of trastuzumab resistance, is trapped in RAB5-positive and RAB7A-positive endosomes.

As HER2, RAB5, and RAB7A are recruited to α_V_β_6_ IACs and HER2 is trafficked to RAB5-positive and RAB7A-positive endosomes following ligand-induced stimulation of α_V_β_6_, we assessed whether LAP treatment modulates RAB5 and RAB7A activity. Effector pull-down assays showed that trastuzumab-sensitive cells exhibit a high level of steady-state RAB5 activity. However, treatment with soluble LAP induced a rapid inhibition of RAB5 activity in these cells (Fig. 4D). Trastuzumab-resistant cells had substantially lower baseline levels of RAB5 activity, compared with trastuzumab-sensitive cells. Moreover, despite the high levels of α_V_β_6_ available at the surface of trastuzumab-resistant cells, ligand-induced stimulation of α_V_β_6_ failed to suppress RAB5 activation (Fig. 4D). Thus, ligand-induced endocytosis of α_V_β_6_ drives accumulation of HER2 in RAB5-positive endosomes and suppresses RAB5 activity, in a trastuzumab sensitivity-dependent manner. Given the key role that RAB5 activity plays in the early stages of receptor internalization and endocytic trafficking (*52*, *56*), it is likely that the rapid suppression of RAB5 activity following LAP treatment (Fig. 4D) serves to limit the extent of receptor internalization.

To gain further mechanistic insight, we investigated whether RAB5 activity regulates α_V_β_6_-dependent HER2 endocytosis. As seen previously (Fig. 4A), in trastuzumab-sensitive cells, 30-min LAP stimulation induced significant HER2 internalization (Fig. 4E, mCherry) and LAP did not trigger HER2 internalization in trastuzumab-resistant cells (Fig. 4F, mCherry). However, expression of either constitutively active RAB5Q79L (RAB5CA) or dominant-negative RAB5S34N (RAB5DN) induced constitutive HER2 internalization even in the absence of LAP stimulation in both trastuzumab-sensitive and trastuzumab-resistant cells (Fig. 4, E and F, and fig. S10, A and B, RAB5CA/RAB5DN). These data suggest that RAB5 regulates internalization and intracellular trafficking of HER2 and that this likely requires precise coordination of RAB5 activity, such that perturbation of RAB5 function, either positively or negatively, dysregulates HER2 internalization. While LAP did not induce HER2 internalization in control trastuzumab-resistant cells, LAP enhanced HER2 internalization in trastuzumab-resistant cells transfected with RAB5S34N, partially mimicking trastuzumab-sensitive cells (Fig. 4, E and F, and fig. S10B, mCherry/RAB5DN). RAB5 activity plays a key role in regulating the maturation and progression of endolysosomal vesicles, which ultimately control receptor proteostasis by modulating cargo recycling or degradation, as well as endocytosis (*53*, *57*, *58*). Therefore, it is probable that the accumulation of HER2 observed in these experiments reflects both initial HER2 internalization and downstream modulation of proteostasis.

In contrast to RAB5, LAP-dependent stimulation of HER2 endocytosis was not coincident with dynamic RAB7A activity modulation in either trastuzumab-sensitive or trastuzumab-resistant cells (fig. S9B). Thus, while stimulation of α_V_β_6_ with LAP promoted colocalization of HER2 with RAB7A, it did not modulate RAB7A activity directly. However, levels of steady-state RAB7A activity were substantially higher following acquired trastuzumab resistance (fig. S9B). In addition, when assessing the effect of RAB7 activity on LAP-induced intracellular HER2 trafficking, expression of dominant-negative RAB7T22N (RAB7DN) did not affect the intracellular pool of HER2 in 30-min control-treated cells but did lead to a substantial increase in the level of intracellular HER2 following 30-min LAP treatment, relative to the mCherry control (Fig. 4E and fig. S10A, mCherry 30-min LAP versus RAB7DN 30-min LAP).

While stimulation with an αVβ6 ligand was not sufficient to modulate RAB7A activity in trastuzumab-sensitive cells (fig. S9B), dominant-negative RAB7 promoted intracellular accumulation of HER2 following ligand-dependent stimulation of αVβ6 integrin, relative to mCherry control, but did not affect intracellular HER2 accumulation in the absence of LAP stimulation (Fig. 4E, mCherry/RAB7DN). Given the role of RAB7A in Rab conversion mechanisms (*55*), the intracellular accumulation of HER2 could be due to dominant-negative RAB7 stalling the endolysosomal network and suppressing HER2 degradation.

A similar phenomenon was observed following expression of dominant-negative RAB7 in trastuzumab-resistant cells, albeit with increased intracellular HER2 in 30-min control-treated cells. While we did not study this further, it is possible that this effect in both trastuzumab-sensitive and trastuzumab-resistant cells is due to dominant-negative RAB7 suppressing lysosomal degradation of cargo following initial internalization, resulting in intracellular accumulation of HER2. Together, these analyses, and the fact that treatment with soluble LAP induced a transient increase in HER2 expression in trastuzumab-sensitive cells (fig. S7, F and G), demonstrate that both RAB5 and RAB7 control intracellular trafficking and expression of HER2 downstream of ligand-induced stimulation of α_V_β_6_. This notion is further reinforced by the fact that α_V_β_6_-dependent binding to LAP is sufficient to promote colocalization of RAB5 and RAB7A (fig. S5Ba), a key step in controlling endolysosomal transport of cargos.

Haptotactic cell migration, in which cells are guided by direct interactions of adhesion receptors with immobilized ECM ligands, requires precise coordination of integrin-mediated adhesion dynamics to control local application of mechanical force. Integrin α_V_β_6_ regulates haptotactic migration on FN (*59*, *60*), so we tested whether RAB5 activity is required for α_V_β_6_-mediated migration (fig. S10, C and D). In trastuzumab-sensitive BT474 cells, blockade of either α_V_β_6_ or HER2 inhibited cell migration. Expression of dominant-negative RAB5S34N recapitulated α_V_β_6_ and HER2 inhibition, whereas constitutively active RAB5Q79L promoted cell migration. The accelerated migration induced by constitutively active RAB5 was α_V_β_6_ independent (fig. S10C), suggesting a switch to an alternative integrin or mode of migration. Trastuzumab-resistant cells exhibited significantly higher levels of haptotactic migration relative to trastuzumab-sensitive cells. As expected, migration of trastuzumab-resistant cells was insensitive to trastuzumab-mediated inhibition of HER2. However, despite high expression of α_V_β_6_ on the cell surface (Fig. 2, C to E), the high level of migration on FN in trastuzumab-resistant cells was unaffected by α_V_β_6_ inhibition, dominant-negative RAB5, or constitutively active RAB5 (fig. S10D). Thus, the accelerated migration in trastuzumab-sensitive cells expressing constitutively active RAB5Q79L recapitulated the rapid α_V_β_6_-independent migration of trastuzumab-resistant cells.

As trastuzumab-resistant cells exhibited elevated RAB7A activity, in comparison with trastuzumab-sensitive cells (fig. S9B), we tested whether RAB7A is involved in HER2-dependent and α_V_β_6_-dependent migration. RAB7A knockdown reduced α_V_β_6_-dependent and HER2-dependent haptotactic migration on FN in trastuzumab-sensitive cells but not the high levels of α_V_β_6_-independent and HER2-independent migration of trastuzumab-resistant cells (fig. S9C). These data suggest that, despite RAB7 activation not being under direct control of α_V_β_6_/HER2 trafficking in trastuzumab-sensitive cells, RAB7 is required for α_V_β_6_-mediated and HER2-mediated migration.

As haptotactic cell migration requires dynamic and coordinated turnover of IACs to control the application of force on the ECM, these data suggest that RAB5-dependent endocytosis and trafficking of α_V_β_6_ and HER2 control adhesion dynamics to promote α_V_β_6_-dependent migration. The constitutively active RAB5Q79L construct is GTPase defective [i.e., incapable of hydrolyzing GTP to guanosine diphosphate (GDP)] and therefore accelerates endocytosis but prevents Rab conversion, further maturation of the endolysosomal network and downstream receptor recycling. The fact that constitutively active RAB5Q79L drove α_V_β_6_-independent migration in trastuzumab-sensitive cells, phenocopying trastuzumab-resistant cells (fig. S9, C and D) suggests that, (i) in trastuzumab-sensitive cells, RAB5 activity is dynamically regulated to control α_V_β_6_-dependent migration, and (ii) acquired trastuzumab resistance dysregulates the dynamic coordination of RAB5 activity. These conclusions are supported by the fact that HER2 coaccumulates with RAB5 and α_V_β_6_ in enlarged endosomes in trastuzumab-sensitive cells following LAP-triggered endocytosis, coincident with suppression of RAB5 activity, and that perturbation of RAB5 or RAB7 activity dysregulates intracellular accumulation of HER2 (Fig. 4, C to E, and figs. S8A and S10C). By contrast, trastuzumab-resistant cells exhibit higher basal levels of HER2/RAB5 colocalization, but the subcellular distribution and GTP loading of RAB5 and HER2 internalization are not affected by LAP stimulation, despite expressing high levels of α_V_β_6_ (Figs. 2, C to E, and 4, C and D).

### GDI2 regulates α_V_β_6_-dependent RAB5 activity and HER2 internalization

As RAB5 activity is dynamically regulated following LAP-mediated stimulation of α_V_β_6_ in trastuzumab-sensitive cells and regulates internalization and intracellular accumulation of HER2 and because trastuzumab-resistant cells exhibit dysregulated RAB5 activity (Fig. 4D) and receptor trafficking dynamics (Fig. 4, A to C), we next focused on mechanisms coordinating RAB5 activity.

**Fig. 5.**
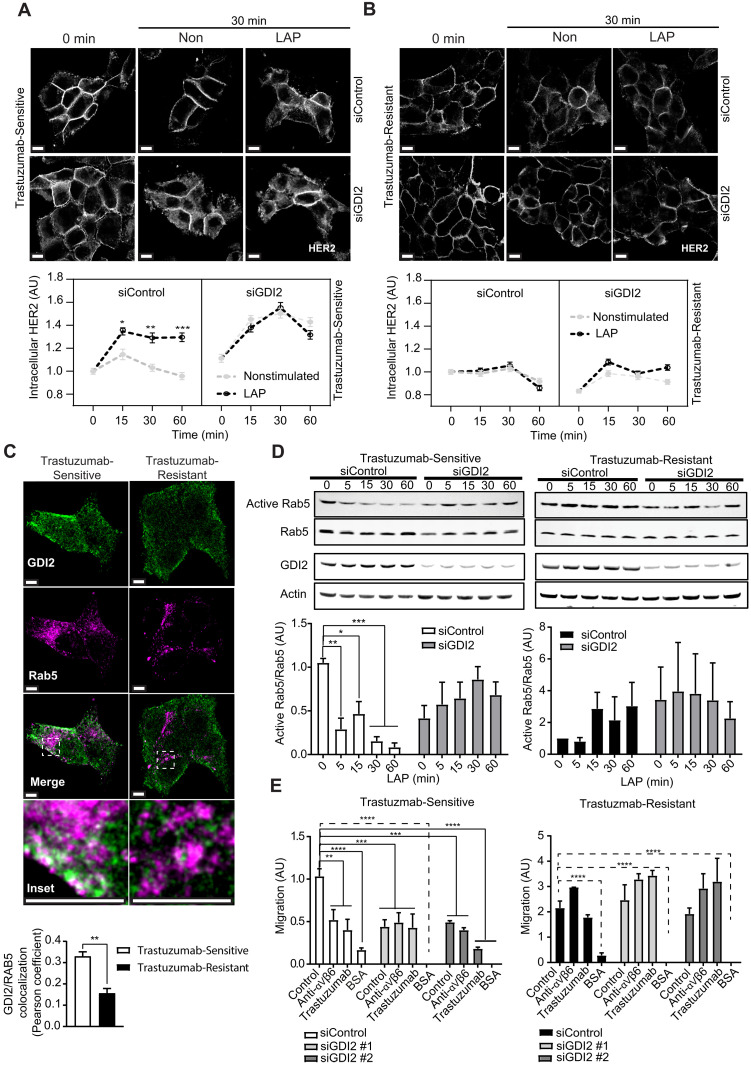
GDI2 regulates RAB5 activity and controls αVβ6-dependent HER2 endocytosis and cell migration. (**A** and **B**) Affibody-chase experiments: siControl-transfected or siGDI2-transfected BT474 cells surface labeled with FITC-conjugated HER2 affibody and stimulated with soluble LAP (LAP) to stimulate α_V_β_6_ integrin and trigger α_V_β_6_ endocytosis, or vehicle (Control), 0- to 60-min time course. Quantitation represents cytoplasmic HER2 fluorescence intensity analysis in (A) trastuzumab-sensitive or (B) trastuzumab-resistant BT474 cells (*N* = 3; 74 to 160 cells per condition); scale bars, 10 μm. Two-way ANOVA with Tukey’s multiple comparison test. Image intensity increased in (B), relative to (A), due to low cell surface HER2 levels in trastuzumab-resistant cells to highlight internalization differences. (**C**) GDI2 (green) and RAB5 (magenta) immunofluorescence in trastuzumab-sensitive and trastuzumab-resistant BT474 cells (*N* = 3; >120 cells per condition); scale bars, 5 μm. GDI2/RAB5 colocalization quantitation (Pearson’s coefficient ± SEM), two-sided *t* test. (**D**) Role of GDI2 in α_V_β_6_-dependent RAB5 activity modulation. Trastuzumab-sensitive and trastuzumab-resistant BT474 cells transfected with siRNA against GDI2 (siGDI2 #1 and #2) or control siRNA. 0- to 60-min LAP stimulation time course. Quantitation of mean RAB5 activity (pull-down eluate), relative to total RAB5 (lysate) ± SEM (*N* = 3), normalized to 0-min trastuzumab-sensitive cells. *N* = 4 independent replicate experiments. Two-way ANOVA with Šídák’s multiple comparison tests. (**E**) Haptotactic migration analysis of BT474 cells (Trastuzumab-Sensitive and Trastuzumab-Resistant) in Transwell coated with FN or BSA as a negative control. Cells were transfected with siRNA against GDI2 (siGDI2 #1 and #2) or siRNA control. Migration was assessed over 24 hours in the presence or absence of α_V_β_6_ integrin blocking antibody or trastuzumab. Data shown are means ± SEM (*N* = 3). One-way ANOVA with Šídák’s multiple comparison tests. [(A), (B), (D), and (E)] Data are arbitrary units (AU) normalized to control means ± SEM. [(A) to (E)] Statistical significance: *P < 0.05; **P < 0.01; ***P < 0.001; ****P < 0.0001.

Our MS data revealed that GDI2, a Rab-specific regulatory molecule, is recruited to α_V_β_6_ IACs in HER2+ breast cancer cells (Fig. 1). However, following acquisition of trastuzumab resistance, RAB5 and RAB7A were enriched at α_V_β_6_ adhesion sites, but GDI2 was depleted (Fig. 3F). It is thought that Rab GDIs modulate Rab function and activity by extracting inactive GDP-bound Rabs from membranes, solubilizing and chaperoning the hydrophobic prenylated GTPases in the cytosol and mediating delivery to their cognate membranes, in preparation for the next cycle of activation (*52*, *54*, *61*, *62*). The full range of GDI2 targets is unknown; however, GDI2 has been shown to associate with and modulate RAB5 (*63*–*65*). Therefore, we examined whether GDI2 regulates α_V_β_6_-dependent HER2 internalization and cell surface bioavailability. Consistent with such a role, in trastuzumab-sensitive cells, small interfering RNA (siRNA)–mediated GDI2 knockdown triggered constitutive endocytosis of HER2, even in the absence of LAP stimulation (Fig. 5A), phenocopying the enhanced HER2 internalization and accumulation induced by constitutively active and dominant-negative RAB5 in the absence of LAP treatment (Fig. 4E, RAB5CA and RAB5DN). These data suggest that, in normal cells, GDI2 serves to constrain HER2 internalization. However, GDI2 knockdown had no impact on the low level of HER2 endocytosis in trastuzumab-resistant cells (Fig. 5B), which recruit less GDI2 to α_V_β_6_-mediated IACs (Fig. 2F).

As GDI2 is a putative RAB5 regulator, we next analyzed the role GDI2 plays in RAB5 activity modulation in trastuzumab-sensitive and trastuzumab-resistant cells. Under steady-state conditions, trastuzumab-sensitive cells exhibited substantial colocalization of RAB5 and GDI2 (Fig. 5C), whereas trastuzumab-resistant cells displayed significantly less RAB5/GDI2 colocalization (Fig. 5C). Furthermore, in trastuzumab-sensitive cells, siRNA-mediated knockdown of GDI2 inhibited the α_V_β_6_-dependent suppression of RAB5 activity following LAP stimulation (Fig. 5D). GDI2 knockdown also reduced steady-state RAB5 activity in trastuzumab-sensitive cells and induced a consistent, but not statistically significant, increase in RAB5 activity following LAP stimulation (Fig. 5D), phenocopying the RAB5 activation profile observed in trastuzumab-resistant cells (Fig. 4D). Consistent with the absence of GDI2 from α_V_β_6_ IACs, in trastuzumab-resistant cells, GDI2 knockdown had no significant effect on α_V_β_6_-dependent RAB5 activity (Fig. 5D). Moreover, while GDI2 knockdown inhibited α_V_β_6_-dependent, HER2-dependent, and RAB5-dependent haptotactic migration of trastuzumab-sensitive cells, inhibiting GDI2 expression had no effect on the α_V_β_6_-independent, HER2-independent, and RAB5-independent migration of trastuzumab-resistant cells (Fig. 5E).

Together, these data show that ligand engagement of αVβ6 integrin is sufficient to trigger rapid HER2 endocytosis, which is constrained by GDI2-dependent control of RAB5 activity (Figs. 4, A to E, and 5, A to D, and fig. S7, A to D). The data suggest that GDI2 regulates α_V_β_6_-dependent RAB5 activity and RAB5-dependent HER2 internalization and cell migration in trastuzumab-sensitive cells (Fig. 5, A to E). These findings are consistent with a model whereby GDI2 is recruited to the α_V_β_6_-proximal adhesome where it modulates RAB5 activity to control α_V_β_6_ ligand–induced HER2 trafficking to coordinate α_V_β_6_-dependent migration. However, as less GDI2 is recruited to α_V_β_6_ IACs in trastuzumab-resistant cells, RAB5 activity is dysregulated, leading to perturbation of endolysosomal network dynamics and promotion of α_V_β_6_-independent cell migration. Further data and discussion relating to the functional relationship between RAB5, RAB7, and GDI2 during HER2 internalization, in both trastuzumab-sensitive and trastuzumab-resistant cells, are presented in Supplementary Results and fig. S11.

### RAB5/RAB7A/GDI2 subnetwork differentially regulates invasion and TGFβ activation in trastuzumab-sensitive and trastuzumab-resistant cells

**Fig. 6.**
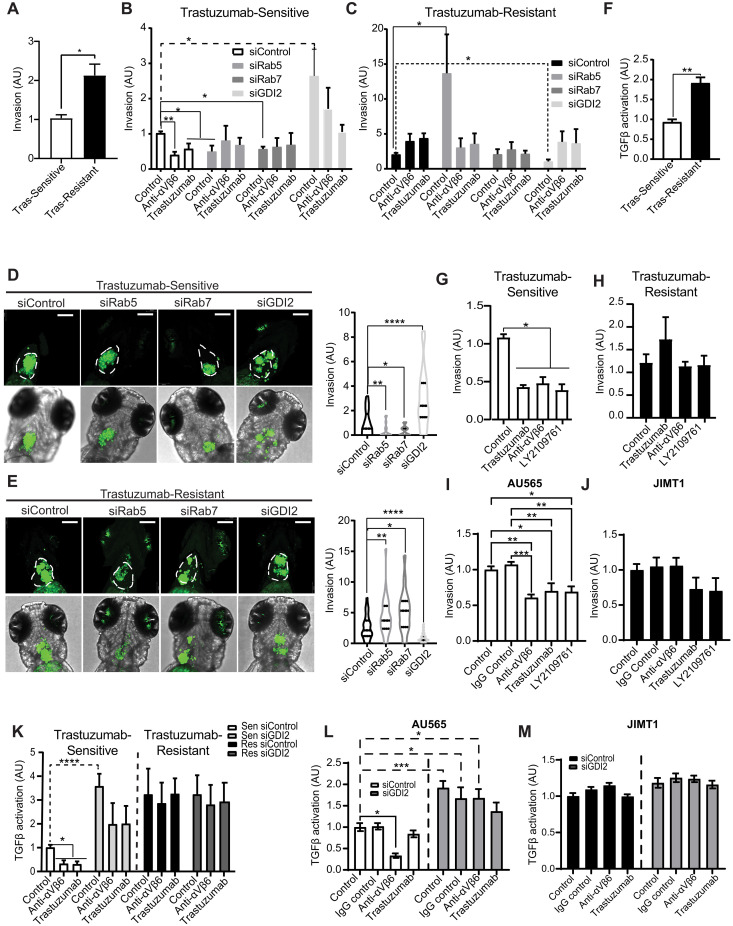
RAB5, RAB7A, and GDI2 differentially regulate invasion and TGFβ activity in trastuzumab-sensitive and trastuzumab-resistant cells. (**A**) Invasion of trastuzumab-sensitive versus trastuzumab-resistant through the cross-linked collagen-rich and FN-rich ECM (*N* = 3). Two-sided *t* test. (**B** and **C**) Invasion of siControl, siRAB5A, siRAB7A, and siGDI2 trastuzumab-sensitive (B) or trastuzumab-resistant (C) cells, in the presence or absence of integrin α_V_β_6_ blocking antibody (10 μg/ml) or trastuzumab (10 μg/ml). Note different *y* axis scales: (B) 0 to 4; (C) 0 to 20. *N* = 4. (**D** and **E**) Invasion/dissemination of CFSE-labeled siRAB5A, siRAB7A, siGDI2, or siControl BT474 trastuzumab-sensitive (D) or resistant cells (E) in a zebrafish xenograft model. Xenografts imaged 48 hours after injection. Images: maximum intensity projections; scale bars, 30 μm. *n* = 20 to 35 animals per condition. [(B) to (E)] Welch’s ANOVA with Dunnett’s multiple comparisons test. (**F**) TGFβ activity coculture assay comparing BT474 trastuzumab-sensitive and trastuzumab-resistant cells (*N* = 3). Two-sided *t* test. (**G** and **H**) Invasion of trastuzumab-sensitive (G) or trastuzumab-resistant (H) BT474 cells in the presence or absence of α_V_β_6_ integrin blocking antibody (10 μg/ml), trastuzumab (10 μg/ml), or TGFβ receptor 1/2 inhibitor (LY2109761; 10 μM) (*N* = 3). (**I** and **J**) Invasion of trastuzumab-sensitive AU565 cells (I) or trastuzumab-resistant JIMT1 cells (J) in the presence or absence of α_V_β_6_ integrin blocking antibody (10 μg/ml), trastuzumab (10 μg/ml), or TGFβ receptor 1/2 inhibitor (10 μM) (*N* = 6). (**K**) TGFβ activity analysis of siGDI2 and siControl trastuzumab-sensitive and trastuzumab-resistant BT474 cells treated with α_V_β_6_ integrin blocking antibody or trastuzumab (*N* = 4; 4 wells per biological replicate). [(G) to (K)] One-way ANOVA with Tukey’s multiple comparison tests. (**L** and **M**) TGFβ activation assays with trastuzumab-sensitive AU565 (L) and trastuzumab-resistant JIMT1 (M) cells expressing siGDI2 or siControl treated in the presence or absence of α_V_β_6_ integrin antibody (10 μg/ml) or trastuzumab (10 μg/ml) (*N* = 3; 5 wells per biological replicate). Two-way ANOVA with Šídák’s multiple comparison test. [(A) to (M)] Data are arbitrary units (AU) normalized to control means ± SEM. Statistical significance: *P < 0.05; **P < 0.01; ***P < 0.001; ****P < 0.0001.

As α_V_β_6_ is a proinvasive receptor, we next sought to determine the role of the RAB5/RAB7A/GDI2 subnetwork in invasion through FN-enriched cross-linked collagen ECM. Invasion of trastuzumab-resistant BT474 cells was significantly higher than trastuzumab-sensitive cells (Fig. 6A). Invasion of trastuzumab-sensitive BT474 cells was inhibited by a function-blocking anti-α_V_β_6_ antibody, trastuzumab, and siRNA-mediated knockdown of either RAB5 or RAB7A (Fig. 6B). These data were consistent with the effect of dominant-negative RAB5 or RAB7A-targeting siRNA in suppressing cell motility (figs. S9C and S10C). However, unexpectedly, GDI2 knockdown, which also suppressed haptotactic migration (Fig. 5E), induced a substantial increase in invasion of trastuzumab-sensitive cells (Fig. 6B and fig. S12A). Moreover, invasion of trastuzumab-sensitive cells following inhibition of GDI2 was α_V_β_6_ independent (Fig. 6B). Thus, invasion of trastuzumab-sensitive cells is driven by α_V_β_6_, HER2, RAB5, and RAB7A and suppressed by the Rab regulator GDI2.

**Fig. 7.**
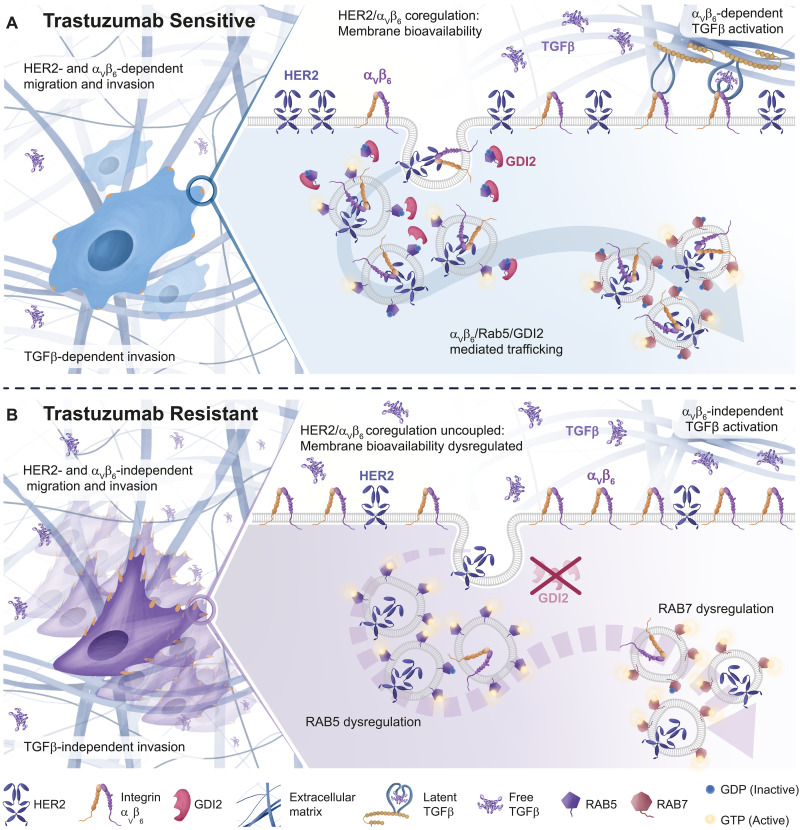
Integrin α_V_β_6_/HER2 cross-talk and trafficking drive breast cancer invasion and are dysregulated by trastuzumab resistance. (**A**) Trastuzumab-Sensitive Cells: GDI2 is recruited to sites proximal to α_V_β_6_ IACs and coordinates HER2 and α_V_β_6_ trafficking and signaling by locally modulating RAB5 activity. GDI2-mediated cross-talk between α_V_β_6_ and HER2 affects membrane availability of both receptors, ultimately influencing migration, invasion, and TGFβ activation. (**B**) Trastuzumab-Resistant Cells: GDI2 is excluded from α_V_β_6_ IACs, leading to dysregulation of RAB5 activation dynamics, followed by increased RAB7 activation. Consequently, HER2/α_V_β_6_ cross-talk is impaired, altering receptor trafficking dynamics and disrupting bioavailability of both HER2 and α_V_β_6_ integrin at the plasma membrane. This dysregulation further affects TGFβ activation, resulting in increased cell invasiveness and metastatic potential. Overall, these changes may increase the ability of cells to evade HER2 targeting drugs.

While invasion of trastuzumab-resistant cells was significantly higher than trastuzumab-sensitive cells (Fig. 6A), invasion of trastuzumab-resistant cells was α_V_β_6_ independent (Fig. 7C), despite their high level of α_V_β_6_ expression (Fig. 2, D and E). Moreover, knockdown of RAB5 induced a substantial increase in invasion of the already highly invasive trastuzumab-resistant BT474 cells (note the different scale *y* axes in Fig. 6, B and C), which was sensitive to both α_V_β_6_ inhibition and trastuzumab treatment (Fig. 6C and fig. S12A). By contrast, siRNA-mediated knockdown of GDI2 suppressed invasion of trastuzumab-resistant cells, an effect that was eliminated by α_V_β_6_ inhibition or trastuzumab (Fig. 6C). Thus, GDI2 and RAB5 differentially regulate invasion in trastuzumab-sensitive and trastuzumab-resistant cells. As GDI2 modulates RAB5 activity in trastuzumab-sensitive cells (Fig. 5D), GDI2 likely constrains invasion by controlling RAB5 activity to modulate α_V_β_6_-dependent and HER2-dependent proinvasive functions. However, this mechanism is dysregulated in trastuzumab-resistant cells, which invade in an α_V_β_6_-independent and HER2-independent manner, which is suppressed by RAB5 activity and promoted by GDI2.

Having established the impact of RAB5, RAB7A, and GDI2 on cellular invasion through FN-rich collagen matrices, we sought to analyze their role in early tumor dissemination events in vivo. Zebrafish pericardial xenografts represent a rapid and effective method to assess tumor invasion, dissemination, and tumor-stromal interactions in vivo (*66*, *67*). Trastuzumab-sensitive and trastuzumab-resistant cells, following depletion of RAB5, RAB7A, or GDI2, were injected into zebrafish embryos. While control trastuzumab-sensitive cells were able to invade into the surrounding stromal microenvironment, RAB5 and RAB7A knockdown cells showed little evidence of dissemination (Fig. 6D). By contrast, GDI2-depleted cells exhibited very high levels of invasion, relative to control cells. As observed in in vitro invasion assays, control trastuzumab-resistant cells were more invasive than their trastuzumab-sensitive counterparts in vivo. Moreover, RAB5 and RAB7A knockdown promoted invasion of trastuzumab-resistant cells, whereas GDI2 knockdown effectively eliminated invasion (Fig. 6E). These data suggest that the RAB5/RAB7A/GDI2 subnetwork, recruited to α_V_β_6_ IACs, differentially regulates invasion and dissemination of trastuzumab-sensitive and trastuzumab-resistant cells, in a physiologically relevant in vivo tissue microenvironment. Unexpectedly, despite the effect of RAB7A knockdown on invasion and dissemination of trastuzumab-resistant cells in vivo (Fig. 6C), RAB7A knockdown had no effect on invasion of trastuzumab-resistant cells in in vitro invasion assays (Fig. 6C). This may be due to the effect of RAB7A knockdown on trastuzumab-resistant cell viability in culture (fig. S12, A and B).

Cell invasion is regulated by a range of cellular functions, including haptotactic migration, matrix degradation, cell survival, and proliferation. RAB5, RAB7A, and GDI2 knockdown did not affect cell viability, a function of survival and proliferation, in trastuzumab-sensitive cells (fig. S12B) but had a substantial effect on cell invasion (Fig. 6B), whereby knockdown of RAB5 or RAB7A suppressed αVβ6-dependent invasion, yet GDI2 knockdown increased invasion substantially. These analyses demonstrated that siRNA-mediated GDI2 inhibition exerts differential effects on haptotactic cell migration on FN and invasion (Figs. 5E and 6, B and D), suppressing α_V_β_6_-dependent motility but promoting tumor cell invasion.

These data led us to investigate the mechanism underpinning this apparent discrepancy. As well as driving cell motility, α_V_β_6_ promotes tumor progression through mechanical activation of TGFβ, a potent cytokine with key roles in tumor invasion and metastasis (*68*–*72*). Consistent with their highly invasive phenotype and high levels of α_V_β_6_ expression, trastuzumab-resistant BT474 cells exhibited substantially higher levels of TGFβ activity than trastuzumab-sensitive cells (Fig. 6F). However, unexpectedly, antibody blockade of α_V_β_6_ integrin only suppressed TGFβ activity in trastuzumab-sensitive and not in trastuzumab-resistant cells (fig. S12, C and D), suggesting that other mechanisms may be contributing to TGFβ activation when cells become insensitive to trastuzumab. Consistent with this, while invasion of trastuzumab-sensitive BT474 cells was dependent on both α_V_β_6_ integrin and TGFβ receptors (Fig. 6G), invasion of trastuzumab-resistant cells was both α_V_β_6_ independent and TGFβR1/2 independent (Fig. 6H). Similarly, invasion of trastuzumab-sensitive AU565 and SKBR3 cells was driven by α_V_β_6_ integrin and TGFβ receptors (Fig. 6I and fig. S12E), whereas invasion of innately trastuzumab-resistant JIMT1 and HCC1954 cells was α_V_β_6_ independent and TGFβR1/2 independent (Fig. 6J and fig. S12F).

Together, these data suggest a degree of commonality between the mechanisms driving invasion in trastuzumab-sensitive cells and the way that these mechanisms are perturbed in trastuzumab-resistant cells, irrespective of whether the cells exhibit acquired or innate trastuzumab resistance. The data suggested that, in trastuzumab-sensitive cells, invasion is driven by modulating αVβ6-dependent TGFβ activity and cell motility. However, this mechanism is rewired in models of acquired or innate trastuzumab resistance, resulting in a different mode of invasion, which does not use αVβ6, HER2, or TGFβ.

As TGFβ activation and invasion of trastuzumab-sensitive cells were α_V_β_6_ dependent (Fig. 6, B, G, and I, and fig. S12, C and E) and components of the RAB5/RAB7A/GDI2 subnetwork differentially regulated invasion (Fig. 6, B and D), we tested the roles of RAB5, RAB7A, and GDI2 in modulating TGFβ activity in trastuzumab-sensitive and trastuzumab-resistant cells. Depletion of RAB5 or RAB7A had no effect on TGFβ activity in trastuzumab-sensitive cells, but knockdown of GDI2 induced a substantial increase in TGFβ activation (fig. S12G). By contrast, suppression of RAB5, RAB7A, or GDI2 had no effect on the already high levels of TGFβ activation in trastuzumab-resistant cells (fig. S12H). The high level of TGFβ activity in trastuzumab-sensitive BT474 and AU565 cells following GDI2 knockdown was insensitive to α_V_β_6_ integrin inhibition (Fig. 6, K and L, and fig. S12I), mimicking the phenotype of acquired and innate trastuzumab-resistant cells (Fig. 6, K and M, and fig. S12I). Thus, loss of GDI2 in trastuzumab-sensitive cells phenocopies the invasion and TGFβ activity profiles of trastuzumab-resistant cells, rendering cells highly invasive but via a mechanism that that is independent of α_V_β_6_, HER2, and TGFβ receptors.

Together, the data show that GDI2 knockdown in trastuzumab-sensitive cells dysregulates RAB5 activity (Fig. 5D) and RAB5-dependent HER2 internalization (fig. S11, A and B), enhances TGFβ activation (Fig. 6, K to M), and recapitulates the TGFβ activation profile and α_V_β_6_-independent invasion mechanisms exhibited by trastuzumab-resistant cells (Fig. 6, B, D to F, and K to M, and fig. S10, G and H). These findings suggest that GDI2 modulates RAB5 activity to coordinate HER2 trafficking, and α_V_β_6_-dependent TGFβ activation and invasion, in trastuzumab-sensitive cells. However, this mechanism is dysregulated in trastuzumab-resistant cells, which recruit less GDI2 to α_V_β_6_-mediated IACs.

Together, these findings lead to a model whereby GDI2 exerts a regulatory and suppressive effect in HER2+ breast cancer cells, limiting activation of RAB5 and constraining α_V_β_6_-dependent HER2 trafficking, signaling, and invasion (Fig. 7A). However, this mechanism becomes dysregulated in trastuzumab-resistant cells, due to loss of GDI2 from α_V_β_6_-dependent adhesion signaling complexes, enabling a high level of dysregulated invasion that is α_V_β_6_ independent, HER2 independent, and TGFβ independent (Fig. 7B).

### GDI2 and ITGB6 are potential prognostic indicators of survival and trastuzumab resistance in HER2+ breast cancer

Having identified a mechanism linking the trafficking regulators RAB5, RAB7A, and GDI2 to α_V_β_6_-mediated and HER2-mediated invasion, which is dysregulated following acquired trastuzumab resistance, we next analyzed breast cancer patient data to determine the impact of expression of RAB5, RAB7A, and GDI2 in HER2+ breast cancer.

**Fig. 8.**
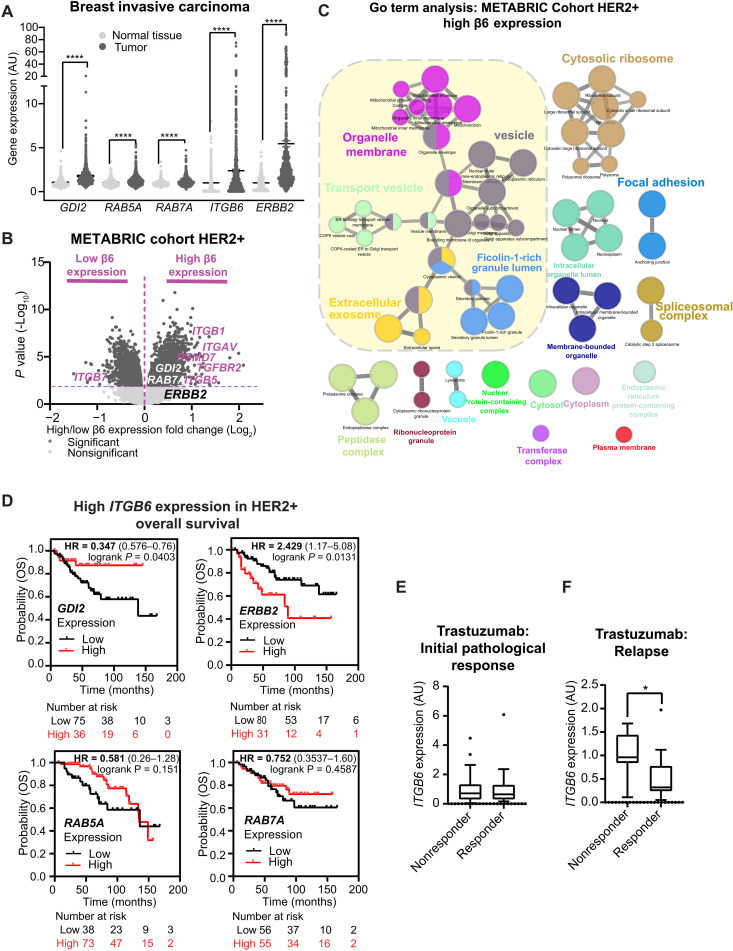
Trafficking regulatory subnetwork is highly expressed in high α_V_β_6_ expressing breast tumors and α_V_β_6_ correlates with therapeutic response. (**A**) Differential gene expression data (RNA-seq) for the *GDI2*/*RAB5A*/*RAB7A*/*ERBB2*/*ITGB6* cluster in normal breast tissue (*n* = 403; light gray) and breast invasive carcinoma (*n* = 1097; dark gray). Data were extracted from the TNMplot database (tnmplot.com). Black lines in violin blots represent the median. Mann-Whitney test. (**B**) Volcano plot showing statistical analysis (ANOVA) of RNA-seq gene expression data of patients with HER2+ breast cancer from the METABRIC cohort expressing high (Right) and low (Left) levels of *ITGB6* (Q1 versus Q4). Significant genes (dark gray); nonsignificant genes (light gray); relevant genes are highlighted in purple. (**C**) Visual representation of GO terms analysis (ClueGO, cellular compartment) of genes highly and significantly expressed in tumors expressing high levels of *ITGB6* (Q4). Colors represent specific merged GO term groups, node size represents the level of significance of each GO term, and clustering and edge length represent functionally grouped networks based on kappa score. (**D**) OS of patients with HER2+ breast cancer and with high (above median) expression of *ITGB6*, expressing high (red) or low (black) levels of *GDI2*, *ERBB2*, *RAB5A*, and *RAB7A*. (**E** and **F**) Differential *ITGB6* gene expression (gene chip) in patients with HER2+ breast cancer subdivided according to therapeutic response to trastuzumab. (E) Initial pathological complete response (responder) versus residual disease after completing therapy (nonresponder) (*n* = 77 patients). (F) RFS at 5 years (responder) versus samples relapsed before 5 years (nonresponder) (*n* = 24 patients). Two-sided Student’s *t* test. [(A), (E), and (F)] Statistical significance: *P < 0.05; ****P < 0.0001.

Initial analyses revealed that mRNA expression of *ITGB6*, *ERBB2*, *RAB5A*, *RAB7A*, and *GDI2* was all significantly higher in tumor tissue in comparison to normal tissue (Fig. 8A). Further interrogation of The Cancer Genome Atlas (TCGA) mRNA expression data across different breast cancer subtypes (Normal-like, Luminal A, Luminal B, HER2+, and Basal-like; fig. S13, A to E) demonstrated that *ITGB6* expression was higher in HER2+ breast cancer than all other subtypes (fig. S13B), and *GDI2* was increased in both Basal and HER2+, two of the most aggressive and invasive subtypes of breast cancer (fig. S13C). In addition, *RAB5A* and *RAB7A* were increased in HER2+ breast cancer in comparison to Luminal A and Normal-like subtypes (fig. S13, D and E).

As α_V_β_6_ integrin is a poor prognostic indicator (*17*, *68*, *73*–*75*), *ITGB6* gene expression is elevated in HER2+ breast cancer relative to other subtypes (fig. S13B) and α_V_β_6_ modulates HER2 trafficking and invasion (Figs. 4, A and B, and Fig. 6, B, C, G, and I, and fig. S9E), we used transcriptomic tumor gene expression data from the Molecular Taxonomy of Breast Cancer International Consortium (METABRIC) patient cohort (*76*) to identify an *ITGB6* coexpression signature in 247 patients with HER2+ breast cancer. These analyses indicated that ITGB6 gene expression positively correlated with *GDI2* (fig. S13F), *ERBB2* (fig. S13G), and *RAB5A* (fig. S13H) expression, but negatively correlated with *RAB7A* (fig. S13I) expression. To investigate these correlations further, METABRIC patients were subdivided according to expression levels of *ITGB6* [high (Q4) versus low (Q1)]. *ITGAV*, *GDI2*, *RAB7A*, and *TGFBR2* were all significantly enriched in the quartile of patients expressing highest *ITGB6* (Fig. 8B). Moreover, stratification of patients according to *GDI2* expression [high (Q4) versus low (Q1)] revealed that *ITGB6*, *RAB5A*, and *TGFBR2* expression was enriched within *GDI2* Q4 (fig. S13J). When the population was subdivided according to *RAB5A* expression (Q4 versus Q1), *GDI2* and *ITGAV* were enriched in the upper quartile patients (fig. S13K). Consistent with the mechanistic data linking these molecules functionally, these analyses demonstrate that *ITGB6*, *GDI2*, and *RAB5A* expression positively correlate in patients with HER2+ breast cancer.

GO term analysis of gene expression in high ITGB6 (Q4) expressing tumors revealed that the primary dominant cluster of genes was related to intracellular trafficking pathways (i.e., including “vesicle,” “transport vesicle,” and “extracellular exosome” GO terms among others; Fig. 8C). Critically, these data resembled our previous findings based on proteomic analysis of α_V_β_6_ IACs in HER2+ breast cancer cells (Figs. 1 and 3) and highlight the importance of the interplay between the proinvasive receptor α_V_β_6_ and trafficking pathways in patients with HER2+ breast cancer.

Having established correlations between *ITGB6*, *GDI2*, *RAB5A*, and *RAB7A* expression and considering that elevated *ITGB6* expression has been identified as an unfavorable prognostic factor among patients with HER2+ breast cancer (*17*), we explored whether coexpression of *GDI2*, *RAB5A*, or *RAB7A* with elevated *ITGB6* could further predict prognosis. Thus, we analyzed overall survival (OS) in patients expressing high levels of *ITGB6* (cutoff: median expression). Crucially, high *GDI2* expression correlated with better OS in patients with tumors expressing high levels of *ITGB6* [hazard ratio (HR) = 0.347; Fig. 8D], whereas high expression of *ERBB2* correlated with worse OS (HR = 2.429; Fig. 8D) and neither *RAB5A* nor *RAB7A* exhibited a significant correlation with OS (Fig. 8D). These data suggest that *GDI2* and *ITGB6* together may have value as prognostic indicators for OS in HER2+ breast cancer. These findings are consistent with the negative regulatory role that GDI2 plays in α_V_β_6_-dependent and HER2-dependent TGFβ activity and invasion (Fig. 6, B, D, K, and L, and fig. S12G) in trastuzumab-sensitive cells.

Our in vitro assays revealed that α_V_β_6_ integrin expression was increased in trastuzumab-resistant cells and that mechanisms governing HER2/α_V_β_6_ cross-talk and trafficking were uncoupled following acquired trastuzumab resistance. Therefore, we examined whether *ITGB6* expression might influence therapeutic efficacy of trastuzumab in HER2+ breast cancer. Patients were subdivided into two groups, responder or nonresponder, based on the clinical response of their tumors to trastuzumab neoadjuvant chemotherapy within clinically annotated datasets and the level of *ITGB6* expression determined for each patient. No significant differences in *ITGB6* expression were observed between responders and nonresponders for the initial pathological response to trastuzumab (Fig. 8E). However, *ITGB6* expression at the point of initial diagnosis was significantly higher in tumors that later relapsed within 5 years following trastuzumab treatment (Fig. 8F). Together, these results suggest that *ITGB6* levels may be a good predictor of whether trastuzumab-treated patients are likely to develop drug resistance and relapse.

Together, these data provide valuable insights into the differential expression patterns of *ITGB6*, *ERBB2*, *RAB5A*, *RAB7A*, and *GDI2* in breast invasive carcinoma. We have identified their association with specific breast cancer subtypes and demonstrated the potential prognostic significance of *ITGB6* and *GDI2* in HER2+ breast cancer. Furthermore, our findings suggest that *ITGB6* expression levels may have predictive value for long-term trastuzumab response. Thus, overall, this study highlights key molecular mechanisms driving HER2+ breast cancer progression and drug resistance (Fig. 7) that will be critical for development of prognostic and therapeutic strategies.

## DISCUSSION

HER2 and α_V_β_6_ integrin are independent predictors of breast cancer survival and metastasis (*17*). We investigated α_V_β_6_-dependent adhesion signaling complexes and identified an α_V_β_6_/HER2 cross-talk mechanism, which drives invasion and is dysregulated in trastuzumab-resistant HER2+ breast cancer cells. Collectively, the findings identify a key mechanism integrating the functions of these two proinvasive receptors and reveal that this mechanism is perturbed following trastuzumab resistance (Fig. 7). Specifically, we report that:

1) Integrin α_V_β_6_ recruits HER2 and a RAB5/RAB7A/GDI2 trafficking regulatory subnetwork and recruitment is enhanced by trastuzumab exposure but dysregulated by trastuzumab resistance.

2) Components of the trafficking regulatory subnetwork mediate direct cross-talk between α_V_β_6_ and HER2, affecting receptor trafficking and signaling.

3) Trastuzumab resistance disrupts α_V_β_6_-mediated control of HER2 endocytosis and signaling.

4) RAB5, RAB7A, and GDI2 differentially regulate invasion and TGFβ activation, but this mechanism is uncoupled by trastuzumab resistance, rendering cells unresponsive to therapeutic intervention.

5) Components of the trafficking regulatory subnetwork are highly expressed in HER2+ breast cancers expressing high levels of α_V_β_6_ and affect patient survival and α_V_β_6_ expression predicts relapse following trastuzumab treatment.

Together, these data suggest that, in trastuzumab-sensitive HER2+ breast cancer, components of the RAB5/RAB7A/GDI2 subnetwork are recruited to sites of α_V_β_6_ engagement and coordinate HER2 endocytosis, signaling, and intracellular trafficking, thus modulating HER2 bioavailability at the plasma membrane (Fig. 7A). In this instance, invasion is regulated by α_V_β_6_, HER2, and TGFβ and appears to be constrained by GDI2 (Fig. 7A). However, this mechanism is subverted in trastuzumab-resistant cells, leading to α_V_β_6_-independent and HER2-independent tumor progression (Fig. 7B), whereby GDI2 is depleted from the α_V_β_6_-proximal adhesome and invasion is unaffected by α_V_β_6_, HER2, or TGFβ inhibition (Fig. 7B). This suggests a level of cellular reprogramming that dysregulates the RAB5-dependent, RAB7-dependent, and GDI2-dependent mechanism observed in trastuzumab-sensitive cells.

On the basis of the conventional view of adhesion signaling complexes, it is perhaps unexpected that trafficking regulatory small GTPases, such as RAB5 and RAB7A, and molecules that regulate their function are recruited to α_V_β_6_-dependent IACs. However, with the advent of adhesion isolation techniques coupled with proteomic analysis and advanced imaging modalities, this view is changing (*36*, *77*). It is becoming clear that, while there is a “core” complex of structural adhesion components coupled to the actin cytoskeleton, noncanonical components can also be recruited to IACs. The emerging picture is that many noncanonical adhesome proteins are recruited to the adhesion-proximal environment. These are typically more dynamically recruited or labile and may serve regulatory roles to fine-tune adhesion function, signaling, and dynamics. Functionally and energetically, it makes sense that regulatory molecules are recruited to the adhesion-proximal environment to facilitate dynamic modulation of signaling functions. Rab GDIs regulate Rab functions and activity by extracting GDP-bound GTPases from membranes, solubilizing and chaperoning the GTPases in the cytosol and delivering them to their cognate membranes, in preparation for the next cycle of activation (*52*, *54*, *61*, *62*). In this regard, the recruitment of a GDI, such as GDI2, to the local adhesion microenvironment would enable the rapid coordination of Rab GTPase activity, extraction, and delivery to dynamically coordinate adhesion signaling and receptor trafficking.

Mounting evidence suggests that receptor endocytosis and intracellular trafficking are essential for triggering and maintaining a complete RTK signaling response (*78*–*80*). However, until recently, HER2 was thought to be retained predominantly at the plasma membrane (*78*, *81*–*88*). It is now clear that HER2 exhibits rapid trafficking kinetics, but the mechanisms coordinating these processes remain largely obscure (*41*, *89*–*91*). We demonstrate that α_V_β_6_ integrin–ligand engagement triggers HER2 endocytosis, controlling cell surface bioavailability and receptor signaling: Treatment with the α_V_β_6_ ligand, LAP, modulates HER2 phosphorylation and signaling, RAB5 activity and triggers endocytosis of HER2, which then colocalizes with RAB5-positive and RAB7A-positive endosomes. Moreover, this mechanism is perturbed by GDI2 inhibition, which induces constitutive HER2 endocytosis, consistent with a key role for GDI2 in coordinating and restricting α_V_β_6_-dependent and RAB5-dependent HER2 trafficking (*92*).

Rab GTPases recruit effector proteins to specific endomembrane compartments to precisely regulate the specificity and directionality of vesicular transport. RAB5 is a master regulator of endosome biogenesis and organization, with an essential role in assembly of the endosomal machinery (*53*, *57*, *58*). RAB5 controls vesicle formation and fusion, including homotypic fusion of early endosomes and early-to-late endosome maturation (an essential precursor to lysosomal degradation). The role of RAB7A in α_V_β_6_-triggered HER2 trafficking appears to be less direct than that of RAB5 and likely indicates a role in Rab conversion and maturation of the endolysosomal network, rather than in initial endocytosis. Early-to-late endosome maturation requires transition from RAB5 to RAB7 through the process of Rab conversion; RAB5 promotes local activation of RAB7, and when a RAB7 activity threshold is reached, RAB5 is inactivated through a negative-feedback loop (*55*, *57*, *93*, *94*). RAB7A regulates late endosome maturation, transport, clustering and fusion to lysosomes (*95*, *96*). Therefore, precise regulation of RAB5 and RAB7 activity and conversion is essential to orchestrate endolysosomal network dynamics and cargo bioavailability.

We found that invasion of trastuzumab-sensitive cells is regulated by α_V_β_6_, HER2, RAB5, and RAB7A and that activity of GDI2 constrains this invasive capacity. GDI2 is a relatively understudied Rab guanine nucleotide dissociation inhibitor, ostensibly a negative regulator of Rab function capable of extracting GDP-bound inactive Rabs from vesicular membranes and sequestering them in a cytosolic pool, before redelivery to acceptor endosomes (*65*, *97*). Our data identify GDI2 as a key regulator of α_V_β_6_-mediated RAB5 activity and HER2 endocytosis. Thus, GDI2 plays a crucial role in coordinating HER2 endolysosomal dynamics and α_V_β_6_-driven invasion. Given the roles for both RAB5 and RAB7A in this mechanism, it is tempting to speculate that GDI2 may function as a regulator of RAB5 to RAB7A conversion. This notion is supported by the fact that both RAB5 and RAB7A are required for invasion of trastuzumab-sensitive cells, whereas, following acquired trastuzumab resistance, RAB5 and RAB7A appear to have very different effects on cellular invasion. While needing further investigation, these observations may be indicative of disrupted RAB5/RAB7A conversion in trastuzumab-resistant cells, due to dysregulated GDI2 activity.

Data from the Braga lab demonstrate that PAK (p21-activated protein kinase)–mediated phosphorylation of GDI2, downstream of Rac1 activation, increases affinity of GDI2 for RAB5 (*65*), so it is conceivable that Rho family GTPase and Rab family GTPase signals converge to coordinate α_V_β_6_-dependent, RAB5-mediated, HER2 trafficking. Intriguingly, in the same study, it was demonstrated that GDI2 phosphorylation also regulates the ability of GDI2 to associate with RAB11. While RAB5 is a master regulator of endocytosis, RAB11 coordinates receptor recycling and redelivery to the plasma membrane. It is also notable that, within the trafficking regulatory subnetwork recruited to ligand-bound α_V_β_6_-dependent IACs, both GDI2 and RAB11A are depleted following acquired trastuzumab resistance (Fig. 3, C and F). While it has not been a focus of this study, it will now be important to understand how GDI2 operates to control the network of small GTPases that regulate diverse components of the endolysosomal machinery to coordinate normal cellular functions and how these processes are subverted by trastuzumab resistance. Moving forward, it will be important to investigate this intriguing hypothesis and delineate the complex role that GDI2 may play in Rab conversion, coordinating endolysosomal dynamics, and receptor recycling.

Our data provide insight into not only the underlying mechanisms coordinating HER2 endocytosis and intracellular trafficking but also how these may be bypassed upon drug resistance. Following acquired trastuzumab resistance, α_V_β_6_-dependent control of HER2 trafficking was dysregulated. This is consistent with the emergent notion that rewiring of endolysosomal and signaling networks is a key mechanism eliciting resistance to molecularly targeted therapeutics (*78*, *98*, *99*). HER2 regulates cancer proliferation, invasion, and metastasis by activation of phosphatidylinositol 3-kinase (PI3K) and MAPK signaling (*90*). In an effort to suppress HER2 oncogenic signaling, strategies have been developed to harness HER2 trafficking and promote receptor degradation. For example, HER2-, trastuzumab-, or antibody cross-linking approaches have been exploited to increase the limited level of HER2 endocytosis triggered by trastuzumab and promote lysosomal degradation (*100*–*107*). However, dysfunctional HER2 trafficking may also contribute to drug resistance mechanisms (*40*, *108*–*110*). Perturbation of HER2 endosomal trafficking machinery has been shown to modulate the response to cationic amphiphilic drugs that target the lysosome (*41*). Moreover, the reduction in HER2 cell surface expression in trastuzumab-resistant cells would likely affect the effectiveness of other HER2-targeting drugs that bind to the HER2 extracellular domain as part of their mechanism of action, either to deliver a cytotoxic payload, such as trastuzmab-DM1, or reagents that induce antibody-dependent cellular cytotoxicity.

The fact that GDI2 knockdown promotes α_V_β_6_-independent and HER2-independent invasion, in trastuzumab-sensitive cells, is consistent with promotion of a mode of invasion similar to that induced by acquired trastuzumab resistance. As GDI2 is recruited to α_V_β_6_ IACs in trastuzumab-sensitive cells, it is likely that α_V_β_6_ adhesion complexes serve as platforms to orchestrate RAB5 activity and HER2 trafficking, whereas in highly invasive trastuzumab-resistant cells, in which RAB5 activity is dysregulated, GDI2 is mislocalized, and invasion is α_V_β_6_ independent and TGFβ independent, expression of RAB5 suppresses invasion. The likely explanation for such an invasive behavior in trastuzumab-resistant cells is that dysregulated GDI2 recruitment and activity disrupts coordination of RAB5 and RAB7A activity, affecting receptor trafficking and bioavailability. In addition, as GDI2 knockdown inhibited invasion in resistant cells, these data suggest that the pool of GDI2 not recruited to α_V_β_6_ IACs may limit global RAB5 activity to enable RAB5-independent invasion.

From a therapeutic perspective, this study raises another important issue: While α_V_β_6_ targeting may be therapeutically beneficial in trastuzumab-sensitive tumors, our invasion data suggest that α_V_β_6_ inhibition may be ineffective or even counterproductive in trastuzumab-resistant breast cancer. Our in vitro data demonstrated that, despite trastuzumab-resistant cells expressing high levels of α_V_β_6_, their invasion is not responsive to α_V_β_6_ or TGFβ receptor inhibition. Moreover, analysis of patient data showed that high α_V_β_6_ expression in tumors correlates with an increased likelihood to relapse within 5 years following trastuzumab treatment. Thus, while α_V_β_6_ is clearly an appealing therapeutic target in HER2+ breast cancer, there is now a key need to assess exactly which patients might benefit from function-blocking anti-α_V_β_6_ targeting therapeutics and which may not. That said, as trastuzumab resistance increases cell surface α_V_β_6_, this study provides a strong rationale for developing α_V_β_6_ integrin as a target for delivering cytotoxic or disease modifying reagents, including targeted delivery of cytotoxic payloads or induction of antibody-dependent cellular cytotoxicity (*111*, *112*). It is also conceivable that integrin α_V_β_6_ expression could be harnessed to modify the expression of other proteins of interest, such as RTKs or immune checkpoint regulators (*113*, *114*).

If new therapeutic strategies targeting α_V_β_6_ were to be developed, it will be important to determine whether there are differences in the response to such compounds depending on whether the tumors were initially sensitive or resistant to trastuzumab. It will also be critical to explore whether the mechanism delineated in this study is disrupted in cells that exhibit innate or acquired resistance to other HER2-targeting drugs, including those that target the extracellular region of HER2 (e.g., pertuzumab) and HER2-targeting kinase inhibitors (e.g., lapatinib or neratinib).

Our data suggest that there are common features exhibited by cell lines that exhibit acquired trastuzumab resistance and cells that are inherently trastuzumab-resistant, in relation to LAP-stimulated HER2 internalization, TGFβ activation, and cell invasion characteristics. However, it should be noted that many mechanisms can drive trastuzumab resistance, including acquired mutations in PI3K and PTEN (*115*–*117*). Consistent with which, our data demonstrate elevated and dysregulated PI3K signaling in trastuzumab-resistant BT474 cells (fig. S7, F and G). Thus, it is conceivable that the mutational status of trastuzumab-resistant tumors might influence α_V_β_6_-dependent signaling networks and functions. Thus, the future development and translation of any α_V_β_6_ integrin–targeting therapeutics will need to assess whether their efficacy is affected by the mutational status of the tumor and the mechanism underpinning the resistance, rather than purely focusing on whether the tumor is trastuzumab resistant.

Given the differences identified in α_V_β_6_ IAC signaling networks, following short-term exposure to trastuzumab versus acquired resistance following extended treatment, it is conceivable that α_V_β_6_ signaling and function may differ in the context of adjuvant versus neoadjuvant trastuzumab treatment. We have not studied this, but conceivably, the duration and concentration of trastuzumab exposure, during different treatment regimes, might affect whether α_V_β_6_ can be used as a prognostic indicator or therapeutic target. Clearly, these issues demand further analysis and, going forward, it will be important to determine whether the ability to use initial α_V_β_6_ expression to predict disease relapse differs in patients given adjuvant or neoadjuvant trastuzumab. Likewise, it would be necessary to study the timing of resistance mechanisms in detail to establish when different classes of HER2-targeting and α_V_β_6_-targeting drugs might be clinically effective.

From the existing TCGA data, it is not clear what proportion of relapsed patients with HER2+ breast cancer had previously received trastuzumab. However, over the past 20 years, most patients with HER2+ breast cancer have received adjuvant or neoadjuvant trastuzumab (*47*, *118*, *119*), so it is reasonable to assume that the great majority of relapsed patients had been treated with trastuzumab, as either combination or monotherapies. Moreover, the clinical data associated with trastuzumab responder/nonresponder analyses allow confident identification of patients that were treated with trastuzumab and demonstrated that α_V_β_6_ integrin expression correlates with disease relapse, albeit based on a smaller number of patients.

Together, these analyses highlight the importance of understanding the complex interplay between the therapeutically tractable receptors α_V_β_6_ integrin and HER2 and the trafficking regulatory proteins GDI2, RAB5, and RAB7A in breast cancer to predict patient survival and design novel therapeutic strategies for HER2+ breast cancer.

Expression of α_V_β_6_ integrin is a poor prognostic indicator in HER2+ breast cancer and dual inhibition of α_V_β_6_ and HER2 in mouse xenograft models improves therapeutic effect, compared with monotherapies (*17*). However, this study presents evidence of direct α_V_β_6_-HER2 cross-talk. We used systems-level analyses to direct our functional studies and found a key mechanism integrating α_V_β_6_ integrin and HER2 functions, which drives breast cancer invasion and is dysregulated by trastuzumab resistance (Fig. 7). These findings have important clinical implications as components of the trafficking subnetwork correlate with patient survival, and αVβ6 expression may serve as a predictor for disease relapse following trastuzumab treatment. Together, the data suggest that it may be possible to identify (i) those patients who are more likely to relapse while on trastuzumab and (ii) those who may not benefit from antibody-mediated blockade of α_V_β_6_ but might be better served by α_V_β_6_-targeting therapeutics that induce cytotoxicity or modulate the immune response. It is also possible that mechanisms regulating endosomal trafficking of HER2 and α_V_β_6_ may represent novel targets for developing therapies and understanding how existing HER2-targeting therapeutics induce resistance. To apply these insights translationally, going forward, it will likely be necessary to apply machine learning approaches to gain a deeper understanding of α_V_β_6_ integrin and HER2 rewiring following acquired drug resistance and to stratify patients.

## MATERIALS AND METHODS

### Generation of trastuzumab-resistant cells

Trastuzumab (Herceptin, Roche) resuspended in sterile water to a stock concentration of 21 mg/ml was obtained from the Beatson West of Scotland Cancer Centre Pharmacy. To generate resistant cells, BT474 cells (BT474 Trastuzumab-Resistant; BT474 Tras-Res) were cultured in Dulbecco’s Modified Eagle’s medium (DMEM) supplemented with 10% fetal bovine serum (FBS), 2 mM glutamine, and penicillin (100 U/ml) and streptomycin (100 μl/ml) in the presence of increasing concentrations of trastuzumab. Trastuzumab was initially added at 10 μg/ml, increasing to 50 μg/ml after 7 days and 100 μg/ml after 3 weeks, and cells were then maintained at this concentration. At the same time, BT474 cells were simultaneously cultured in the absence of trastuzumab to generate a matched control population (BT474 Trastuzumab-Sensitive; BT474 Tras-Sen). Once the cell lines were established, resistance to trastuzumab was assessed using [3-(4,5-dimethylthiazol-2-yl)-5-(3-carboxymethoxyphenyl)-2-(4-sulfophenyl)-2H-tetrazolium (MTS)] cell viability assays (details below) in parental and trastuzumab-resistant cells.

### Cell culture

BT474 cells (wild-type and BT474 Trastuzumab-Sensitive) and HER2-18 cells were maintained in DMEM (high glucose, l-glutamine) containing 10% FBS and penicillin (100 U/ml) and streptomycin (100 μl/ml). BT474 Trastuzumab-Resistant cells were maintained in DMEM supplemented with 10% FBS, 2 mM glutamine, and penicillin (100 U/ml) and streptomycin (100 μl/ml) in the presence of trastuzumab (50 μg/ml). After resurrecting BT474 Trastuzumab-Resistant cells from frozen stocks, they were cultured in the presence of trastuzumab (200 μg/ml) for 7 days, before transferring to trastuzumab (50 μg/ml). HCC1419, SKBR3, JIMT1, HCC1954, and MDA-MB-361 cells (kindly provided by J. Ivaska, University of Turku, Finland) and AU565 cells (kindly provided by D. Yu, MD Anderson Cancer Centre, Texas, United States) were maintained in DMEM/F12 media containing 10% FBS, penicillin (100 U/ml) and streptomycin (100 μl/ml), nonessential amino acids, and 1 mM Hepes. Mink lung epithelial cells (MLECs) (kindly provided by G. Thomas, University of Southampton, United Kingdom) were maintained in DMEM (high glucose, l-glutamine) containing 10% FBS, penicillin (100 U/ml) and streptomycin (100 μl/ml), and geneticin (400 μg/ml).

### Cell viability assays

Cells were seeded on 96-well plates (1 × 10^4^ cells per well) in a complete growth medium in the absence of trastuzumab or lapatinib. After 16 hours, cells were serum starved for 4 hours and then incubated with trastuzumab or lapatinib at different concentrations (trastuzumab: 0, 25, 50, 100, and 200 μg/ml; lapatinib: 0, 0.125, 0.25, 0.5, and 1.0 μM) in serum-free media for 48 hours. Cell viability and proliferation were assessed by using an MTS assay (CellTiter 96 AQueous Non-Radioactive Cell Proliferation Assay, Promega #G5421) according to manufacturer’s instructions. Cells were incubated with the MTS reagent at 37°C and 5% CO_2_ for 1 hour prior to recording the optical density (OD) at 490 nm. Absorbance was read on a Promega GloMax microplate reader. All experimental conditions were in triplicate, and results represent three or four independent biological experiments.

### Proteomic analysis of α_V_β_6_ IACs

#### Isolation of IACs

BT474 or HER2-18 cells were seeded (5 × 10^4^ cells/cm^2^) on 10-cm plates precoated with LAP (0.5 μg/ml; Sigma-Aldrich), FN (10 μg/ml; Sigma-Aldrich), and Coll-I (10 μg/ml; Corning). After 2.5 hours of seeding, molecular complexes were stabilized by cross-linking with 3 mM dimethyl 3,3′-dithiobispropionimidate (DTBP; Thermo Fisher Scientific) at 37°C for 30 min; after two washes with cold phosphate-buffered saline (PBS) without cations, remaining DTBP was quenched with 20 mM Tris (pH 8.0). Cell bodies were removed by incubating in 20 mM NH_4_OH (Sigma-Aldrich) and 0.5% (v/v) Triton X-100 (Sigma-Aldrich) in PBS for at least 5 min prior to sonicating the samples (SONICS Vibra-Cell VCX-500 with a tapered microtip titanium alloy probe, 6.5 mm in diameter and 142 mm in length) at 20% amplitude for 1 min. Adhesion complexes were recovered from plates with 2X Laemmli buffer using cell scrapers and boiled at 95°C for 5 min and stored at −20°C for further analysis. Effective isolation of IACs was confirmed by immunoblotting.

#### Sample preparation for MS

Isolated IACs, solubilized in 2x Laemmli buffer, were resolved by SDS–polyacrylamide gel electrophoresis (PAGE) [4 to 12% Bis-Tris gels (Life Technologies) and MES-SDS buffer (Life Technologies)]. Gels were stained using the InstantBlue protein stain (Expedeon) according to manufacturer’s instructions. Stained gel sections were excised for each sample, chopped into ~1-mm^3^ pieces, and transferred to individual wells of a 96-well plate and then incubated twice with a solution containing 50% (v/v) acetonitrile (ACN) and 12.5 mM NH_4_HCO_3_ for 30 min at room temperature (RT).

Gel pieces were dehydrated by incubating twice with ACN for 5 min at RT followed by centrifugation in a vacuum concentrator (Eppendorf) at RT. To reduce proteins, gel pieces were incubated with 10 mM dithiothreitol (DTT) in 25 mM NH_4_HCO_3_ at 56°C for 1 hour. To alkylate all proteins, gel pieces were incubated with 55 mM iodoacetamide in 25 mM NH_4_HCO_3_ at 37°C for 45 min. Then, gel pieces were washed with 25 mM NH_4_HCO_3_ for 10 min at RT and dehydrated by incubating with (i) ACN for 5 min at RT and (ii) 25 mM NH_4_HCO_3_ for 5 min at RT. Steps (i) and (ii) were repeated once more. Subsequently, samples were subjected to centrifugation in a vacuum concentrator for 30 min.

Protein digestion was performed by first preincubating samples with porcine trypsin (1.25 ng/μl; Roche) for 45 min at 4°C and then incubating overnight at 37°C. Digested peptides were collected by centrifugation. Additional peptides were extracted by incubating the gel pieces in 50 μl of 99.8% (v/v) ACN/0.2% (v/v) formic acid (FA) for 30 min at RT and centrifugation, followed by incubating with 50 μl of 50% (v/v) ACN/0.1% (v/v) FA for 30 min at RT. Extracted peptides were collected by centrifugation and then pooled with the initial supernatant and evaporated to dryness in the collection plate by vacuum centrifugation. Dried peptides were resuspended in 20 μl of 5% (v/v) ACN in 0.1% FA and stored at −20°C until analysis.

#### Mass spectrometry

Four microliters of each digested fraction was injected onto a nanoACQUITY (Waters) Ultra Performance Liquid Chromatography column, coupled to an LTQ Orbitrap XL (Thermo Fisher Scientific) equipped with a nanoelectrospray source (Proxeon). Samples were resolved over a gradient using 0.1% FA (Buffer A) and 0.1% FA in ACN (Buffer B) with a flow rate of 0.300 μl/min with the following steps: 1-min wash with 1% Buffer B, increasing to 7% Buffer B over 1 min, followed by a linear gradient up to 35% Buffer B over 50 min. This was followed by a 10-min wash with 85% Buffer B and 17 min re-equilibration with 1% Buffer B. Dynamic exclusion was enabled for a repeat count of 1 for a duration of 30.00 s. MS spectra were acquired by the Orbitrap at a resolution of 30,000 and tandem MS (MS/MS) was performed on the top 12 most intense ions in the LTQ ion trap.

#### Peptide identification and proteomic analysis

Raw peptide MS data from each digested fraction were merged into a single peak list for each experimental condition and searched against a reviewed *H. sapiens* UniProt database (2018) using the Mascot Daemon (version 2.3.2) software. The initial precursor and fragment ion maximum mass deviations in the database search were set to 5 parts per million and 0.6 Da, respectively, which is optimal for linear ion trap data. One missed cleavage by the enzyme trypsin was allowed. Cysteine carbamidomethylation (C) was set as a fixed modification, whereas oxidation (M, K, and P) and phosphorylation (S, T, and Y) were considered as variable modifications.

Scaffold 4 (Proteome Software) was used to probabilistically validate protein identifications derived from MS/MS sequencing results using the X!Tandem (*120*) and ProteinProphet computer algorithms (*121*). Proteins were determined to be significantly enriched on a specific substrate or condition, based on the spectral counts of individual proteins, standardized by total spectra (quantitative value: weighted spectra), followed by pairwise Fisher’s exact tests (*P* value < 0.05, three independent experiments). To build protein-protein interaction networks, data were imported to Cytoscape 3.1 (open source, https://cytoscape.org/) and mapped using the Protein Interaction Network Analysis interactome database (*122*) (release date 21 May 2014) supplemented with a literature-curated database of IAC proteins (*37*, *38*). Significantly enriched proteins, for each experimental condition, were subjected to cellular compartment GO analysis using the Cytoscape plug-in ClueGo (*123*). To identify overlapping and hierarchical modules in protein-protein interaction networks (functional subnetworks) from proteins recruited to α_V_β_6_ IACs, the OH-PIN hierarchical clustering algorithm was used (*124*).

### Flow cytometry

To detect levels of cell surface HER2 and integrin α_V_β_6_ expression, cells were detached using trypsin (Sigma-Aldrich) and washed with a buffer containing PBS (−), 0.1% (w/v) sodium azide, and 0.1% (v/v) bovine serum albumin (BSA; wash buffer). Then, cells were incubated with a primary antibody (10 μg/ml; α_V_β_6_ integrin, Chemicon MAB2074Z) for 30 min at 4°C in a wash buffer at 4°C, washed three times, and incubated with an Alexa Fluor 647–conjugated secondary antibody and HER2 affibody fluorescein isothiocyanate (FITC) conjugated (Abcam, ab31891) at 4°C for 30 min. Cells were washed three times and then processed on an Attune NxT Flow Cytometer (Thermo Fisher Scientific) and analyzed using the FlowJo software (BD).

### Transfection

siRNA-mediated knockdown or recombinant protein expression was achieved by using the TransiT-X2 reagent (Mirus) according to manufacturer’s instructions. To achieve optimal siRNA silencing, cells were subject to two rounds of transfection with a 48-hour interval with 25 nM human RAB5A siRNA (Ambion Silencer Select Oligo #1 s11680 or Oligo #2 s11679), human RAB7A siRNA (Ambion Silencer Select Oligo #1 s15443 or Oligo #2 n289911), human GDI2 siRNA (Ambion Silencer Select Oligo #1 s5690 or Oligo #2 s5691), or AllStars Negative Control siRNA (Qiagen). Levels of protein knockdown were assessed by immunoblotting. For protein expression, cells were transfected with DNA (1 μg/ml): constitutively active RAB5 (*125*) [mcherry-RAB5CA(Q79L), Addgene plasmid #35138], dominant-negative RAB5 [mCherry-RAB5DN(S34N), Addgene plasmid #35139] (*125*), dominant-negative RAB7 [DsRed-RAB7 DN(T22N), Addgene plasmid #12662] (*126*), or empty pmCherry-C1 vector (Clontech, Addgene plasmid #3552). Transfection efficiency was confirmed by epifluorescence microscopy.

### Indirect immunofluorescence

Cells were fixed with 4% (w/v) paraformaldehyde (PFA) for 10 min and then washed once with 0.1% sodium azide in PBS to neutralize the residual PFA, followed by two washes in PBS. Cells were then permeabilized with 0.1% (v/v) Triton X-100 for 10 min, followed by blockade with 2% (w/v) BSA in PBS for 1 hour. Samples were incubated with specific primary antibody combinations in PBS containing 0.5% (w/v) BSA and 0.05% Triton X-100 for 1 hour. Primary antibodies used: RAB5 (Cell Signaling Technology, rabbit mAb #3547), RAB7 (Cell Signaling Technology, rabbit mAb #9367), HER2 (Invitrogen, mouse mAb #AHO1011), Cell Signaling Technology (rabbit mAb #2165), GDI2 (Invitrogen), and β6 Integrin (620W7, rat). Primary antibodies were detected using fluorescently conjugated secondary antibodies (Alexa Fluor 405, 488, 594, and 647; 1-hour incubation). All steps were performed at RT. Samples were mounted using the ProLong Gold Antifade Mountant (Thermo Fisher Scientific) and imaged by confocal microscopy using Zeiss LSM800 or LSM900 systems with a 63x/1.4 oil objective (voxel size: 0.09 μm by 0.09 μm by 0.2 μm). For colocalization analysis, images were first deconvolved using the Huygens professional software (Scientific Volume Imaging). Pearson’s coefficient of colocalization for the region of interest (ROI) was estimated by using the colocalization module of the IMARIS 9 software (Oxford Instruments).

### HER2 internalization assay

HER2 endocytosis was quantified in pulse-chase experiments using affibody-mediated cell surface labeling of HER2. BT474 cells were seeded at least 24 hours before the assay and then serum starved for 4 hours. Cell surface HER2 receptor was live stained with FITC-labeled anti-HER2 affibody (Abcam, ab31891) on ice for 20 min and then washed twice with ice-cold PBS. Cells were incubated with LAP (0.5 μg/ml) or control vehicle at different time intervals (0, 5, 15, 30, and 60 min) at 37°C and 5% CO_2_. Samples were fixed using 4% PFA on ice for 10 min and then washed three times with cold PBS containing 0.1% (w/v) sodium azide. Subsequently, samples were mounted using the ProLong Gold Antifade (Thermo Fisher Scientific). Samples were imaged using confocal microscopy (Zeiss LSM900) with 63x/1.4 oil immersion objective (voxel size: 0.099 μm by 0.099 μm by 0.2 μm).

Internalized HER2 receptor was analyzed by measuring fluorescence intensity in the intracellular space using FIJI. The ROI was selected by manually demarcating an area to define the intracellular space with a line to exclude the HER2 signal from the membrane for each individual cell. A total of 27 to 160 cells, per time point and experimental condition, were measured across three independent experiments (specific numbers in each figure legend).

### SDS-PAGE and immunoblotting

Cells were lysed on ice using a radioimmunoprecipitation assay buffer [150 mM NaCl, 25 mM Tris (pH 7.6), 1% (v/v) IGEPAL CA-630, 1% sodium deoxycholate, and 0.1% (w/v) SDS] supplemented with protease inhibitors [leupeptin (50 μg/μl), aprotinin (50 μg/μl), and 0.5 mM 4-(2-aminoethyl)benzenesulfonyl fluoride hydrochloride (AEBSF)] and phosphatase inhibitor cocktail (PhosStop, Roche). Lysates were sonicated (QSonica Q55-110 Q55 Sonicator, 20 kHz and 0.3 cm in diameter probe) for 10 pulses of 30 s with 50% amplitude on ice and then clarified by centrifugation (16,000*g* for 15 min), and proteins were solubilized with an SDS sample buffer and resolved by SDS-PAGE [using 4 to 12% Bis-Tris gels (Life Technologies) and MES-SDS buffer (Life Technologies)] and then transferred to a nitrocellulose membrane and blocked using 5% nonfat milk and 0.1% Tween 20 (v/v) in PBS. Primary antibodies against RAB5 (Cell Signaling Technology, rabbit mAb #3547), RAB7 (Cell Signaling Technology, rabbit mAb #9367), GDI2 (Thermo Fisher Scientific, rabbit pAb #pa5-48831), HER2 (Cell Signaling Technology, rabbit mAb #2165), pHER2 Y877 (Abcam, rabbit mAb #2241), pHER2 Y1248 (Abcam, rabbit mAb #2247), pHER2 Y1222 (Abcam, rabbit mAb #2243), pHER2 Y1196 (Abcam, rabbit mAb #6942), pHER2 Y1112 (Millipore, mouse mAb #04-294), αV-integrin (Abcam, rabbit mAb #ab179475), β6-integrin (Santa Cruz Biotechnology, goat pAb #sc-6632), β1-integrin (Abcam, rabbit mAb #ab52971), vinculin (Abcam, mouse mAb #ab11194), paxillin (BD, mouse mAb #610051), phospho-Erk 1/2 (p44/42 MAPK) T202/Y204 (pERK1/2) (Cell Signaling Technology, rabbit mAb #137F5), Akt (Cell Signaling Technology, rabbit mAb #4691), pAkt (Cell Signaling Technology, rabbit mAb #4060), GAPDH (Abcam, mouse mAb #ab9484), or β-actin (Sigma-Aldrich, mouse mAb #A3853) were incubated at 4°C overnight. Primary antibodies were detected using fluorescently or horseradish peroxidase (HRP)–conjugated secondary antibodies. Proteins were visualized using an Odyssey fluorescence detection system (LI-COR Biosciences) or a ChemiDoc chemiluminescence imaging system (Bio-Rad). Protein quantitation was performed using the “gels” function from FIJI (ImageJ) to measure band intensity (mean gray value).

### Protein purification

Expression of glutathione *S*-transferase (GST)–RAB5BD and GST-mRAB7BD recombinant proteins was induced in transformed BL21 *Escherichia coli* cells with pGEX-6P-1-hR5BD and pGEX-4 T-3-mR7BD, respectively. Cells were incubated (OD_600_ of 0.6) with 0.5 mM isopropyl-β-d-thiogalactopyranoside (Sigma-Aldrich) at 30°C for 4 hours. Pelleted bacteria were then lysed in a buffer containing 25 mM tris-HCl (pH 7.4), 1 M NaCl, 0.5 mM EDTA, 1 mM DTT, 0.1% Triton X-100, leupeptin (50 μg/μl), aprotinin (50 μg/μl), and 0.5 mM AEBSF, followed by sonication on ice. After centrifugation (10,000*g* for 10 min at 4°C), supernatant was collected and incubated with glutathione-Sepharose beads (1 ml of beads/liter of bacteria culture, Sepharose 4B, GE Healthcare) for 1 hour at 4°C by rocking. Beads were then pelleted (2000*g* for 1 min) and washed two times with a lysis buffer. Beads were washed once with a lysis buffer without Triton X-100 and then resuspended in the same buffer with 2% glycerol. Purified beads coated with recombinant proteins were aliquoted, snap frozen in nitrogen, and stored at −80°C until use. Protein purification was confirmed by SDS-PAGE followed by staining with Coomassie blue staining.

The pGEX-6P-1-hR5BD construct, comprising GST conjugated to the RAB5-binding domain of human rabaptin-5 (residues 789 to 862) (*127*), was a generous gift from V. Torres from the Faculty of Odontology of University of Chile. The pGEX-4 T-3-mR7BD construct, comprising GST conjugated to the RAB7-binding domain of murine Rab interacting lysosomal protein (*128*), was a gift from A. Edinger (Addgene plasmid #79149).

### RAB5 and RAB7A activity assays

BT474 cells were seeded at 8.9 × 10^4^ cells/cm^2^ in 10-cm dishes and incubated overnight in a full growth medium. Cells were serum starved for 4 hours prior to stimulation with LAP (0.5 μg/ml) for 0, 5, 15, 30, and 60 min. Cells were lysed in a lysis buffer containing 25 mM tris-HCl (pH 7.2), 150 mM NaCl, 5 mM MgCl_2_, 1% NP-40, 5% glycerol, and protease inhibitors by scraping. Lysates were clarified by centrifugation at 16,000*g* for 15 min. Lysate supernatants were incubated with glutathione-conjugated Sepharose beads, precoated with 30 μg of GST-RAB5BD or GST-RAB7BD, for 60 min at 4°C on a rotating shaker followed by three washes with the lysis buffer. Last, samples were boiled in 2x Laemmli buffer and analyzed by immunoblotting.

### Haptotactic migration assays

Haptotactic migration assays were performed using FN-coated polycarbonate filters (8-μm pore size, Transwell, Costar, Corning Inc.). The undersides of Transwell inserts were stained to identify cells that crossed the polycarbonate filters. Each condition was performed in duplicate in at least three independent experiments.

### Transwell invasion assays

Coll-I (2 mg/ml, 100 μl per insert) supplemented with FN (10 μg/ml; Sigma-Aldrich) solution was polymerized into 8-μm inserts (Transwell, Costar, Corning Inc.) for 1 hour at 37°C. A total of 1 × 10^5^ cells (24 hours after the second round of transfection) in 100 μl of a serum-free medium, in the presence or absence of trastuzumab (50 μg/ml; Roche), α_V_β_6_ integrin blocking antibody (10 μg/ml; 620W7), 10 μM LY2109761 (TGFβ receptor 1/2 inhibitor), or vehicle or immunoglobulin G (IgG) controls, were seeded on top of the collagen/FN gels. A medium supplemented with 10% (v/v) FBS and heregulin (20 ng/ml; Sigma-Aldrich), in the presence or absence of trastuzumab (50 μg/ml; Roche) or α_V_β_6_ integrin blocking antibody (10 μg/ml; 620W7), 10 μM LY2109761 (TGFβ receptor 1/2 inhibitor), or vehicle or IgG controls, was added to the lower wells of the plate. Cells were allowed to invade through gels for 4 days, changing media every other day. Cells were then fixed with 4% (v/v) PFA for 1 hour and permeabilized in 0.1% Triton X-100 for 1 hour at RT. Cells were stained using 4′,6-diamidino-2-phenylindole (DAPI) (Invitrogen) overnight at 4°C and then inserts were washed three times with PBS. To visualize invading cells, the underside of inserts was imaged using a Zeiss Apotome2 wide-field microscope. Invasion was quantified using the count cells plug-in from FIJI (ImageJ). All experimental conditions were in duplicate, and the average number of cells represents at least three independent experiments.

### TGFβ activation luciferase assay

To assess the capacity of cells to activate TGFβ, BT474, AU565, or JIMT1 cells were cocultured with MLECs stably transfected with an expression construct containing a truncated PAI-1 promoter fused to a firefly luciferase reporter gene (*129*). MLECs (5 × 10^5^ cells/ml) were seeded with complete media and incubated at 37°C for over 16 hours. AU565 or JIMT1 cells or Trastuzumab-Sensitive or Trastuzumab-Resistant BT474 cells were then serum starved for 4 hours and then, following preincubation with trastuzumab (50 μg/ml; Roche), α_V_β_6_ integrin blocking antibody (10 μg/ml; 620W7), 10 μM LY2109761 (TGFβ receptor 1/2 inhibitor), or vehicle or IgG controls for 10 min, were seeded (2 × 10^4^ cells per well) on top of MLECs and incubated for 16 hours. In parallel, MLECs were incubated with recombinant human TGFβ (0, 125, 250, 500, and 1000 pg/ml; PeproTech) in a serum-free medium in separate wells. Cells were then washed once with PBS and lysed with 25 μl of a reporter lysis buffer provided in the luciferase assay kit (Promega E4530) according to manufacturer’s instructions. Last, lysates were transferred to white opaque plates and 100 μl of the luciferase substrate was added. Luminescence was read on a Promega GloMax microplate reader system with pumps. All experimental conditions were in quadruplicate, and the average TGFβ activation for each assay was estimated by interpolating TGFβ concentration from luminescence values obtained with the recombinant human TGFβ standard curve. Results represent at least three independent experiments.

### Zebrafish xenograft assay

Adult zebrafish (*Danio rerio*) were maintained at the University of Manchester Biological Services Unit according to National Home Office regulations under the Animals (Scientific Procedures) Act 1986. Nacre strain (mitfa^w2a/w2a^; nacre^−/−^) zebrafish were used throughout the study to generate embryos lacking pigment, which can otherwise obscure imaging. Cells were labeled with a 5 μM CellTrace CFSE vital dye (Invitrogen) according to manufacturer’s instructions and resuspended at 1.6 × 10^7^ cells/ml on ice with PBS supplemented with a 0.5% polyvinyl pyrrolidone K60 solution (Sigma-Aldrich). Forty-eight hours after fertilization, embryos were anaesthetized with MS-222 (0.1 mg/ml; Sigma-Aldrich) and ~500 cells were injected into the pericardial cavity using a micropipette and pump (World Precision Instruments). Engrafted embryos were sorted to remove falsely injected embryos and allowed to recover at 28°C for 2 hours and then maintained at 34°C for 48 hours.

Two days after injection (dpi), engrafted zebrafish embryos were anaesthetized in MS-222 (0.1 mg/ml) and xenografts were imaged using a Leica TCS SP8 AOBS upright confocal (Leica Microsystems) with a 20x 0.50 Plan Fluotar dipping objective and 1.0x confocal zoom. Z-stacks from the top to the bottom of the tumor were acquired, and maximum intensity projections of the three-dimensional (3D) stacks are shown in the results. Captured 3D Z-stacks were processed using the Volocity Software version 6.8.9 (PerkinElmer) and the Volocity Visualization module and standard parameters. All experiments consist of at least three independent repeats. Relative invasion index is defined as the number of cells invaded outside the pericardial cavity at 2 dpi normalized to the average number of invading cells in the control group.

### Patient database analysis

To compare gene expression between normal (normal samples from noncancerous patients and further pediatric tissues) and tumor tissue, we used the online resource tnmplot.com and analyzed RNA sequencing (RNA-seq) data for breast invasive carcinoma (*130*). To assess gene expression in different subtypes of breast cancer, RNA-seq gene expression data from the Breast Invasive Carcinoma TCGA, PanCancer Atlas dataset (*131*) were extracted using the online resource https://cbioportal.org (accessed date: 29 February 2023). Patients were subdivided into Luminal A, Luminal B, HER2+, Basal-like, and Normal-like categories. Following a Kruskal-Wallis test, the statistical significance threshold was set at *P* < 0.05.

To determine gene expression in patients with HER2+ breast cancer, RNA-seq gene expression data from the METABRIC dataset (*76*, *132*, *133*) were extracted using cbioportal.org. In this case, patient data were split according to *ITGB6* gene expression levels in quartiles, followed by gene enrichment analysis (differential expression) comparing the upper quartile (Q4, high *ITGB6* expression) and the lower quartile (Q1, low *ITGB6* expression), using DESeq2 (*134*) in the R software (RStudio, Spotted Wakerobin). Significance was set at *P* < 0.01, and data were visualized by volcano plot. Significantly enriched genes in Q4 were the subject of cellular compartment GO term analysis using the Cytoscape plug-in ClueGO.

Kaplan-Meier analysis of OS was generated using the online resource kmplot.com/analysis and using mRNA gene chip data to determine gene expression in patients with breast cancer (*135*). Patient data were split using the autoselect best cutoff tool, using the “all probe sets per gene” option, and restricting searches to HER2+ subtype based on array data. To analyze the impact of expression levels of *gene1* in subpopulations of high *gene2* expression (i.e., *ITGB6*), data were filtered by median expression of *gene2* using the “use multiple genes” tool, generating two subpopulations including: (i) only patients expressing high levels of *gene2* and (ii) only patients expressing low levels of *gene2*. The HR was estimated from each individual Kaplan-Meier analysis and summarized in heatmaps and supplementary tables.

Correlations between *ITGB6* gene expression with initial pathological complete response or relapse-free survival (RFS) after 5 years, following trastuzumab treatment, from breast cancer patient data, were generated using the online resource https://rocplot.org. Patients with HER2+ breast cancer treated with trastuzumab were assigned to two cohorts (responder and nonresponder) based on their clinical characteristics (*136*). Data are based on all probes available for *ITGB6*. Data were extracted and analyzed using GraphPad Prism version 8. The total number of patients for initial pathological complete response was 57 (34 responders and 23 nonresponders) and, for RFS, was 44 (26 responders and 18 nonresponders). Both cohorts were compared using unpaired *t* tests.

### Statistical analysis

To assess significance, statistical analysis was performed using appropriate methods based on the data distribution. For normally distributed data, unpaired, two-tailed Student’s *t* test with unequal variance or analysis of variance (ANOVA) were used, followed by a specific post hoc multiple test correction, according to the statistical hypothesis being tested: Tukey’s multiple comparison test was used when comparing the difference between each pair of means, Dunnett’s method was used to compare each mean with a control mean, and Šídák’s correction was used when comparing a specific set of means. In cases where the data did not follow a normal distribution, nonparametric Mann-Whitney *U* test or Kruskal-Wallis *H* test was used, depending on the number of groups being analyzed, followed by Dunn’s or Welch’s multiple comparison tests, depending on assumed equal or unequal variance.

The number of samples, experiments, or replicates for each figure is stated in the relevant figure legends. *P* values are denoted by asterisks: (*) for *P* ≤ 0.05, (**) for *P* ≤ 0.01, (***) for *P* ≤ 0.001, and (****) for *P* ≤ 0.0001.

Data presentation includes bar charts with SEM (± SEM) or kernel density violin plots illustrating the frequency distribution of the data, with dashed lines representing the median and dotted lines indicating the quartiles. GraphPad Prism versions 8, 9, and 10 (GraphPad Software Inc.) were used for statistical analyses, except for MS analysis (Scaffold 4, Proteome Software) and GO term/networking analysis (Cytoscape 3).

## Supplementary Materials

### The PDF file includes:

Supplementary Results and Discussion Figs. S1 to S13 Legends for supplementary files S1 to S3
