# Supplementary material for: A trafficking regulatory subnetwork governs αVβ6 integrin-HER2 cross-talk to control breast cancer invasion and drug resistance
Source: Sci Adv. 2024 Dec 4;10(49):eadk9944. doi: 10.1126/sciadv.adk9944 (PMC11616693; doi:10.1126/sciadv.adk9944)
Supplement: Supplementary file 1 — Supplementary Results and Discussion Figs. S1 to S13 Legends for supplementary files S1 to S3 [file sciadv.adk9944_sm.pdf]

Science Advances
AAAS

Supplementary Materials for

A trafficking regulatory subnetwork governs $\alpha_V\beta_6$ integrin-HER2 cross-talk to control breast cancer invasion and drug resistance

Horacio Maldonado *et al.*

Corresponding author: Mark R. Morgan, mark.morgan@liverpool.ac.uk

Sci. Adv. **10**, eadk9944 (2024)
DOI: 10.1126/sciadv.adk9944

The PDF file includes:

Supplementary Results and Discussion
Figs. S1 to S13
Legends for supplementary files S1 to S3

Other Supplementary Material for this manuscript includes the following:

Supplementary files S1 to S3

SUPPLEMENTARY RESULTS & DISCUSSION

Overview

Data presented in the main **Results** section indicate that key components of the trafficking regulatory subnetwork recruited to $\alpha_V\beta_6$ adhesion complexes, regulate HER2 endocytosis and trafficking; suggesting a role for these molecules in mediating integrin $\alpha_V\beta_6$-HER2 crosstalk, TGFβ activation and invasion of HER2+ breast cancer cells. Moreover, these mechanisms are dysregulated in trastuzumab-resistant cells.

In this **Supplementary Results** section, we present further data that highlights the complex interplay between RAB5, RAB7A and GDI2, in the context of $\alpha_V\beta_6$-dependent HER2 trafficking. We explore the relationship between components of the trafficking regulatory sub-network, identify features modulating $\alpha_V\beta_6$/LAP-dependent HER2 internalisation and intracellular accumulation, and speculate on potential mechanisms modulating $\alpha_V\beta_6$ and HER2 proteostasis, signalling and function, that demand further investigation in the future.

Interplay of RAB5, RAB7A and GDI2 in HER2 in LAP-dependent HER2 internalisation and accumulation

NB: Data from Fig. 4E/F are re-presented in the Supplementary Materials as Fig. S11A and S11C, respectively, alongside further conditions modulating GDI2 expression in conjunction with manipulation of RAB5 and RAB7 activity.

As highlighted in the main **Results** section, in siControl trastuzumab-sensitive cells, 30 min LAP stimulation induced significant HER2 internalisation (Fig. 4E/S11A; mCherry). Whereas expression of either constitutively active RAB5Q79L (RAB5CA) or dominant negative RAB5S34N (RAB5DN) induced constitutive HER2 internalisation and intracellular accumulation, even in the absence of LAP stimulation (Fig. 4E/S11A, RAB5CA/RAB5DN). Superficially, these characteristics seem to mimic the effect of suppressing GDI2 expression on HER2 internalisation, which leads to enhanced HER2 internalisation, even in the absence of LAP stimulation (Fig. 5A).

However, following GDI2 knockdown, constitutively active RAB5Q79L (RAB5CA) renders cells sensitive to LAP stimulation, further increasing HER2 internalisation relative to untreated (30 min Control) cells (Fig. S11A/B, RAB5CA). Moreover, expression of dominant negative RAB5S34N (RAB5DN) in GDI2 knockdown cells, reduces levels of HER2 internalisation relative to untreated cells (Fig. S11A/B, RAB5DN). Importantly, these data suggest that in wildtype cells, GDI2 constrains RAB5-driven LAP-induced internalisation. Such that in the absence of GDI2 expression, elevated Rab5 activity can further promote LAP-induced HER2 intracellular accumulation. Whereas reduced RAB5 functionality, as a result of RAB5DN, reduces the level of HER2 internalisation following LAP treatment.

Together, these data suggest that in control cells, GDI2 serves to limit the activity of RAB5; perhaps acting as a break to constrain dysregulated receptor trafficking. Given the recruitment of GDI2 to HER2-enriched $\alpha_V\beta_6$-mediated adhesion complexes, it is tempting to speculate that GDI2 could serve a key role in co-ordinating spatiotemporal regulation of trafficking dynamics

and signalling. Whereas, in the absence of GDI2, as seen in trastuzumab-resistant cells which exhibit reduced levels of GDI2 proximal to $\alpha_V\beta_6$ IACs, these control mechanisms appear to be dysregulated.

Data presented in the **Results** section, indicated that dominant-negative RAB7T22N (RAB7DN) led to increased intracellular HER2 levels following 30 min LAP treatment, relative to nil treatment and mCherry control cells (Fig. 4E, S11A; mCherry 30 min LAP vs RAB7DN 30 min LAP, RAB7DN 30 min nil vs RAB7DN 30 min LAP). Loss of GDI2 resulted in elevated intracellular accumulation of HER2 in RAB7DN-expressing cells, but to no greater extent than in mCherry control siGDI2 cells (Fig. S11B; mCherry 30 min LAP vs RAB7DN 30 min LAP). These data, and the fact that RAB7 typically operates downstream of RAB5 in the endolysosomal pathway (*53, 55, 57, 58*), may suggest that any potential effect of GDI2 on RAB7-mediated functions, is likely due to effects on upstream trafficking regulators such as RAB5, rather than directly on RAB7A. The enhanced response to LAP in siControl cells expressing dominant-negative RAB7, relative to mCherry, in the presence of LAP treatment, could also be consistent with the effect of GDI2 knockdown on earlier steps in the trafficking pathway, such as modulation of RAB5 activity. This would also be in agreement with data showing that, while trastuzumab-resistant cells exhibited high levels of RAB7A activity, LAP stimulation and the associated induction of HER2 endocytosis did not modulate RAB7A activity in either trastuzumab-sensitive or trastuzumab-resistant cells (Fig. S9B). So, while stimulation of $\alpha_V\beta_6$ with LAP promotes co-localisation of HER2 with RAB7A, it does not modulate RAB7A activity directly.

While these studies suggest a complex relationship exists between GDI2, RAB5 and RAB7A, it is important not to over-interpret the data. The experimental setup does not enable us to distinguish between changes in initial HER2 endocytic rates, versus downstream accumulation of HER2 due to inhibition of HER2 degradation. While it is possible to speculate on these characteristics, based on what is known about the role of different GTPases in modulating endolysosomal biogenesis, transport and degradation, the complexity of the system means these conclusions should be treated cautiously. To get genuine mechanistic and spatiotempotral insight, going forward, it will be essential to employ super-resolution live cell imaging approaches to determine where and when the specific GTPases are recruited to different endomembranes, following specific and pulsed stimuli.

Despite the lack of evidence that GDI2 modulates RAB7 activity directly, it is likely that RAB7 still plays a critical role in controlling $\alpha_V\beta_6$ and HER2 functions. Integrin and RTK function and expression are under direct control of the endolysosomal network, which exerts spatiotemporal control over intracellular trafficking, recycling and degradation mechanisms *(28, 41, 77)*. RAB5 to RAB7 conversion is a key step within this pathway and the prevailing model suggests that a cut-off switch exists, which requires localised activation of RAB7 to ensure directionality and irreversibility of late endosome biogenesis (*93)*. Thus, there is now a need to fully explore how GDI2 and other GTPase regulators, modulate RAB conversion and the biogenesis of the endolysosomal network, to co-ordinate $\alpha_V\beta_6$ and HER2 bioavailability and function.

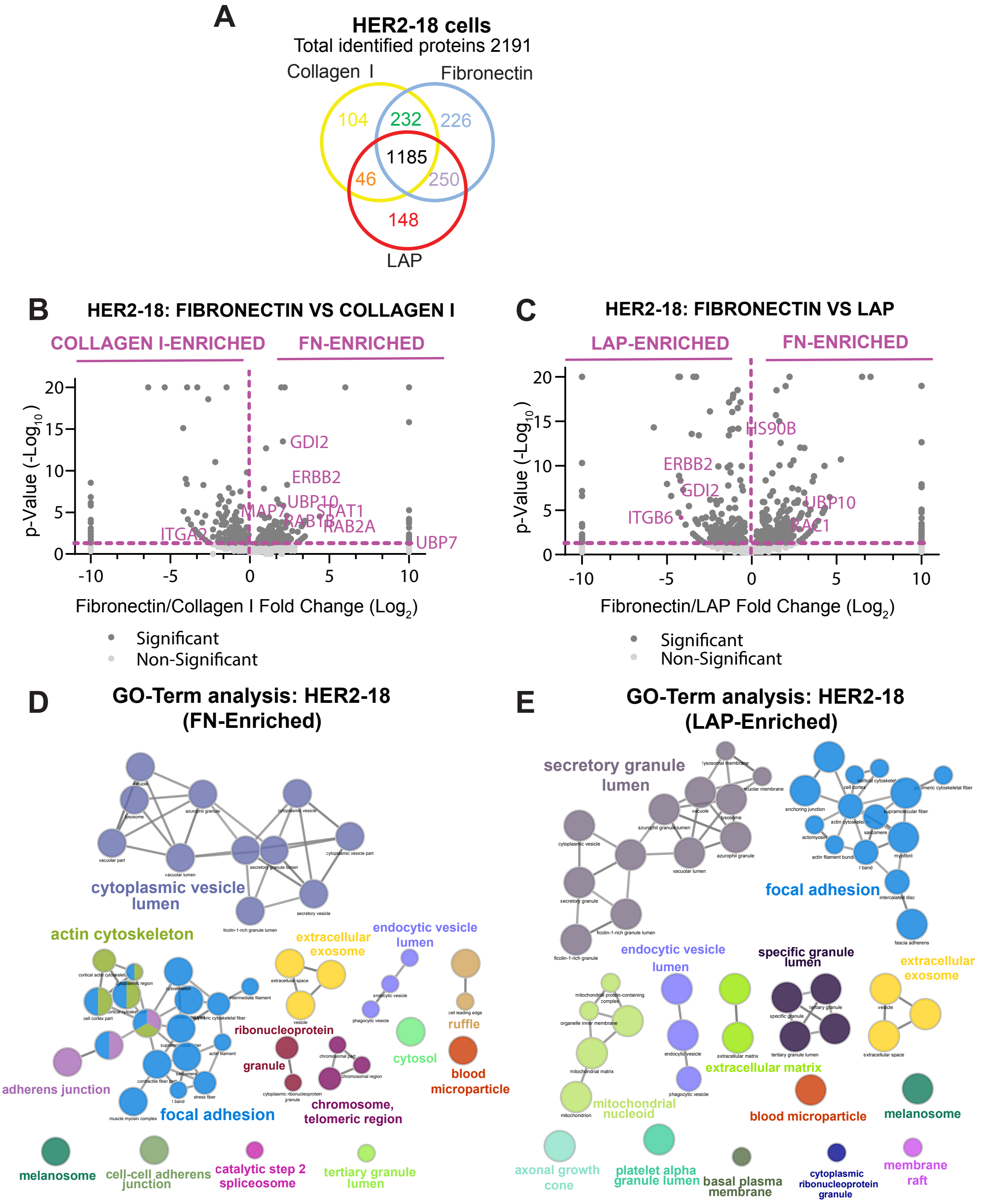
A
HER2-18 cells
Total identified proteins 2191
Collagen I
Fibronectin
LAP
104
232
226
1185
46
250
148
B
HER2-18: FIBRONECTIN VS COLLAGEN I
COLLAGEN I-ENRICHED
FN-ENRICHED
p-Value (-Log10)
Fibronectin/Collagen I Fold Change (Log2)
GDI2
ERBB2
UBP10
MAP7
STAT1
RAB1B
RAB2A
ITGA2
UBP7
Significant
Non-Significant
C
HER2-18: FIBRONECTIN VS LAP
LAP-ENRICHED
FN-ENRICHED
p-Value (-Log10)
Fibronectin/LAP Fold Change (Log2)
HS90B
ERBB2
GDI2
ITGB6
UBP10
RAC1
Significant
Non-Significant
D
GO-Term analysis: HER2-18 (FN-Enriched)
cytoplasmic vesicle lumen
actin cytoskeleton
extracellular exosome
endocytic vesicle lumen
ruffle
ribonucleoprotein granule
cytosol
blood microparticle
adherens junction
focal adhesion
chromosome, telomeric region
melanosome
cell-cell adherens junction
catalytic step 2 spliceosome
tertiary granule lumen
E
GO-Term analysis: HER2-18 (LAP-Enriched)
secretory granule lumen
focal adhesion
endocytic vesicle lumen
specific granule lumen
extracellular exosome
extracellular matrix
mitochondrial nucleoid
blood microparticle
melanosome
axonal growth cone
platelet alpha granule lumen
basal plasma membrane
cytoplasmic ribonucleoprotein granule
membrane raft

FIGURE S1

Figure S1: Label-free proteomic analysis of IAC enrichment in HER2-18 cells seeded on different matrices. IAC enrichment coupled with free-label mass spectrometry analysis was used to define proteins specifically recruited to ligand-bound $\alpha_V\beta_6$ in HER2-18 cells. **A)** Venn diagram showing total identified proteins recruited to IAC on different substrates including LAP ($\alpha_V\beta_6$-selective ligand), fibronectin ($\alpha_V\beta_6$-binding non-selective ligand) and collagen-I (non-$\alpha_V\beta_6$-binding ligand). 2,191 proteins were identified with at least 90% confidence. Overlap of proteins identified on all ligands: 1,185 proteins; 54%. Detected proteins unique to each ligand – LAP: 148; fibronectin: 226; collagen-I: 104; or shared between two ligands - LAP/FN: 250; LAP/Collagen-I: 46; FN/Collagen-I: 232. **B/C)** Volcano plots demonstrating enrichment of proteins identified on **(B)** fibronectin (right) and collagen-I (left) matrices and **(C)** fibronectin (right) and LAP (left) matrices. Pairwise statistical analysis: Fisher's exact test; quantitative method: weighted spectra; significance level: $P < 0.05$. Significant proteins (dark grey); non-significant proteins (light grey); proteins of interest highlighted in purple. **D/E)** Visual representation of ClueGO cellular compartment gene ontology (GO) analyses of proteins significantly enriched on **(D)** Fibronectin in comparison with Collagen-I, or **(E)** LAP in comparison with Fibronectin in HER2-18 cells. Colours represent specific merged GO term groups, node size represents level of significance of each GO term, clustering and edge length represent functionally grouped networks based on kappa score. All mass spectrometry data represent 3 independent experiments.

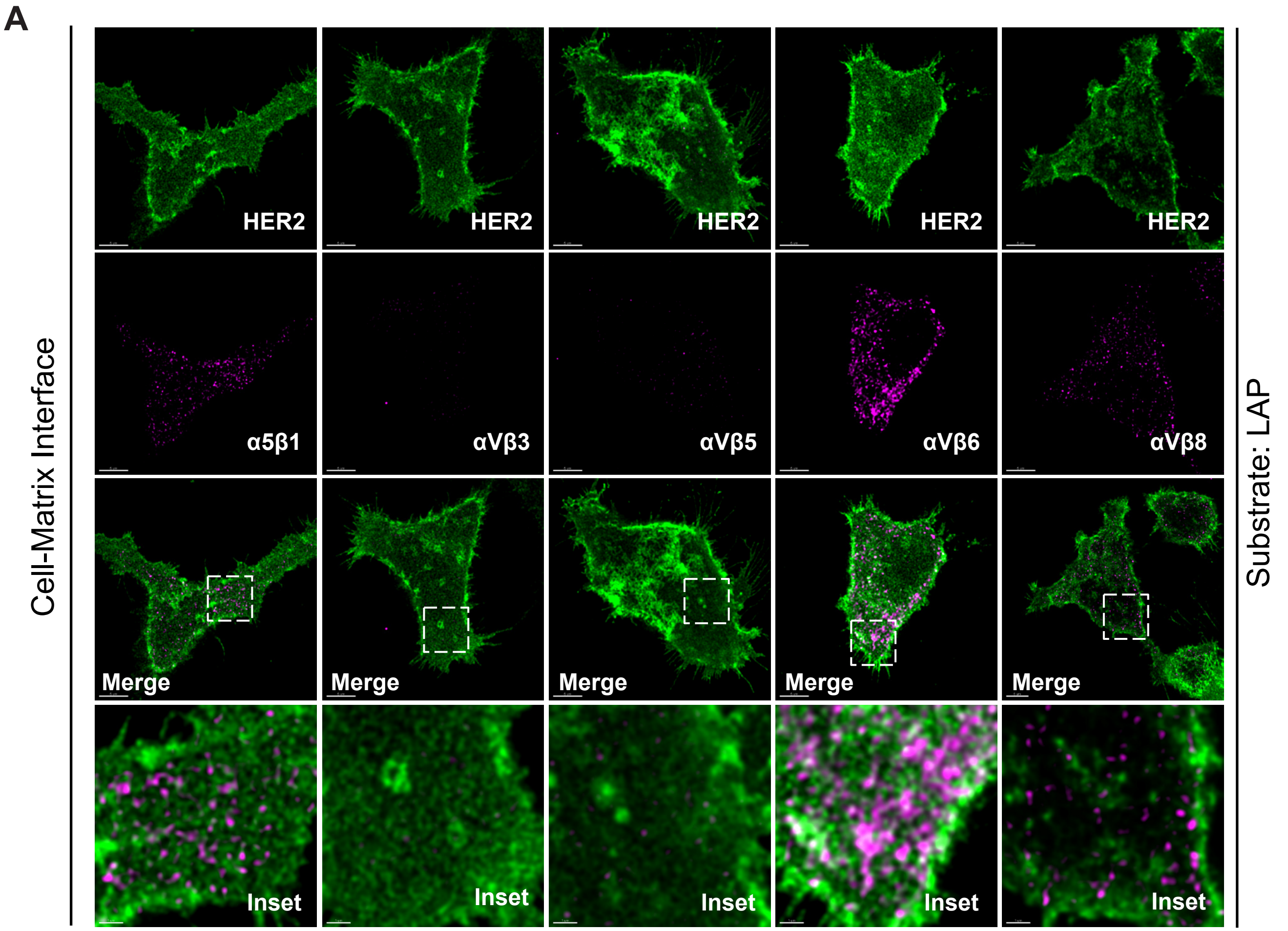
A
Cell-Matrix Interface
Substrate: LAP
HER2
α5β1
αVβ3
αVβ5
αVβ6
αVβ8
Merge
Inset

FIGURE S2

Figure S2: Integrin $\alpha_V\beta_6$ is the primary RGD-binding integrin recruited on LAP and co-localises with HER2 at the cell-matrix interface. Immunofluorescent staining of RGD-binding integrins $\alpha_5\beta_1$, $\alpha_V\beta_3$, $\alpha_V\beta_5$, $\alpha_V\beta_6$ & $\alpha_V\beta_8$, (magenta) and HER2 (green) in HER2-18 cells attached to LAP. Images show the basal Z-plane representing the cell-matrix interface. White pixels indicate integrin/HER2 colocalisation. Dashed boxes indicate location of insets; scale bar represents 10 µm.

FIGURE S3

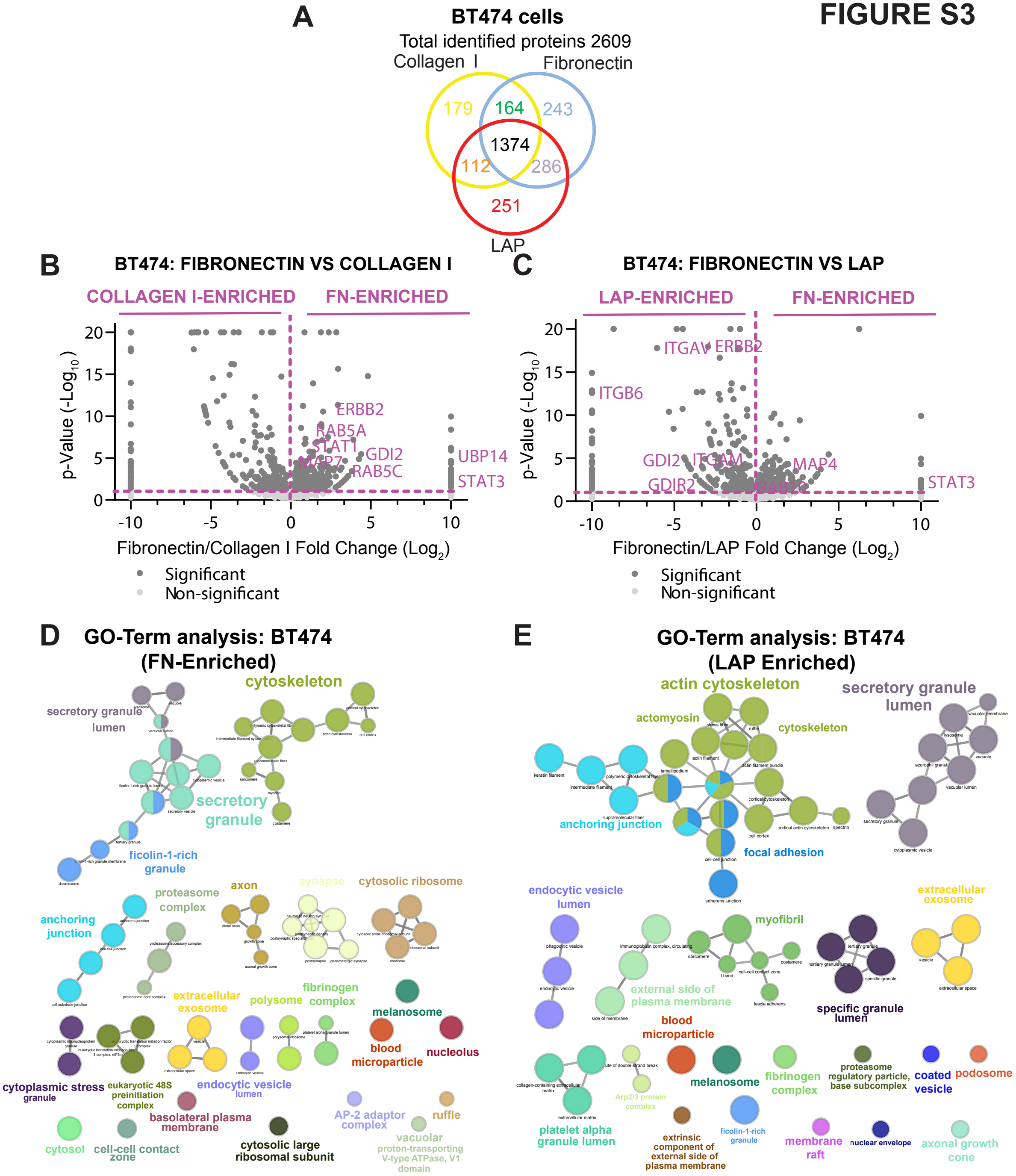
A
BT474 cells
Total identified proteins 2609
Collagen I
Fibronectin
LAP
179
164
243
1374
112
286
251
B
BT474: FIBRONECTIN VS COLLAGEN I
COLLAGEN I-ENRICHED
FN-ENRICHED
p-Value (-Log10)
Fibronectin/Collagen I Fold Change (Log2)
ERBB2
RAB5A
STAT1
GDI2
MAP7
RAB5C
UBP14
STAT3
Significant
Non-significant
C
BT474: FIBRONECTIN VS LAP
LAP-ENRICHED
FN-ENRICHED
p-Value (-Log10)
Fibronectin/LAP Fold Change (Log2)
ITGAV
ERBB2
ITGB6
GDI2
ITGAM
MAP4
GDIR2
RAB10
STAT3
Significant
Non-significant
D
GO-Term analysis: BT474 (FN-Enriched)
cytoskeleton
secretory granule lumen
secretory granule
ficolin-1-rich granule
proteasome complex
axon
synapse
cytosolic ribosome
anchoring junction
extracellular exosome
polysome
fibrinogen complex
melanosome
blood microparticle
nucleolus
cytoplasmic stress granule
eukaryotic 48S preinitiation complex
endocytic vesicle lumen
basolateral plasma membrane
AP-2 adaptor complex
ruffle
vacuolar proton-transporting V-type ATPase, V1 domain
cytosol
cell-cell contact zone
cytosolic large ribosomal subunit
E
GO-Term analysis: BT474 (LAP Enriched)
actin cytoskeleton
secretory granule lumen
actomyosin
cytoskeleton
anchoring junction
focal adhesion
endocytic vesicle lumen
extracellular exosome
myofibril
external side of plasma membrane
specific granule lumen
blood microparticle
melanosome
fibrinogen complex
proteasome regulatory particle, base subcomplex
coated vesicle
podosome
platelet alpha granule lumen
Arp2/3 protein complex
extrinsic component of external side of plasma membrane
ficolin-1-rich granule
membrane raft
nuclear envelope
axonal growth cone

Figure S3: Label-free proteomic analysis of IAC enrichment in BT474 cells seeded on different matrices. IAC enrichment coupled with free-label mass spectrometry analysis was used to define proteins specifically recruited to ligand-bound $\alpha_V\beta_6$ in BT474 cells. **A)** Venn diagram showing total identified proteins recruited to IAC on different substrates including LAP ($\alpha_V\beta_6$-selective ligand), fibronectin ($\alpha_V\beta_6$-binding non-selective ligand) and collagen-I (non-$\alpha_V\beta_6$-binding ligand). 2,609 proteins were identified with at least 90% confidence. Overlap of proteins identified on all ligands: 1,374 proteins; 53%. Detected proteins unique to each ligand – LAP: 251; fibronectin: 243; collagen-I: 179; or shared between two ligands - LAP/FN: 286; LAP/Collagen-I: 112; FN/Collagen-I: 164. **B/C)** Volcano plots demonstrating enrichment of proteins identified on **(B)** fibronectin (right) and collagen-I (left) matrices and **(C)** fibronectin (right) and LAP (left) matrices. Pairwise statistical analysis: Fisher's exact test; quantitative method: weighted spectra; significance level: $P < 0.05$. Significant proteins (dark grey); non-significant proteins (light grey); proteins of interest highlighted in purple. **D/E)** Visual representation of ClueGO cellular compartment gene ontology (GO) analyses of proteins significantly enriched on **(D)** Fibronectin in comparison with Collagen-I, or **(E)** LAP in comparison with Fibronectin in BT474 cells. Colours represent specific merged GO term groups, node size represents level of significance of each GO term, clustering and edge length represent functionally grouped networks based on kappa score. All mass spectrometry data represent 3 independent experiments.

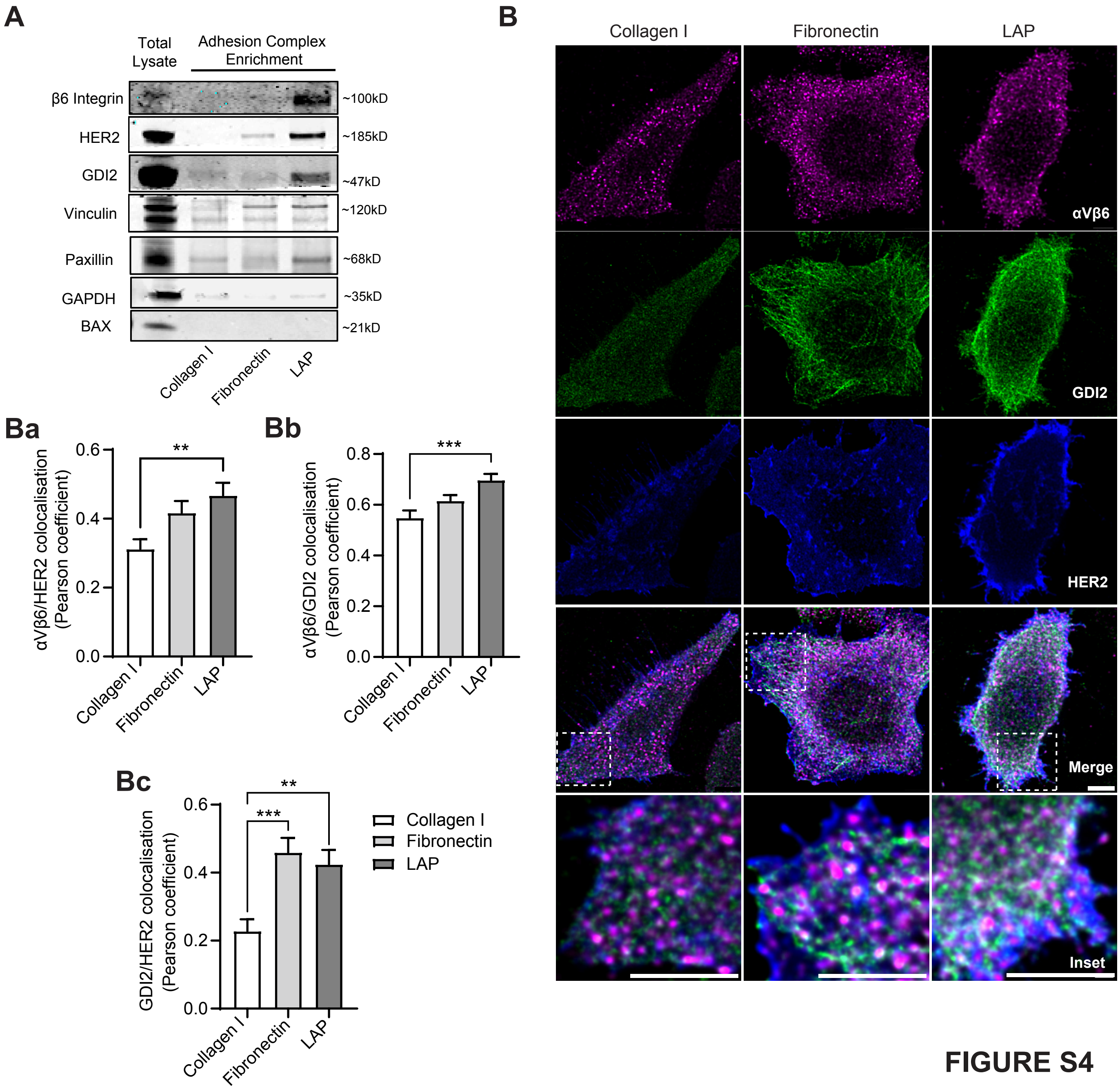
A
Total Lysate
Adhesion Complex Enrichment
β6 Integrin
HER2
GDI2
Vinculin
Paxillin
GAPDH
BAX
~100kD
~185kD
~47kD
~120kD
~68kD
~35kD
~21kD
Collagen I
Fibronectin
LAP
B
Collagen I
Fibronectin
LAP
αVβ6
GDI2
HER2
Merge
Inset
Ba
αVβ6/HER2 colocalisation (Pearson coefficient)
**
Bb
αVβ6/GDI2 colocalisation (Pearson coefficient)
***
Bc
GDI2/HER2 colocalisation (Pearson coefficient)
***
**
Collagen I
Fibronectin
LAP

FIGURE S4

Figure S4: Ligand-engagement of integrin $\alpha_V\beta_6$ promotes recruitment of, and colocalisation with, HER2 and GDI2. A) Immunoblot analysis of β_6 integrin, HER2, GDI2, Vinculin, Paxillin, GAPDH and BAX protein levels in IACs isolated from BT474 cells on LAP ($\alpha_V\beta_6$-selective ligand), fibronectin ($\alpha_V\beta_6$-binding non-selective ligand) and collagen-I (non-$\alpha_V\beta_6$-binding ligand). **B)** Immunofluorescent staining of $\alpha_V\beta_6$ integrin (magenta), GDI2 (green) and HER2 (blue) in HER2-18 cells attached to collagen-I, fibronectin and LAP. Images show the basal Z-plane representing the cell-matrix interface. Dashed boxes indicate location of insets; scale bar represents 5 µm. **Ba/b/c)** Quantitative analysis of colocalisation calculated by Pearson's coefficient ± S.E.M of **(Ba)** $\alpha_V\beta_6$/HER2 colocalisation, **(Bb)** $\alpha_V\beta_6$/GDI2 colocalisation, and **(Bc)** GDI2/HER2 colocalisation. *N* = 3 independent replicate experiments with 24 images analysed per condition. One-Way ANOVA test, followed by a Tukey's multiple comparisons test: **(Ba)** $\alpha_V\beta_6$/HER2 colocalisation Collagen-I vs LAP *P* = 0.0046; **(Bb)** $\alpha_V\beta_6$/GDI2 colocalisation Collagen-I vs LAP *P* = 0.0004; **(Bc)** GDI2/HER2 colocalisation Collagen-I vs Fibronectin *P* = 0.0004, Collagen-I vs LAP *P* = 0.0028.

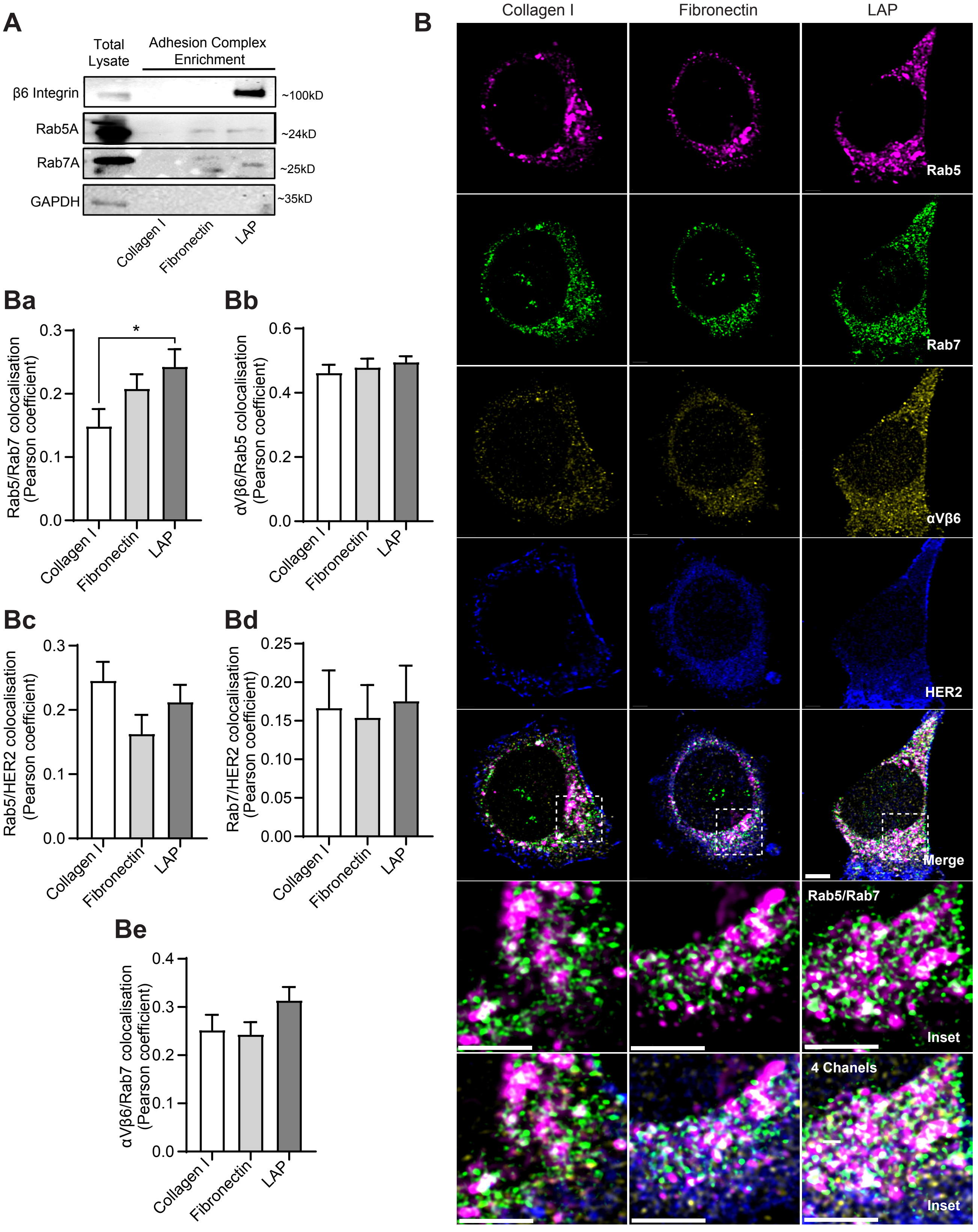

A
Total Lysate
Adhesion Complex Enrichment
β6 Integrin
Rab5A
Rab7A
GAPDH
~100kD
~24kD
~25kD
~35kD
Collagen I
Fibronectin
LAP
Ba
Rab5/Rab7 colocalisation (Pearson coefficient)
Bb
αVβ6/Rab5 colocalisation (Pearson coefficient)
Bc
Rab5/HER2 colocalisation (Pearson coefficient)
Bd
Rab7/HER2 colocalisation (Pearson coefficient)
Be
αVβ6/Rab7 colocalisation (Pearson coefficient)
B
Rab5
Rab7
αVβ6
HER2
Merge
Rab5/Rab7
Inset
4 Chanels

FIGURE S5

Figure S5: Ligand-engagement of integrin $\alpha_V\beta_6$ promotes recruitment of RAB5 and RAB7 to IACs and increases RAB5/RAB7 colocalisation. A) Immunoblot analysis of β_6 integrin, RAB5A, RAB7A and GAPDH protein levels in IACs isolated from HER2-18 cells on LAP ($\alpha_V\beta_6$-selective ligand), fibronectin ($\alpha_V\beta_6$-binding non-selective ligand) and collagen-I (non-$\alpha_V\beta_6$-binding ligand). **B)** Immunofluorescent staining of RAB5 (magenta), RAB7 (green), $\alpha_V\beta_6$ integrin (yellow) and HER2 (blue) in HER2-18 cells attached to collagen-I, fibronectin and LAP. Images show central Z-planes representing regions containing endosomal structures. Dashed boxes indicate location of insets; scale bar represents 5 µm. **Ba/b/c/d/e)** Quantitative analysis of colocalisation calculated by Pearson's coefficient ± S.E.M of **(Ba)** RAB5/RAB7, **(Bb)** $\alpha_V\beta_6$/RAB5, **(Bc)** RAB5/HER2, (**Bd)** RAB7/HER2, and **(Be)** $\alpha_V\beta_6$/RAB7 colacalisation. *N* = 3 independent replicate experiments with 24 images analysed per condition. One-Way ANOVA test, followed by a Tukey's multiple comparisons test: **(Ba)** $\alpha_V\beta_6$ and RAB5/RAB7 colocalisation Collagen I vs LAP *P* = 0.0327.

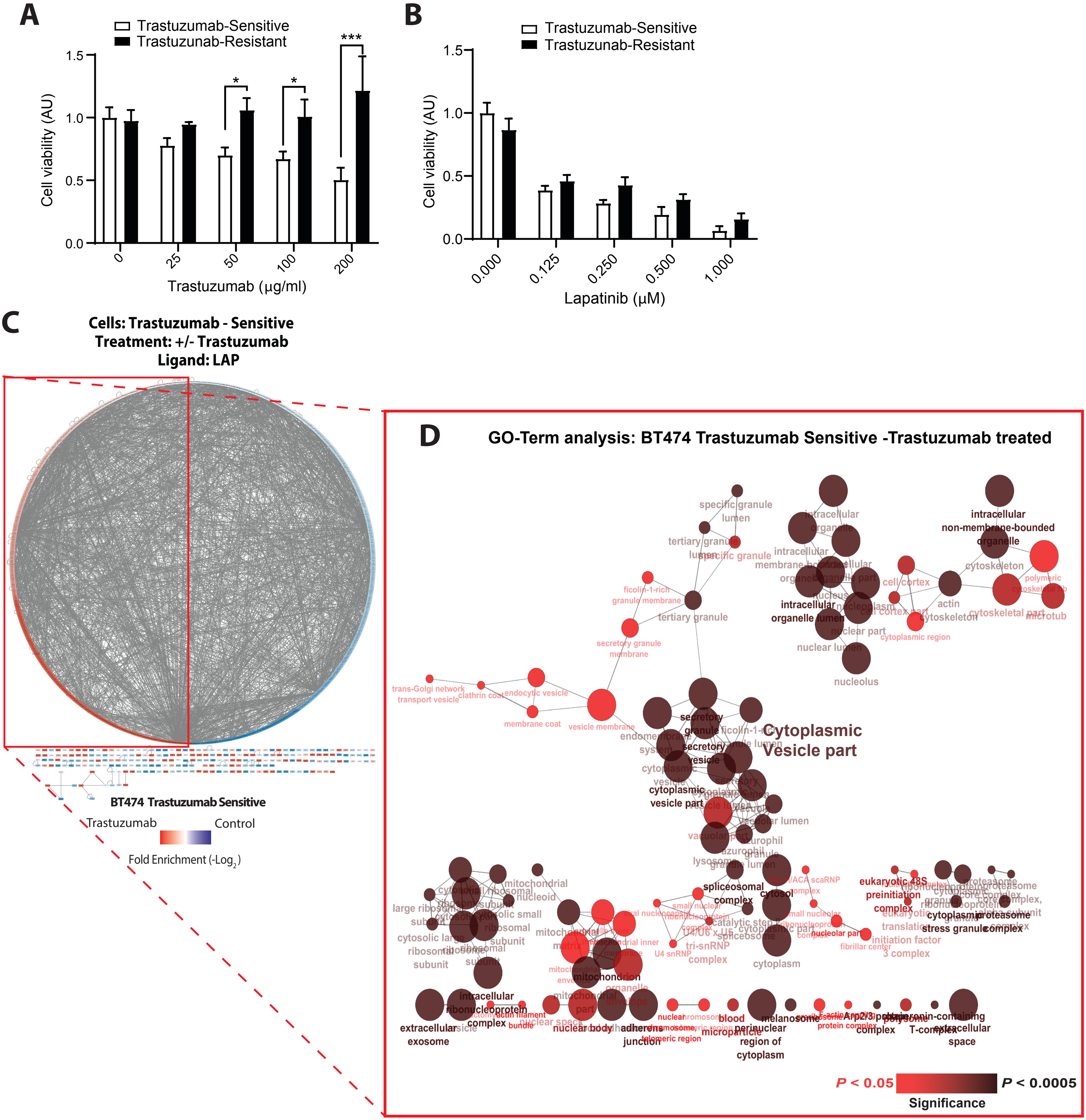
A
Trastuzumab-Sensitive
Trastuzunab-Resistant
Cell viability (AU)
1.5
1.0
0.5
0.0
*
*
***
0
25
50
100
200
Trastuzumab (µg/ml)
B
Trastuzumab-Sensitive
Trastuzunab-Resistant
Cell viability (AU)
0.000
0.125
0.250
0.500
1.000
Lapatinib (µM)
C
Cells: Trastuzumab - Sensitive
Treatment: +/- Trastuzumab
Ligand: LAP
BT474 Trastuzumab Sensitive
Trastuzumab
Control
Fold Enrichment (-Log2)
D
GO-Term analysis: BT474 Trastuzumab Sensitive -Trastuzumab treated
Cytoplasmic
Vesicle part
intracellular
non-membrane-bounded
organelle
intracellular
organelle lumen
nuclear lumen
nucleolus
cytoskeleton
actin
cytoskeleton
cytoplasmic region
secretory granule
membrane
vesicle membrane
membrane coat
trans-Golgi network
transport vesicle
clathrin coat
endocytic vesicle
tertiary granule
specific granule
lumen
spliceosomal
complex
cytosol
cytoplasm
eukaryotic 48S
preinitiation
complex
stress granule
initiation factor
3 complex
mitochondrion
large ribosomal
subunit
cytosolic large
ribosomal
subunit
intracellular
ribonucleoprotein
complex
extracellular
exosome
nuclear body
adherens
junction
melanosome
perinuclear
region of
cytoplasm
extracellular
space
T-complex
P < 0.05
P < 0.0005
Significance

FIGURE S6

Figure S6: Pre-treatment with sub-lethal concentrations of trastuzumab alters the composition of $\alpha_V\beta_6$-dependent adhesion complexes. A/B) Cell viability assay (MTS) for Trastuzumab-Sensitive and Trastuzumab-Resistant BT474 cells. Cells were treated with different concentrations of **(A)** trastuzumab (0, 25, 50, 100 and 200 µg/mL), or **(B)** Lapatinib (0, 0.125, 0.250, 0.50 and 1.000 µM), for 48 hours. Data are arbitrary units (AU) normalised to control means ± S.E.M. from 4 independent experiments. Two-way ANOVA with Tukey test for multiple comparisons: Trastuzumab-Sensitive vs Trastuzumab-Resistant 50 µg/mL $P = 0.0361$; 100 µg/mL $P = 0.0483$; 200 µg/mL $P = 0.0001$. Statistical significance $^*P < 0.05$, $^{***}P < 0.001$. **C/D)** IAC enrichment coupled with free-label mass spectrometry was used to define proteins specifically recruited to ligand-bound $\alpha_V\beta_6$ in trastuzumab-sensitive BT474 cells treated with trastuzumab (10 µg/mL) for 4 days prior to plating on LAP. **C)** Protein-protein interaction network of proteins significantly enriched in $\alpha_V\beta_6$-mediated adhesion complexes of trastuzumab-sensitive cells pre-treated with trastuzumab (red nodes) or vehicle control (blue nodes). Lines (edges) linking nodes represent protein-protein interactions. **D)** Visual representation of ClueGO cellular compartment GO analyses of proteins significantly enriched in $\alpha_V\beta_6$-mediated complexes of trastuzumab-sensitive BT474s treated with trastuzumab, relative to untreated controls. Node size represents number of mapped proteins in each GO term, colour indicates level of significance of each GO term, node clustering and edge length represent functionally grouped networks based on kappa score. All mass spectrometry experiments represent the average of 3 independent experiments. Data relate to **Figure 3D-F**.

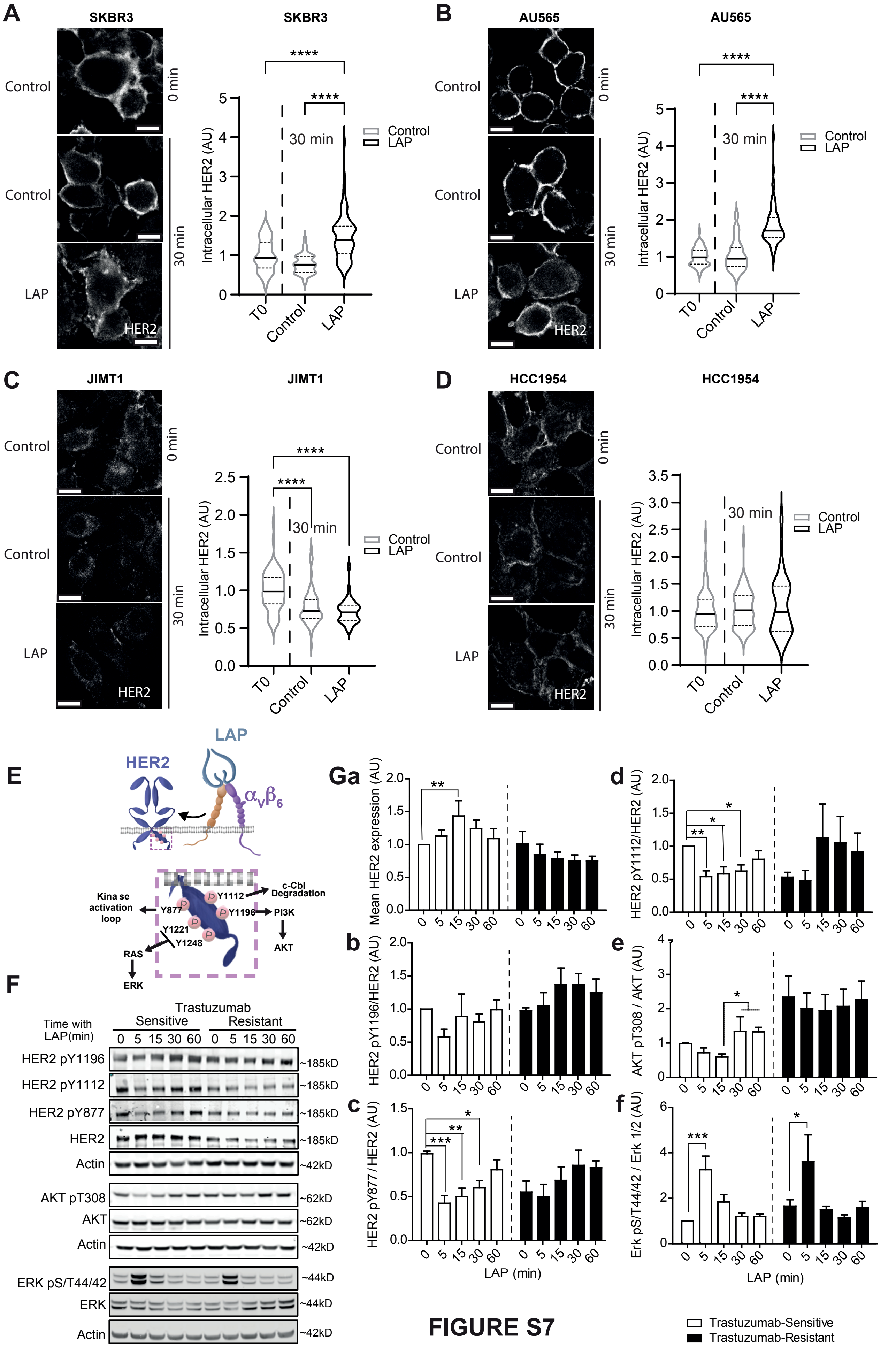
A
SKBR3
Control
Control
LAP
HER2
0 min
30 min
Intracellular HER2 (AU)
30 min
Control
LAP
T0
B
AU565
C
JIMT1
D
HCC1954
E
LAP
HER2
$\alpha_V\beta_6$
Y1112
c-Cbl Degradation
Kinase activation loop
Y877
Y1196
PI3K
AKT
Y1221
Y1248
RAS
ERK
F
Time with LAP(min)
Trastuzumab
Sensitive
Resistant
HER2 pY1196
HER2 pY1112
HER2 pY877
HER2
Actin
AKT pT308
AKT
ERK pS/T44/42
ERK
~185kD
~42kD
~62kD
~44kD
Ga
Mean HER2 expression (AU)
b
HER2 pY1196/HER2 (AU)
c
HER2 pY877 / HER2 (AU)
d
HER2 pY1112/HER2 (AU)
e
AKT pT308 / AKT (AU)
f
Erk pS/T44/42 / Erk 1/2 (AU)
LAP (min)
Trastuzumab-Sensitive
Trastuzumab-Resistant

FIGURE S7

Figure S7: Ligand-induced αVβ6 stimulation triggers internalisation of HER2 in trastuzmab sensitive cells and differential time-dependent regulation of HER2 expression, phosphorylation and MAPK/AKT activity. A-D) Affibody-chase experiments. Cells were surface-labelled with FITC-conjugated HER2 affibody and stimulated with soluble LAP (LAP), to stimulate $\alpha_V\beta_6$ integrin and trigger $\alpha_V\beta_6$ endocytosis, or vehicle control (Control), for 0 or 30 minutes. Quantitation represents intracellular HER2 fluorescence intensity analysis in **(A/B)** trastuzumab-sensitive SKBR3 and AU565 cells, or **(C/D)** trastuzumab-resistant JIMT1 and HCC1954 cells. Data are arbitrary units (AU) normalised to control means ± S.E.M. of 3 independent experiments 68-79 cells per condition, normalised to T0 (0 min) control scale bar= 10 µm. One-way ANOVA with Tukey's multiple comparison test: **(A)** SKBR3 cells 0 min (T0) vs LAP 30 min *P* < 0.0001, Control 30 min vs LAP 30 min *P* < 0.0001; (**B)** AU565 cells 0 min (T0) vs LAP 30 min *P* < 0.0001, Control 30 min vs LAP 30 min *P* < 0.0001; (**C)** JIMT1 cells 0 min (T0) vs Control 30 min *P* < 0.0001, 0 min (T0) LAP 30 min *P* < 0.0001; Control 30 min vs LAP 30 min non-significant; (**D)** HCC1954 cells 0 min (T0) vs Control 30 min non-significant, 0 min (T0) vs LAP 30 min non-significant; Control 30 min vs LAP 30 min non-significant. **E)** Schematic diagram showing HER2 phosphorylation sites and the functional consequences of specific post-translational modifications on HER2 function, proteostasis and downstream signalling. **F/G)** Trastuzumab-Sentitive and Trastuzumab-Resistant BT474 cells were treated with soluble LAP for 0, 5, 15, 30 and 60 minutes. **(F)** Representative blots for HER2, HER2 pY1112, HER2 pY1196, HER2 pY877, AKT, AKT pT308, ERK1/2, ERK1/2 pS/T 44/42 and Actin. **(G)** Quantitative analysis of: **(Ga)** HER2 total expression, relative to actin; **(Gb)** HER2 phosphorylation levels of pY1196, **(Gc)** pY877, and **(Gd)** pY1112 relative to total HER2; **(Ge)** AKT phosphorylation (pT308), relative to total AKT; **(Gf)** ERK1/2 phosphorylation (pS/T 44/42), relative to total ERK1/2. All data were normalised to 0 min of trastuzumab-sensitive cells and are presented as Mean ± SEM of 8 independent experiments. One-way ANOVA with Dunnett's multiple comparison test: **(Ga)** Trastuzumab-Sensitive 0 vs 15 min *P* = 0.0089; **(Gc)** Trastuzumab-Sensitive 0 vs 5 min *P* = 0.0002, 0 vs 15 min *P* = 0.0013, 0 vs 30 min *P* = 0.0111; **(Gd)** Trastuzumab-Sensitive 0 vs 5 *P* = 0.0064, 0 vs 15 min *P* = 0.0143, 0 vs 30 min *P* = 0.0301; **(Ge)** Trastuzumab-Sensitive 15 vs 30 min *P* = 0.340, 15 vs 60 min *P* = 0.0117; **(Gf)** Trastuzumab-Sensitive 0 vs 5 *P* = 0.0001, Trastuzumab-Resistant 0 vs 5 *P* = 0.0197. **A-G)** Statistical significance is denoted with asterisks: **P* < 0.05, ***P* < 0.01, ****P* < 0.001.

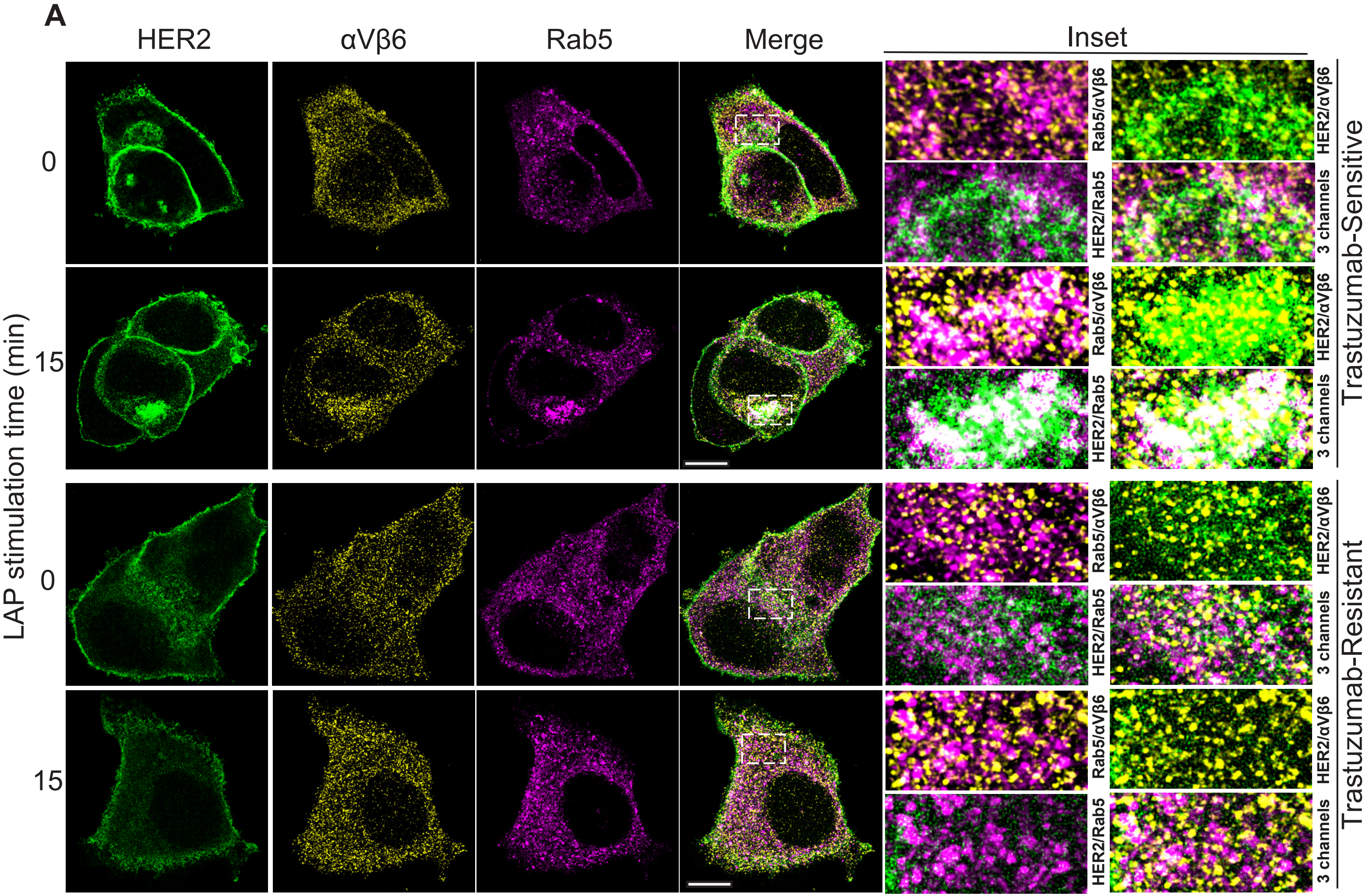
A
HER2
αVβ6
Rab5
Merge
Inset
LAP stimulation time (min)
0
15
0
15
Rab5/αVβ6
HER2/αVβ6
HER2/Rab5
3 channels
Trastuzumab-Sensitive
Trastuzumab-Resistant

FIGURE S8

Figure S8: HER2, RAB5 and integrin $\alpha_V\beta_6$ colocalise in trastuzumab-sensitive cells following LAP stimulation. A) Representative immunofluorescence images demonstrating colocalisation of HER2 (green), integrin $\alpha_V\beta_6$ (yellow) and RAB5 (magenta) in trastuzumab-sensitive and trastuzumab-resistant BT474 cells, treated with soluble LAP for 0 and 15 minutes. Scale bar = 10 µm. Dashed boxes indicate location of insets, highlighting intracellular cytoplasmic regions. For each representative cell image, four versions of each inset are presented to demonstrate levels of Rab5/$\alpha_V\beta_6$, HER2/$\alpha_V\beta_6$, HER2/Rab5 and Rab5/$\alpha_V\beta_6$/HER2 co-localisation. Micrographs represent the same images from **Figure 4C**, demonstrating HER2 and RAB5 subcellular distribution, further analysed to demonstrate 3-channel colocalisation with $\alpha_V\beta_6$.

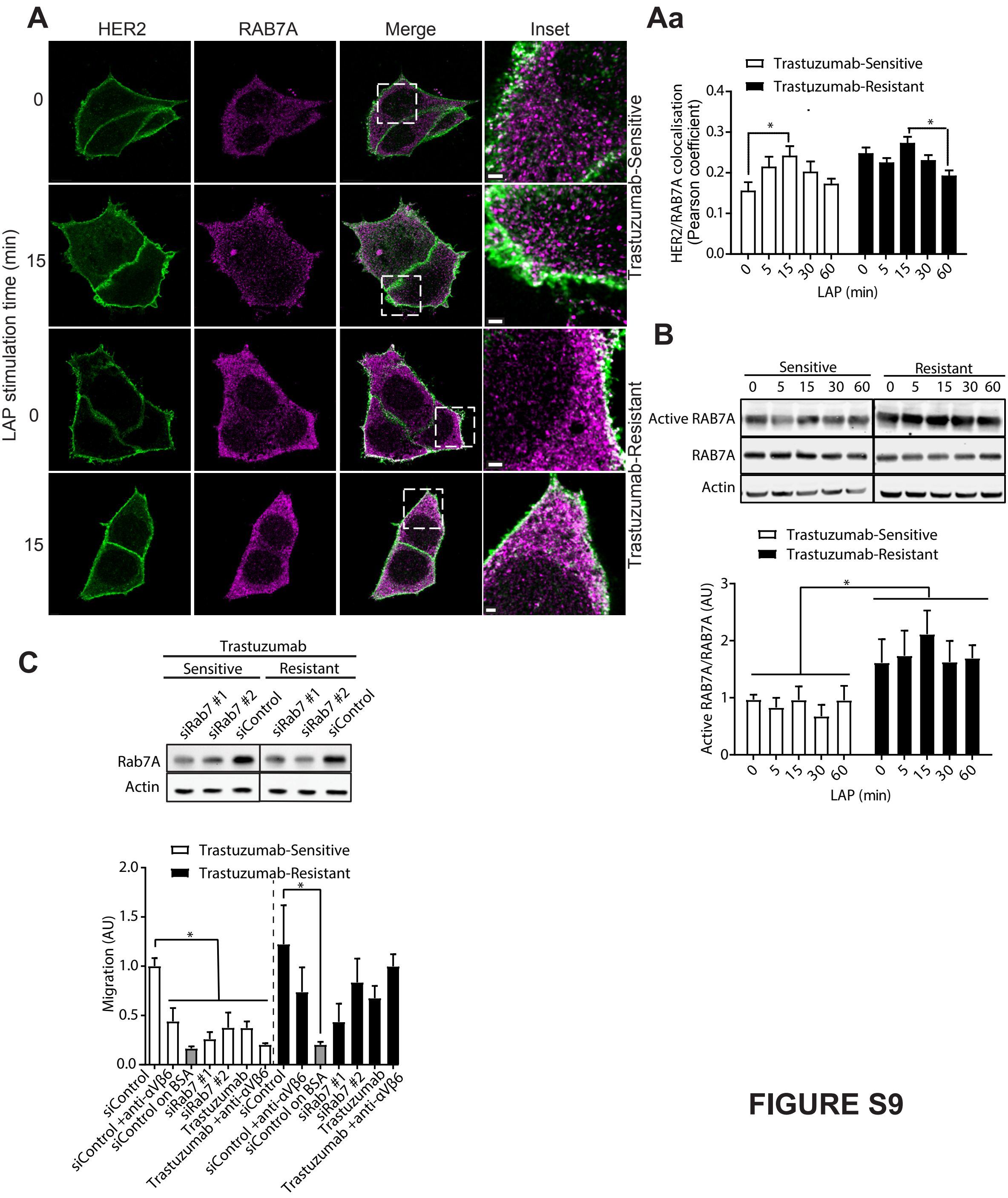

A
HER2
RAB7A
Merge
Inset
LAP stimulation time (min)
0
15
0
15
Trastuzumab-Sensitive
Trastuzumab-Resistant
Aa
Trastuzumab-Sensitive
Trastuzumab-Resistant
HER2/RAB7A colocalisation (Pearson coefficient)
0.0
0.1
0.2
0.3
0.4
*
*
0 5 15 30 60 0 5 15 30 60
LAP (min)
B
Sensitive
Resistant
0 5 15 30 60 0 5 15 30 60
Active RAB7A
RAB7A
Actin
Trastuzumab-Sensitive
Trastuzumab-Resistant
Active RAB7A/RAB7A (AU)
0
1
2
3
*
0 5 15 30 60 0 5 15 30 60
LAP (min)
C
Trastuzumab
Sensitive
Resistant
siRab7 #1
siRab7 #2
siControl
siRab7 #1
siRab7 #2
siControl
Rab7A
Actin
Trastuzumab-Sensitive
Trastuzumab-Resistant
Migration (AU)
0.0
0.5
1.0
1.5
2.0
*
*
siControl
siControl +anti-αVβ6
siControl on BSA
siRab7 #1
siRab7 #2
Trastuzumab
Trastuzumab +anti-αVβ6
siControl
siControl +anti-αVβ6
siControl on BSA
siRab7 #1
siRab7 #2
Trastuzumab
Trastuzumab +anti-αVβ6

FIGURE S9

Figure S9: RAB7A colocalises with internalised HER2, is activated in trastuzumab resistant cells and is required for αVβ6- and HER2-dependent migration. A) Colocalisation analysis of HER2 (green) and RAB7A (magenta) immunofluorescence for in trastuzumab-sensitive and -resistant BT474 cells, treated with soluble LAP for 0, 5, 15, 30 and 60 minutes (N = 3, 16-28 cells per condition). **Aa)** Quantitative analysis of HER2/RAB7A colocalisation calculated by Pearson's coefficient. Two-way ANOVA with Tukey's multiple comparison test: Trastuzumab-Sensitive 0 vs 15 min P = 0.0152; Trastuzumab-Resistant 15 vs 60 min P = 0.0174. **B)** Representative blots of RAB7A activity during a time-course of LAP stimulation. Active RAB7 (RAB7-GTP): detection of RAB7A in GST-RILP pulldown eluate; total RAB7A expression in total cell lysate. Graph showing mean RAB7A activity, relative to total RAB7A ± S.E.M normalised to Trastuzumab-Sensitive cells 0 min. N = 3 independent replicate experiments. One-way ANOVA with Dunnett's multiple comparison test: P = 0.0356. The immunoblot images separated by a vertical black line, are from the same image of the membrane. The image was split to present Trastuzumab-Sensitive data on the left and Trastuzumab-Resistant data on the right, to maintain consistency in data presentation. **C)** Haptotactic migration analysis of BT474 cells (Trastuzumab-Sensitive and Trastuzumab-Resistant) in Transwells coated with fibronectin, or BSA as negative control. Cells were transfected with siRNA against RAB7A (siRAB7 #1 and siRAB7 #2) or siRNA control. Migration was assessed over 24 hrs in the presence or absence $\alpha_V\beta_6$ integrin blocking antibody or Trastuzumab. Data shown are mean ± S.E.M. of 4 independent experiments. Welch's ANOVA with Dunnett's multiple comparison test: Trastuzumab-Sensitive cells: siControl vs siControl + BSA P = 0.0281, siControl vs siControl + anti-$\alpha_V\beta_6$ P = 0.0285, siControl vs siRAB7 1 P = 0.0156, siControl vs siRAB7 2 P = 0.0309, siControl vs Trastuzumab P = 0.0311, siControl vs Trastuzumab + anti-$\alpha_V\beta_6$ P = 0.0307. Trastuzumab-Resistant cells: siControl vs BSA P = 0.0151. **B/C)** Data are arbitrary units (AU) normalised to control means ± S.E.M. Statistical significance *P <0.05.

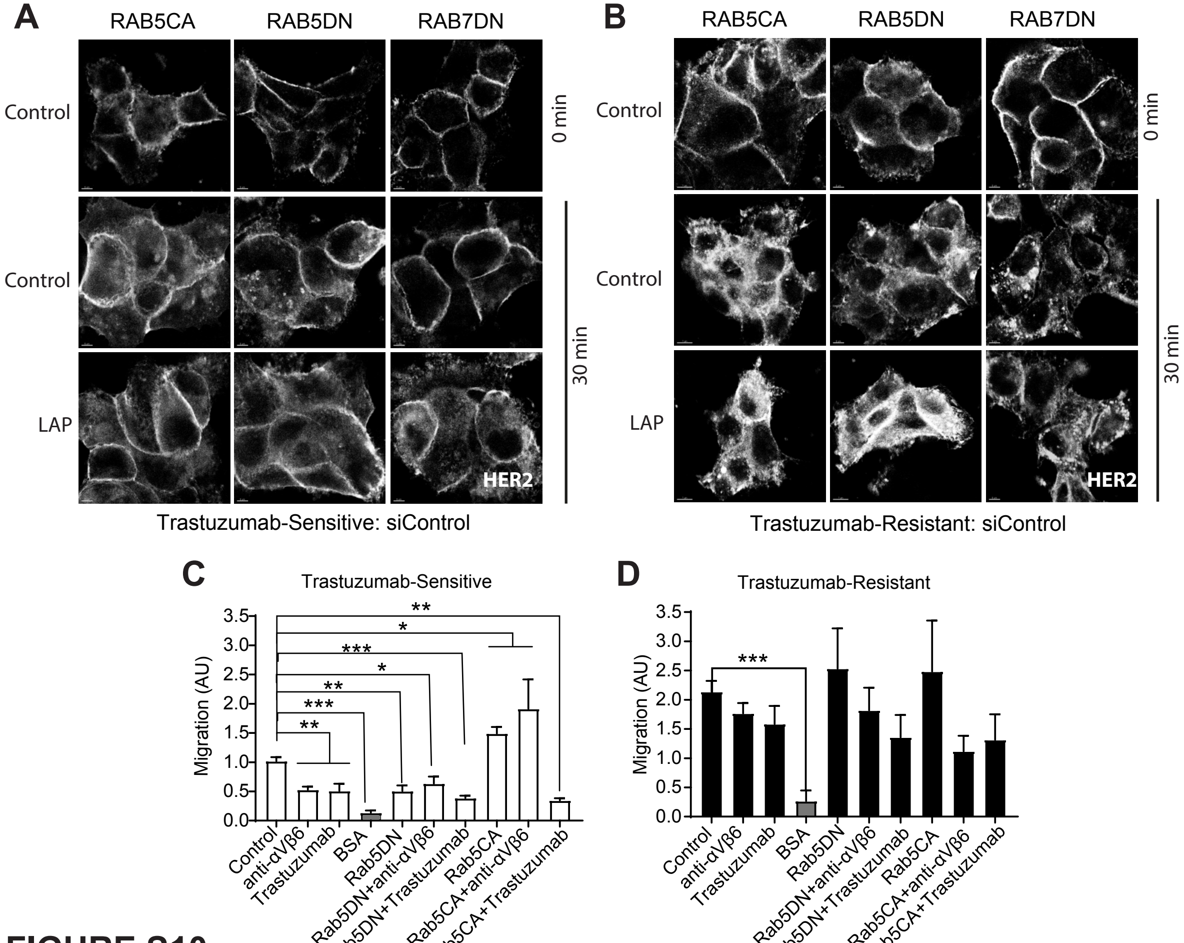
A
RAB5CA
RAB5DN
RAB7DN
Control
Control
LAP
0 min
30 min
HER2
Trastuzumab-Sensitive: siControl
B
RAB5CA
RAB5DN
RAB7DN
Control
Control
LAP
0 min
30 min
HER2
Trastuzumab-Resistant: siControl
C
Trastuzumab-Sensitive
Migration (AU)
0.0
0.5
1.0
1.5
2.0
2.5
3.0
3.5
**
*
***
*
**
***
**
Control
anti-αVβ6
Trastuzumab
BSA
Rab5DN
Rab5DN+anti-αVβ6
Rab5DN+Trastuzumab
Rab5CA
Rab5CA+anti-αVβ6
Rab5CA+Trastuzumab
D
Trastuzumab-Resistant
Migration (AU)
0.0
0.5
1.0
1.5
2.0
2.5
3.0
3.5
***
Control
anti-αVβ6
Trastuzumab
BSA
Rab5DN
Rab5DN+anti-αVβ6
Rab5DN+Trastuzumab
Rab5CA
Rab5CA+anti-αVβ6
Rab5CA+Trastuzumab

FIGURE S10

Figure S10: RAB5 & RAB7 regulate LAP-dependent internalisation and intracellular accumulation of HER2. A/B) Representative images of affibody-chase experiments in **(A)** Trastuzumab-Sensitive and **(B)** Trastuzumab-Resistant BT474 cells. Images correspond to quantitative data presented in **Figure 4E/F**. Images represent cells transfected with siRNA Control (siControl) and co-transfected with constitutively active RAB5 (RAB5Q79L-mCherry, RAB5CA), dominant negative RAB5 (RAB5S34N-mcherry, RAB5DN), or dominant negative RAB7 (RAB7T22N-mcherry, RAB7DN). Cells were surface-labelled with FITC-conjugated HER2 affibody and stimulated with soluble LAP (LAP), to stimulate $\alpha_V\beta_6$ integrin and trigger $\alpha_V\beta_6$ endocytosis, or vehicle control (control), for 0 or 30 minutes. Due to the reduced level of cell-surface HER2 labelling in trastuzumab-resistant cells, the image intensity is increased in panel **B** compared with panel **A**, to highlight the differences in intracellular accumulation. For further analyses see **Supplementary Results** and **Supplementary Figure S11A-D**. **C/D)** Haptotactic migration analysis of **(C)** Trastuzumab-Sensitive and **(D)** Trastuzumab-Resistant BT474 cells in Transwells coated with fibronectin, or BSA as negative control. Cells were transfected with constitutively active RAB5 (RAB5Q79L-mCherry), dominant negative RAB5 (RAB5S34N-mcherry) or empty mCherry vector. Migration was assessed over 24 hrs in the presence or absence of $\alpha_V\beta_6$ integrin blocking antibody or Trastuzumab. Data are arbitrary units (AU) normalised to control means ± S.E.M. of 4 independent experiments. One-way ANOVA with Welch's multiple comparison: Trastuzumab sensitive cells control vs $\alpha_V\beta_6$ antibody $P = 0.005$; Trastuzumab and Rab5DN constructs $P = 0.0013$; Control vs Rab5CA $P = 0.0298$; control vs Rab5CA+$\alpha_V\beta_6$ antibody $P = 0.0298$; control vs Rab5CA+Trastuzumab $P = 0.0009$; Control vs BSA $P = 0.0002$. Statistical significance *$P < 0.05$, **$P < 0.01$, ***$P < 0.001$.

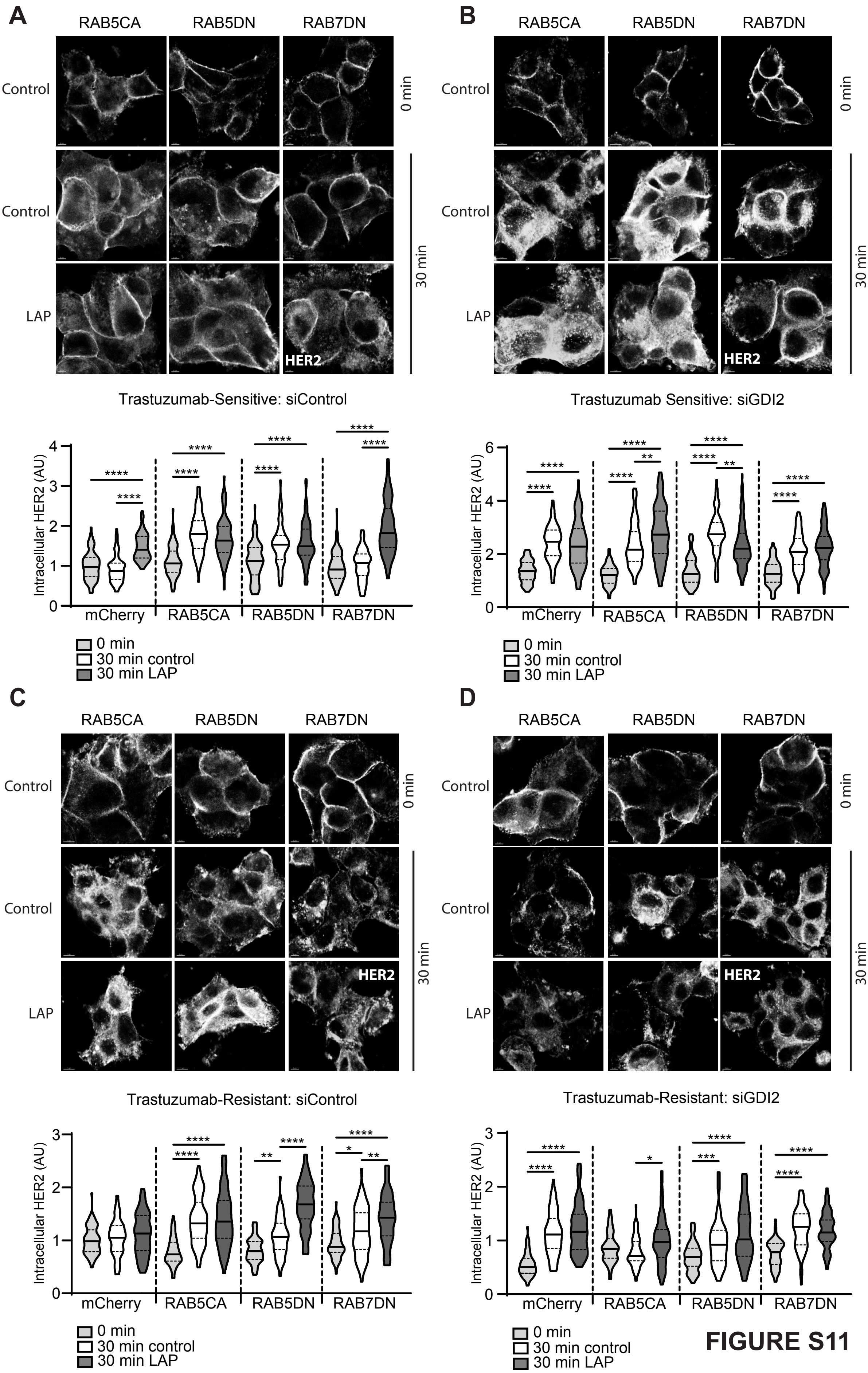
A
RAB5CA
RAB5DN
RAB7DN
Control
Control
LAP
0 min
30 min
HER2
Trastuzumab-Sensitive: siControl
Intracellular HER2 (AU)
mCherry
RAB5CA
RAB5DN
RAB7DN
0 min
30 min control
30 min LAP
B
RAB5CA
RAB5DN
RAB7DN
Control
Control
LAP
0 min
30 min
HER2
Trastuzumab Sensitive: siGDI2
Intracellular HER2 (AU)
mCherry
RAB5CA
RAB5DN
RAB7DN
0 min
30 min control
30 min LAP
C
RAB5CA
RAB5DN
RAB7DN
Control
Control
LAP
0 min
30 min
HER2
Trastuzumab-Resistant: siControl
Intracellular HER2 (AU)
mCherry
RAB5CA
RAB5DN
RAB7DN
0 min
30 min control
30 min LAP
D
RAB5CA
RAB5DN
RAB7DN
Control
Control
LAP
0 min
30 min
HER2
Trastuzumab-Resistant: siGDI2
Intracellular HER2 (AU)
mCherry
RAB5CA
RAB5DN
RAB7DN
0 min
30 min control
30 min LAP

FIGURE S11

Figure S11: GDI2 regulated RAB5 activity to control αVβ6-dependent HER2 endocytosis and cell migration. A/B) Affibody-chase experiments in Trastuzumab-Sensitive and Trastuzumab-Resistant BT474 cells transfected with siRNA against GDI2 (siGDI2#1) or siRNA Control (siControl) and co-transfected with constitutively active RAB5 (RAB5Q79L-mCherry, RAB5CA), dominant negative RAB5 (RAB5S34N-mcherry, RAB5DN), dominant negative RAB7 (RAB7T22N-mcherry, RAB7DN) or empty mCherry vector. Cells were surface-labelled with FITC-conjugated HER2 affibody and stimulated with soluble LAP (LAP), to stimulate $\alpha_V\beta_6$ integrin and trigger $\alpha_V\beta_6$ endocytosis, or vehicle control (control), for 0 or 30 minutes. Quantitation represents cytoplasmic HER2 fluorescence intensity analysis in **(A)** trastuzumab-sensitive siControl **(B)** trastuzumab-sensitive siGDI2 **(C)** trastuzumab-resistant siControl, and **(D)** trastuzumab-sensitive siGDI2 BT474 cells (*N* = 3; 70-86 cells per condition), scale bar = 5 µm. Statistical significance was assessed using a One-way ANOVA with Tukey test for multiple comparisons; Trastuzumab sensitive cells: **A)** siControl mCherry 0 min vs LAP 30 min *P* < 0.0001, 30 min Control vs 30 min LAP *P* < 0.0001, siControl RAB5CA 0 min vs Control 30 min *P* < 0.0001, 0 min vs LAP 30 min *P* < 0.0001. siControl RAB5DN 0 min vs Control 30 min *P* < 0.0001, 0 min vs LAP 30 min *P* < 0.0001. siControl RAB7DN 0 min vs Control 30 min *P* < 0.0001, 0 min vs LAP 30 min *P* < 0.0001. **B)** siGDI2 mCherry 0 min vs LAP 30 min *P* < 0.0001, 30 min Control vs 30 min LAP *P* < 0.0001, siGDI2 RAB5CA 0 min vs Control 30 min *P* < 0.0001, 0 min vs LAP 30 min *P* < 0.0001, 30 min Control vs 30 min LAP *P* = 0.0051.

siGDI2 RAB5DN 0 min vs Control 30 min *P* < 0.0001, 0 min vs LAP 30 min *P* < 0.0001, 30 min Control vs 30 min LAP *P* = 0.0083. siGDI2 RAB7DN 0 min vs Control 30 min *P* < 0.0001, 0 min vs LAP 30 min *P* < 0.0001. Trastuzumab resistant cells: siControl RAB5CA 0 min vs Control 30 min *P* < 0.0001, 0 min vs LAP 30 min *P* < 0.0001. siControl RAB5DN 0 min vs Control 30 min *P* = 0.0034 , 0 min vs LAP 30 min *P* < 0.0001. siControl RAB7DN 0 min vs Control 30 min *P* = 0.0233 , 0 min vs LAP 30 min *P* < 0.0001, 30 min Control vs 30 min LAP *P* = 0.0058. siGDI2 mCherry 0 min vs LAP 30 min *P* < 0.0001, 30 min Control vs 30 min LAP *P* < 0.0001, siGDI2 RAB5CA 30 min Control vs 30 min LAP *P* = 0.0316. siGDI2 RAB5DN 0 min vs Control 30 min *P* = 0.0008 , 0 min vs LAP 30 min *P* < 0.0001, siGDI2 RAB7DN 0 min vs Control 30 min *P* < 0.0001, 0 min vs LAP 30 min *P* < 0.0001. Data are arbitrary units (AU) normalised to control means ± S.E.M. Statistical significance **P* < 0.05, ***P* < 0.01, ****P* < 0.001, *****P* < 0.0001. Due to differences in cell-surface HER2 levels in the different conditions, baseline image intensity is different in each panel **(A, B ,C, D)**, to highlight differences in internalisation. Quantitative data in **Supplementary Figure S11A and S11C** are also presented in **Figure 6 E/F and Supplementary Figure S10A and S10B.**

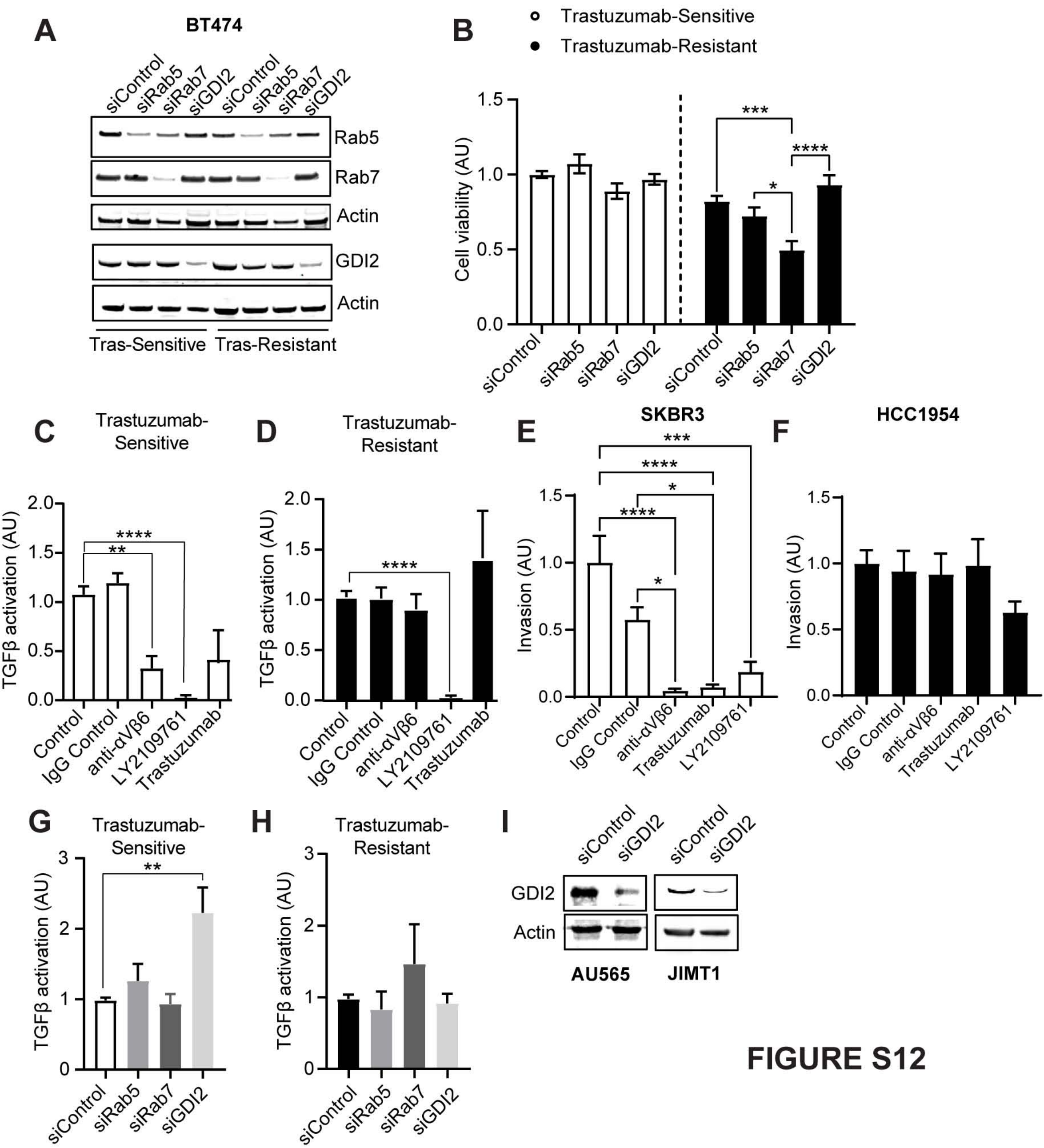
A
BT474
siControl
siRab5
siRab7
siGDI2
Rab5
Rab7
Actin
GDI2
Actin
Tras-Sensitive
Tras-Resistant
B
Trastuzumab-Sensitive
Trastuzumab-Resistant
Cell viability (AU)
C
Trastuzumab-Sensitive
TGFβ activation (AU)
Control
IgG Control
anti-αVβ6
LY2109761
Trastuzumab
D
Trastuzumab-Resistant
E
SKBR3
Invasion (AU)
F
HCC1954
G
Trastuzumab-Sensitive
H
Trastuzumab-Resistant
I
AU565
JIMT1
FIGURE S12

Figure S12: TGFβ activity is modulated by integrin αVβ6, HER2 and GDI2 and regulates invasive capacity in trastuzumab-sensitive, but not trastuzumab-resistant cells. A) Representative immunoblots of trastuzumab-sensitive and trastuzumab-resistant BT474 cells transfected with siRNA against RAB5A, RAB7A, GDI2 or control siRNA, probed for RAB5, RAB7, GDI2 and actin. **B)** Cell viability assay (MTS) for trastuzumab-sensitive and trastuzumab-resistant BT474 cells, transfected with siRNA against RAB5A, RAB7A, GDI2 or control siRNA. Data show mean ± S.E.M. from 5 independent experiments. Two-way ANOVA with Tukey test for multiple comparisons: Trastuzumab-Resistant siControl vs siRab7 $P < 0.001$, siRab5 vs siRab7 $P < 0.05$, siGDI2 vs siRab7 $P < 0.0001$. **C/D)** TGFβ activation assays with trastuzumab-sensitive **(C)** and -resistant **(D)** cells seeded on a monolayer of MLECs transfected with inducible TGFβ reporter, after 16 hours, induction of reporter was measured by luminescence. Cells were treated with trastuzumab (10 µg/mL), an inhibitory $\alpha_V\beta_6$ integrin antibody or a specific inhibitor for TGFβ receptor 1/2 (LY2109761, 10 µM) ($N = 4$). One-Way ANOVA with Tukey's multiples comparison test; significance **(C)** Control vs $\alpha_V\beta_6$ integrin antibody $P = 0.0004$, Control vs LY2109761 $P < 0.0001$ and **(D)** Control vs LY2109761 $P < 0.0001$. **E/F)** Invasion of trastuzumab-sensitive SKBR3 **(I)** and trastuzumab-resistant HCC1954 **(J)** cells in the presence or absence of $\alpha_V\beta_6$ integrin blocking antibody, 10 µg/mL trastuzumab, or a specific inhibitor for TGFβ receptor 1/2 (LY2109761, 10 µM) ($N = 3$). One-way ANOVA with Tukey's multiples comparisons test; **(E)** SKBR3 control vs $\alpha_V\beta_6$ antibody $P < 0.0001$, control vs trastuzumab $P < 0.0001$, control vs LY2109761 $P = 0.0001$, IgG control vs $\alpha_V\beta_6$ antibody $P = 0.0119$, IgG control vs trastuzumab $P = 0.0182$. **G/H)** TGFβ activation assays. TGFβ activation analysis of trastuzumab- sensitive **(G)** and -resistant **(H)** BT474 cells transfected with siRNA against RAB5, RAB7 and GDI2 or siRNA control and seeded on a monolayer of MLEC cells ($N = 4$). Kruskal-Wallis with Dunnett's multiples comparison test: **(G)** siControl vs siGDI2 $P = 0.006$. **I)** Representative immunoblots of trastuzumab-sensitive AU565 cells and endogenously trastuzumab-resistant JIMT1 cells transfected with siRNA against GDI2 or control siRNA, probed for GDI2 and actin. **B-H)** Data are arbitrary units (AU) normalised to control means ± S.E.M. **A-E)** Statistical significance $^{*}P < 0.05$, $^{**}P < 0.01$, $^{***}P < 0.001$, $^{****}P < 0.0001$.

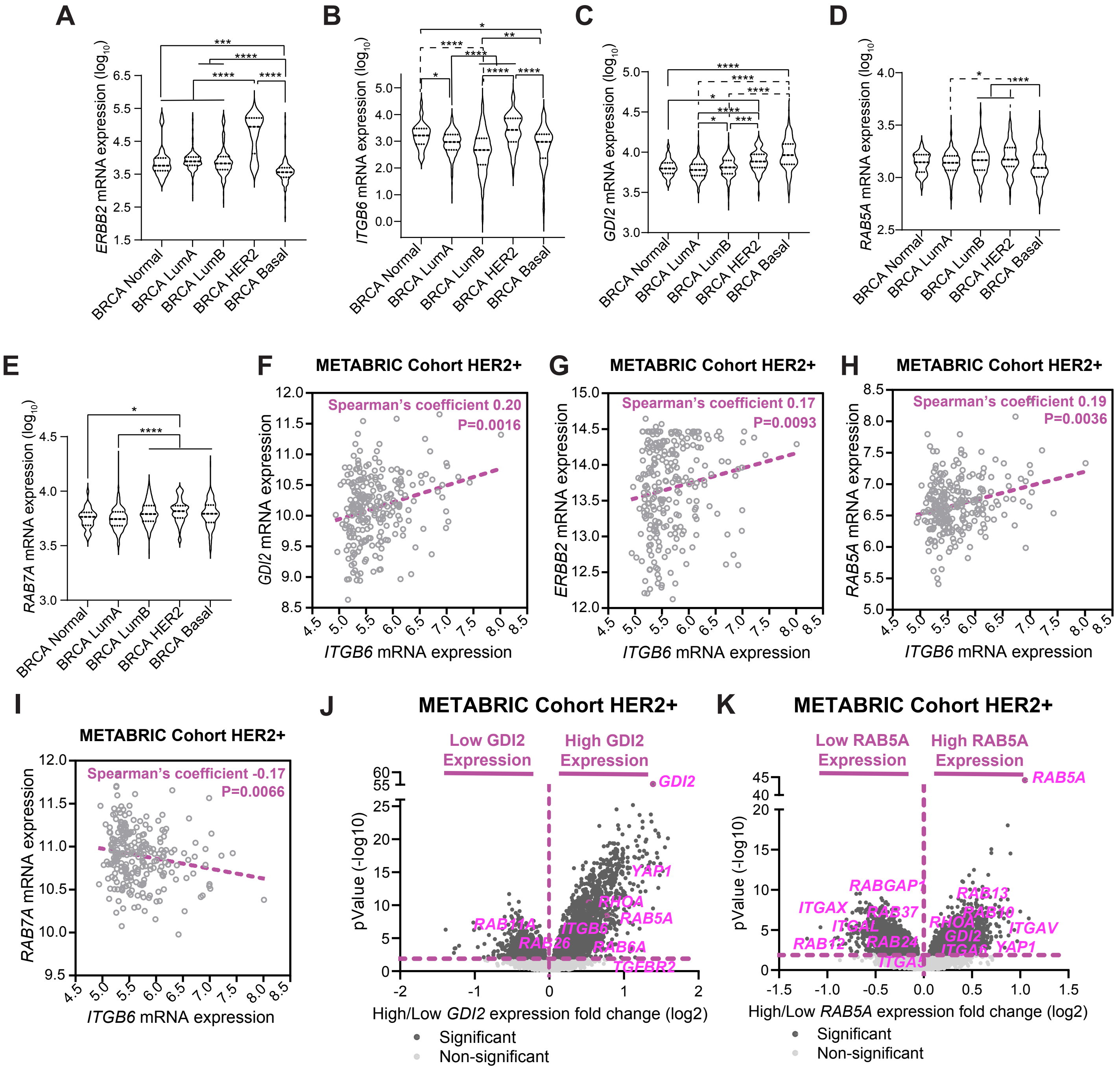
A
ERBB2 mRNA expression (log10)
BRCA Normal
BRCA LumA
BRCA LumB
BRCA HER2
BRCA Basal
B
ITGB6 mRNA expression (log10)
C
GDI2 mRNA expression (log10)
D
RAB5A mRNA expression (log10)
E
RAB7A mRNA expression (log10)
F
METABRIC Cohort HER2+
Spearman's coefficient 0.20
P=0.0016
GDI2 mRNA expression
ITGB6 mRNA expression
G
METABRIC Cohort HER2+
Spearman's coefficient 0.17
P=0.0093
ERBB2 mRNA expression
ITGB6 mRNA expression
H
METABRIC Cohort HER2+
Spearman's coefficient 0.19
P=0.0036
RAB5A mRNA expression
ITGB6 mRNA expression
I
METABRIC Cohort HER2+
Spearman's coefficient -0.17
P=0.0066
RAB7A mRNA expression
ITGB6 mRNA expression
J
METABRIC Cohort HER2+
Low GDI2 Expression
High GDI2 Expression
GDI2
YAP1
RHOA
RAB5A
RAB11A
ITGB6
RAB26
RAB6A
TGFBR2
pValue (-log10)
High/Low GDI2 expression fold change (log2)
Significant
Non-significant
K
METABRIC Cohort HER2+
Low RAB5A Expression
High RAB5A Expression
RAB5A
RABGAP1
RAB13
ITGAX
RAB37
RAB10
ITGAL
RHOA
ITGAV
GDI2
RAB12
RAB24
ITGA6
YAP1
ITGA5
pValue (-log10)
High/Low RAB5A expression fold change (log2)
Significant
Non-significant

FIGURE S13

Figure S13: *ERBB2*, *ITGB6* and *GDI2* mRNA expression are increased in the most aggressive subtypes of breast cancer. A-E) Analysis of RNA-seq gene expression for *ERBB2* **(A)**, *ITGB6* **(B)**, *GDI2* **(C)**, *RAB5A* **(D)** and *RAB7A* **(E)** in breast cancer patients (Breast Invasive Carcinoma TCGA, PanCancer Atlas data set). Data were extracted using the online resource https://www.cbioportal.org. Patients were subdivided, according to clinical diagnosis as: Normal-like (BRCA Normal), Luminal A (BRCA LumA), Luminal B (BRCA LumB), HER2+ (BRCA HER2+) and Basal-like (BRCA Basal); *n* = 981 patients. Dashed lines in violin blots represents the median and dotted lines represent the quartiles. Kruskal-Wallis with Dunn's multiple comparison test: **(A)** Normal vs HER2+ *P* < 0.0001, Normal vs. Basal *P* = 0.0002, LumA vs HER2+ *P* < 0.0001, LumB vs HER2+ *P* < 0.0001, LumB vs Basal *P* < 0.0001, HER2+ vs. Basal *P* < 0.0001; **(B)** Normal vs LumA *P* = 0.0352, Normal vs LumB *P* < 0.0001, Normal vs Basal *P* = 0.0127, LumA vs LumB *P* < 0.0001, LumA vs HER2+ *P* < 0.0001, LumB vs HER2+ *P* < 0.0001, LumB vs Basal *P* = 0.00084, HER2+ vs Basal *P* <0.0001; **(C)** Normal vs HER2+ *P* = 0.0100, Normal vs Basal *P* < 0.0001, LumA vs LumB *P* = 0.0220, LumA vs HER2+ *P* < 0.0001, LumA vs Basal *P* < 0.0001, LumB vs. HER2+ *P* = 0.0001, LumB vs Basal *P* < 0.0001; **(D)** LumA vs HER2+ *P* = 0.0293, LumB vs Basal *P* = 0.0008, HER2+ vs Basal *P* = 0.0003; **(E)** Normal vs HER2+ *P* = 0.0169, LumA vs LumB *P* < 0.0001, LumA vs HER2 *P* < 0.0001, LumA vs Basal *P* < 0.0001. **F-G)** Correlation analysis **(**Spearman's coefficient) of gene expression between *ITGB6* and *GDI2* **(F),** *ERBB2* **(G),** *RAB5A* **(H),** or *RAB7A* **(I)** in HER2+ breast cancers (*n* = 247 patients, METABRIC cohort). **J/K)** Volcano plots representing statistical analysis (ANOVA) of RNA-Seq gene expression data of HER2+ breast cancer patients from the METABRIC cohort expressing high (Q4, Right) and low (Q1, Left) levels of *GDI2* **(J)** and *RAB5A* **(K)**. Significant proteins (dark grey); non-significant proteins (light grey); proteins of interest highlighted in pink. Statistical significance **P* < 0.05, ***P* < 0.01 ****P* < 0.001, *****P* < 0.0001.

SUPPLEMENTARY AUXILLARY FILES

Supplementary File 1 - HER2-18 Adhesion Complex Enrichments – ECM
Spreadsheet summarising proteomic and ontological analysis of integrin-associated adhesion complexes isolated from HER2-18 cells plated on Collagen-1, Fibronectin and Latency Associated Peptide (LAP).
Worksheets summarise: Protein enrichment; Functional subnetwork (Clusters); GO-term analysis of Collagen-1 vs LAP, Collagen-1 vs Fibronectin; LAP vs Fibronectin.

Supplementary File 2 - BT474 Adhesion Complex Enrichments – ECM
Spreadsheet summarising proteomic and ontological analysis of integrin-associated adhesion complexes isolated from BT-474 cells (trastuzumab-sensitive) plated on Collagen-1, Fibronectin and Latency Associated Peptide (LAP).
Worksheets summarise: Protein enrichment; Functional subnetwork (Clusters); GO-term analysis of Collagen-1 vs LAP, Collagen-1 vs Fibronectin; LAP vs Fibronectin.

Supplementary File 3 - BT474 Tras-Sensitive vs Tras-Resistant +/- Trastuzumab - LAP IACs
Spreadsheet summarising proteomic and ontological analysis of $\alpha_V\beta_6$-dependent integrin-associated adhesion complexes isolated from trastuzumab-sensitive & trastuzumab-resistant BT-474 cells +/- pre-treatment with sub-lethal 10μg/ml trastuzumab, plated on Latency Associated Peptide (LAP).
Worksheets summarise: Protein enrichment; GO-term analysis of trastuzumab-sensitive vs trastuzumab-resistant, trastuzumab-sensitive vs +/- trastuzumab.
